# Characteristics of Existing Online Patient Navigation Interventions: Scoping Review

**DOI:** 10.2196/50307

**Published:** 2024-08-19

**Authors:** Meghan Marsh, Syeda Rafia Shah, Sarah E P Munce, Laure Perrier, Tin-Suet Joan Lee, Tracey J F Colella, Kristina Marie Kokorelias

**Affiliations:** 1 Department of Occupational Science and Occupational Therapy University of Toronto Toronto, ON Canada; 2 KITE Toronto Rehabiliation Institute Toronto, ON Canada; 3 Institute of Health Policy, Management and Evaluation University of Toronto Toronto, ON Canada; 4 Toronto Western Hospital University Health Network Toronto, ON Canada; 5 Section of Geriatrics Sinai Health and University Health Network Toronto, ON Canada

**Keywords:** online, patient navigation, peer navigation, patient navigation interventions, online patient navigation interventions, scoping review, patient portals, social care services, online medical tools, eHealth, telehealth, personal support, social care, patient navigation intervention

## Abstract

**Background:**

Patient navigation interventions (PNIs) can provide personalized support and promote appropriate coordination or continuation of health and social care services. Online PNIs have demonstrated excellent potential for improving patient knowledge, transition readiness, self-efficacy, and use of services. However, the characteristics (ie, intervention type, mode of delivery, duration, frequency, outcomes and outcome measures, underlying theories or mechanisms of change of the intervention, and impact) of existing online PNIs to support the health and social needs of individuals with illness remain unclear.

**Objective:**

This scoping review of the existing literature aims to identify the characteristics of existing online PNIs reported in the literature.

**Methods:**

A scoping review based on the guidelines outlined in the Joanna Briggs Institute framework was conducted. A search for peer-reviewed literature published between 1989 and 2022 on online PNIs was conducted using MEDLINE, CINAHL, Embase, PsycInfo, and Cochrane Library databases. Two independent reviewers conducted 2 levels of screening. Data abstraction was conducted to outline key study characteristics (eg, study design, population, and intervention characteristics). The data were analyzed using descriptive statistics and qualitative content analysis.

**Results:**

A total of 100 studies met the inclusion criteria. Our findings indicate that a variety of study designs are used to describe and evaluate online PNIs, with literature being published between 2003 and 2022 in Western countries. Of these studies, 39 (39%) studies were randomized controlled trials. In addition, we noticed an increase in reported online PNIs since 2019. The majority of studies involved White females with a diagnosis of cancer and a lack of participants aged 70 years or older was observed. Most online PNIs provide support through navigation, self-management and lifestyle changes, counseling, coaching, education, or a combination of support. Variation was noted in terms of mode of delivery, duration, and frequency. Only a small number of studies described theoretical frameworks or change mechanisms to guide intervention.

**Conclusions:**

To our knowledge, this is the first review to comprehensively synthesize the existing literature on online PNIs, by focusing on the characteristics of interventions and studies in this area. Inconsistency in reporting the country of publication, population characteristics, duration and frequency of interventions, and a lack of the use of underlying theories and working mechanisms to inform intervention development, provide guidance for the reporting of future online PNIs.

## Introduction

### Background

Individuals living with chronic illness or illnesses or disability have reported increased reliance on the health care system, as well as social supports for relevant resources or services (ie, medication, equipment, therapy, and counseling), particularly emphasizing the past decade [1-4]. This poses a problem, as they also face a number of challenges when navigating the health care system. These challenges can be attributed to various factors, such as a lack of proper care coordination and continuity of health care services [5-8]. Other concerns include patients’ inadequate knowledge related to their conditions or disabilities and the lack of adherence to treatment plans [2,9]. It is also specifically challenging for patients with complex health needs to find appropriate health care services as there is a lack of training in specialized care provision [10-12]. Altogether, these challenges pose a threat to the use, coordination, and continuation of health care services for patients with chronic health conditions or disabilities.

In particular, individuals struggle with coordination difficulties [13]. Literature supports this finding, with a relationship between self-reported care coordination difficulties and the level of patient engagement and chronic illness complexity being observed [13,14]. It is critical to address this gap by providing navigation services for these patients with multiple and complex chronic conditions as a lack of proper coordination and continuation of services can lead to negative outcomes related to one’s health and well-being, including one’s ability to integrate and participate within the community [5-8,13,15].

Patient navigation commonly involves the use of one-to-one interactions between navigators and patients or their family members and caregivers to promote recommended health care use behaviors from patients’ screening, to diagnosis, to resolution [10,16-19]. Patient navigation can be provided in the form of a professional, lay, or peer (with training) navigator [20].

Current literature has identified patient navigation interventions (PNIs) as an effective care approach for populations with chronic illness or disabilities in relation to managing their care through assistance with navigating the health care system [1,10,13,16,21-27]. In a systematic review, McBrien et al [27] assessed the impact of patient navigation on patients living with chronic diseases such as cancer, diabetes, HIV or AIDS, cardiovascular disease, chronic kidney disease, and dementia. The authors found that of the included 67 randomized controlled trials (RCTs), 44 trials indicated that patient navigation improved primary outcomes, specifically those related to the patients’ care or health care navigation process [27]. A meta-analysis of RCTs involving various patient populations revealed that compared to usual care, patient navigation more than doubled the likelihood of patients’ health screening rates and attendance at care events [25]. Similar findings were reported in a scoping review by Kokorelias et al [10] that summarized the literature on patient navigation for adults with chronic conditions, whereby patient navigation increased a patient’s overall satisfaction with their care and improved access to care, education, and adherence to medication and treatment completion. Likewise, in the context of cancer care, reviews of patient navigation have concluded that PNI programs were found to be cost-effective approaches to care when considering factors such as life expectancy, incremental cost-effectiveness ratios, and quality-adjusted life-years [28,29], thus further supporting the benefit and need for patient navigation. While informative, these reviews focused on PNIs in general and were not specific to online PNIs.

One example of patient navigation is peer navigation, which involves trained peer navigators who have lived experiences of health conditions or disabilities that they can use to provide personalized support to patients with different needs [19,23,30-33]. Personalized support in patient or peer navigation may involve the following types of support: educational or informational (sharing of advice, personal experiences, first-hand knowledge, resources, and factual information), psychosocial (provision of emotional and social support using empathy, validation, mentorship, motivation, feedback, and reflection), and instrumental (assistance with administrative activities, accessing and navigating services or resources, advocacy) [19,23,30-33].

Consistent with the theoretical underpinnings of the Social Cognitive Theory [34], the provision of such personalized support in patient navigation can promote patients’ perceived self-efficacy, appropriate health care use behaviors, and related outcomes (ie, community integration, quality of life, and well-being). For instance, Cabassa et al [35] systematic review identified peer-based navigation interventions to be among the most promising interventions for improving the health outcomes of individuals with serious mental illnesses. Peer navigators with lived experience improved health outcomes by facilitating linkages between individuals seeking care and health care services [10,36-39].

One area of development that warrants further exploration is online PNIs for a breadth of chronic conditions in the adult population. Research has shown that online-based PNIs have a great potential for improved health outcomes (eg, increased patient knowledge, transition readiness, self-efficacy, and appropriate use of health care services) in various patient populations. Casillas et al [40] conducted a three-arm RCT to test the efficacy of both a peer navigation intervention and an intervention involving the use of mobile technology (ie, SMS text messaging) in promoting cancer survivorship care in adolescents and young adults. Compared to standard care, these online interventions demonstrated the following statistically significant benefits: online peer navigation improved participants’ self-efficacy in survivorship care, SMS text messaging improved survivorship-focused knowledge, and both interventions improved participants’ attitudes in seeking survivorship care [40]. Specifically, the SMS text messaging group exhibited higher levels of survivorship care knowledge compared to the control group (*P*<.05), while the peer navigation group showed increased survivorship care self-efficacy compared to the control group (*P*<.05). Both intervention groups demonstrated more positive attitudes toward seeking survivor-focused care compared to the control group (SMS text messaging group: *P*<.05; peer navigation group: *P*<.05) [40]. Considering the initial efficacy observed in both interventions, each has the potential to be used in the future to educate and empower adolescent and young adult cancer survivors in accessing necessary survivorship care [40]. Online support has also been deemed a more flexible and sustainable care model when offered to individuals with intellectual disabilities, especially during the COVID-19 pandemic [41]. Moreover, online patient navigation can better reach rural, remote, and other underserved communities.

### Objective

Despite the demonstrated benefits of online patient navigation for various patient populations, the extent of the literature specifically focused on online PNIs across a range of chronic conditions or disabilities is uncharted. Therefore, the purpose of this scoping review is to comprehensively search databases and summarize data from peer-reviewed publications to address the following research question: What is known from the existing literature about the key characteristics (ie, intervention type, mode of delivery, duration, frequency, outcomes and outcome measures, underlying theories or mechanisms of change of the intervention, and impact) of online PNIs used across a range of chronic conditions or disabilities?

## Methods

### Research Design and Methodological Framework

A scoping review methodology was used given the broad nature of the research objective and question, and the lack of previous comprehensive reviews conducted in this area. A scoping review, also known as a scoping study, serves as a form of knowledge synthesis designed to explore research questions and map key concepts, types of evidence, and research gaps related to a defined area or field. This approach involves systematic searching, selection, and synthesis of existing knowledge [42] (page 28). Thus, a scoping review was deemed suitable to help identify key concepts and evidence related to online PNIs for adults with chronic conditions or disabilities. This scoping review was guided by the framework proposed by the Joanna Briggs Institute (JBI) Manual for Evidence [43-45]. The JBI framework was selected as it was developed based on previously reported methodological frameworks by Arksey and O’Malley [46] and Levac et al [47]. This refined framework provides additional guidance and clarity on the steps involved in the collection, analysis, and dissemination of research findings [43-45]. Specifically, the JBI framework focuses on aspects of the research process that have not been addressed as extensively in previous frameworks. The methods and the findings are reported according to the PRISMA-ScR (Preferred Reporting Items for Systematic Reviews and Meta-Analyses extension for Scoping Reviews; Multimedia Appendix 1) to further enhance the reporting of findings, as consistent with the JBI methodology [43-45,48]. A protocol was not published prior to the completion of this scoping review.

### Inclusion and Exclusion Criteria

All empirical study designs (eg, experimental, quasi-experimental, observational, qualitative studies, not review methodologies) reported in peer-reviewed, full text (eg, no conference abstracts) were included to increase the scope of the literature found. To address the identified gaps in the current literature on online PNIs, only peer-reviewed studies involving trained navigators and PNIs delivered using an online format, and software or application-based PNIs both with or without allocated trained navigator support were included. Our review encompasses a broad spectrum of online PNIs including those featuring hybrid formats. These interventions incorporate both online elements and face-to-face or other non–internet-related components, ensuring a comprehensive assessment of diverse intervention modalities and their characteristics. Participants in these studies had to be adults (aged 18 years and older) with chronic conditions or disabilities as recognized by the Public Health Agency of Canada (PHAC) [49], the Canadian Chronic Disease Surveillance System, and the World Health Organization. These conditions based on their PHAC categorization could include exclusively physical or mental health–based conditions, or both. Examples of common chronic diseases and conditions, as defined by the PHAC, the Canadian Chronic Disease Surveillance System, and the World Health Organization, include cardiovascular disease (eg, heart failure, hypertension, and stroke), chronic respiratory disease (eg, asthma and chronic obstructive pulmonary disease), diabetes mellitus (types combined, but not gestational diabetes), mental illnesses (alcohol or drug-induced disorders, mood and anxiety disorders, and schizophrenia), musculoskeletal disorders (eg, arthritis and osteoporosis), and neurological conditions (eg, dementia, epilepsy, multiple sclerosis, and Parkinson) [49-51]. Additionally, studies with participants living with HIV and AIDS were also included [52]. Only studies published between 1989 and 2022 and available in full text in English were included due to feasibility considerations (ie, members of the research team could only read in English) and resource constraints. The Report to the Nation on Cancer in the Poor that began work in the area of patient navigation began in 1989 [21]. Exclusion criteria included PNIs that were delivered in alternate formats (eg, face-to-face, telephone, and mail).

### Data Collection and Management

Comprehensive literature search strategies based on the inclusion and exclusion criteria of this scoping review were developed in collaboration with an experienced librarian (LP). The search strategies were further informed by the Participants/Concept/Context framework as recommended in the JBI methodology. The search strategy included medical subject headings and text words related to adults with chronic conditions or disabilities and online PNIs (Multimedia Appendix 2). The search strategy was first developed, tested, and refined in MEDLINE (OVID interface) prior to being used in other databases. The following databases were searched using the finalized MEDLINE strategy: CINAHL (EBSCO interface), Embase (OVID interface), PsycInfo (OVID interface), and Cochrane Central Register Controlled Trials (Cochrane Library). The use of multiple health care–related databases helped broaden the scope of the comprehensive literature search. Data yielded from the comprehensive literature search strategies were stored and screened using the online Covidence software program (SaaS Enterprise) [53,54]. These data were screened at 2 levels (ie, level 1 and level 2 screening). Study titles and abstracts were screened first, followed by the screening of full-text studies. Screening at both levels was conducted by 2 independent reviewers (MM and SRS) to ensure accuracy in the included results. Discrepancies were addressed through consensus between the reviewers and the senior author (KMK). Reference lists of all included studies were reviewed to determine any studies that may have been missed from the database search. Gray literature was not included.

### Data Extraction and Analysis

Data extraction was carried out by extracting key information or data from the included studies. A data extraction form, developed by the authors, was used to chart and record this information to ensure easy referencing and tracking of each study to ensure clarity. The form was first piloted on the first 5 included studies by all members of the research team. The extraction template was further informed by the Template for Intervention Description and Replication (TIDieR) checklist and guide, which is a framework that aims to promote replicability and implementation of interventions through the consistent reporting of key intervention characteristics [55]. The following data were extracted from the full-text studies: study characteristics (ie, title, author or authors, publication year, publication country, study purpose or objective or objectives, and study design), participant population characteristics (ie, sample size, race or ethnicity, condition or disability, age, and sex), and key characteristics of the intervention (ie, name, type, description, setting, duration, frequency, mode of delivery, underlying theories, behavior change techniques or working mechanisms, context, outcome measures used, and quantitative and qualitative outcomes). In line with scoping review methodologies, we did not evaluate the quality of included studies [56]. Data were extracted by 2 independent reviewers (MM and SRS) and any disagreements were resolved through consensus. Following data extraction, the following information was specifically summarized using descriptive statistics [57] and directed content analysis [58] to provide an accurate overview of the published literature on the key characteristics of online PNIs in adults with chronic conditions or disabilities. The research team reviewed the coded data to create a set of categories that capture the key themes, concepts, and variables relevant to the research question. This involved both inductive categories (emerging from the data) and deductive categories (informed by the TIDieR framework). The authors then began coding the selected studies according to this scheme, using Excel (Microsoft Corp) to facilitate this process. The Excel document was then reviewed by all members of the research team to identify patterns and trends. The team met over a series of meetings to determine key interpretations of the results.

## Results

### Overview

**Figure 1:**
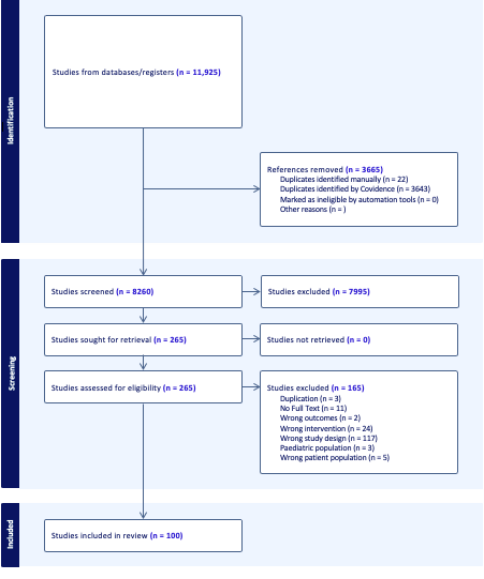
PRISMA-ScR (Preferred Reporting Items for Systematic Reviews and Meta-Analyses extension for Scoping Reviews) flowchart.

The PRISMA-ScR flowchart displayed in Figure 1 shows an overview of our comprehensive literature search, which yielded 11,925 studies.

### Study Characteristics

The publication dates ranged from 2003 to 2022, with some years yielding no publications. The greatest number (n=20, 20%) were published in 2019 with 19 (19%) studies published in 2020. As for the publication country, all studies were published in Western countries. A total of 48 (48%) studies were conducted and published in the United States, followed by 23 (23%) studies from Canada, and 19 (19%) studies from the United Kingdom. There was a variation of study designs observed in the review. The most common study design included RCTs (n=39, 39%). The included studies focused on the development, implementation, and testing of online PNIs. Of the studies included, 82 (82%) studies specified their sample size. There was a significant range in the overall participant sample size (including intervention and control arm or arms), observed from 9 to 14,584 participants, with the median being 97 (IQR 342) participants. When comparing the sample sizes of the intervention and control arms, 24 studies had an approximately equal division, while 21 studies did not. In addition, 55 studies did not specify the sample size of the intervention or control groups. Multimedia Appendix 3 outlines the study characteristics.

### Population Characteristics

Among the included full-text studies, the majority (n=79, 79%) of the studies’ participants had conditions or disabilities that would be classified exclusively as physical health-based conditions according to the PHAC (including cancer, heart disease, diabetes, and stroke), while only 11% (n=11) of the studies represented participants with solely mental health-based conditions (eg, mood disorders, substance use and addictions, and eating disorders). A total of 10% (n=10) of the studies represented participants who had both physical and mental health–based conditions. One study represented participants who had post–COVID-19 condition. In terms of the participants’ age, 55% (n=55) of the studies provided this information. Across the studies that reported age (regardless of study design), the mean age range (with SD) of the participants included in the studies’ intervention arms (ie, those who participated in online PNI) was between 19.84 (SD 1.61) and 69.6 (SD 9.1) years. When comparing the age of the participants in the intervention and control arms, 37% (n=37) of the studies had similarly aged participants (ie, 20-70 years of age). With regards to the sex of participants, 20 (20%) studies included only female participants while only 4 (4%) studies included only male participants. A total of 19 (19%) studies had an approximately equal distribution of females and males, and 14 (14%) studies did not specify the sex of the participants or inconsistently reported this information. In terms of the racial or ethnic representation of participants in studies, more than half (n=54, 54%) of the studies did not specify this information. Among those that did, in 20 (20%) studies, the majority of the participants were White, followed by the following racial groups: Black, African, or African-American (16 studies), Hispanic or Latinx (5 studies), and Asian or Asian American (4 studies; terminology as used by the original authors of the included studies)*.* Only one study focused on the Indigenous population (in Australia).

### Intervention Characteristics

#### Types of Intervention

Among the 100 included studies, the most common intervention type was a combination (n=61, 61%), which included a mix of peer, patient, and other navigation types including coaching, digital navigation (including mobile, eHealth, or telehealth-based interventions, or software application-based interventions), and self-management. Within the “other” category, intervention types included a mixture or combination of peer, patient, and other navigation intervention types, as well as the exclusive implementation of interventions such as coaching, counseling, health promotion, digital navigation, self-monitoring, or self-management. A total of 32 (32%) studies were exclusively patient navigation-based, and 7 (7%) studies were exclusively peer navigation-based. Other studies did not clearly specify the intervention type (ie, who the intervention was led by or what it involved).

Several studies explored diverse online PNIs for cancer-related support and care. While the interventions varied across studies, commonalities and differences emerged. One study introduced a virtual navigation tool providing comprehensive cancer information accessible 24/7, emphasizing its value in validating information and controlling exposure, while another used nurse navigators sending scripted messages, resulting in improved quality of life and symptom burden among participants [59]. A similar modality was used by other studies that used peer navigation and SMS text messaging interventions for educating cancer survivors on late effects and survivorship care, leading to improved survivorship knowledge, attitudes, and self-efficacy [40]. On the contrary, some scholars focused on self-monitoring and physical activity, noting enthusiasm and continued use among participants [60,61].

In the context of multimorbidity, the studies featured various adaptations and design elements tailored for both physical and mental health conditions and examined technology use for patients with multiple chronic conditions, focusing on communication tools, tracking medical information, and decision-making support, intending to address self-management challenges and health care navigation issues [62]. Allen et al [63] developed an internet-based health coaching intervention targeting chronic pain, depression, and mobility difficulties, emphasizing patient-clinician communication improvement and patient empowerment through goal-setting and constructive communication tools. On the other hand, some interventions focused on peer visitation, support groups, and educational materials, enhancing recovery expectations and satisfaction [64,65]. Thus, both chronic conditions, like cancer and multiple chronic conditions, used strategies such as technology integration for communication, support, and information dissemination, tailored interventions addressing specific conditions and associated challenges, peer support networks fostering engagement and optimism, and empowerment strategies encouraging collaborative patient-clinician communication, goal-setting, and self-efficacy. Multimedia Appendix 4 outlines the intervention characteristics.

#### Duration and Frequency

Of the included studies, the duration and frequency of interventions were varied. Of the 100 included studies, 55 (55%) studies specified a duration and only 17 (17%) studies specified a frequency of the intervention. Other studies included a variable duration or frequency that was tailored to the needs of individual patients. Of the 55 (55%) studies that specified duration, 7 (7%) studies were offered for a year. A total of 41 (41%) studies were offered for 1 month up to 11 months, 3 studies were offered weekly (eg, one time per week), and 4 (4%) studies were held on a daily basis. In addition, of the total studies included, 14 studies had a variable duration, 24 (24%) studies did not specify, and there were 7 (7%) studies where the duration was not applicable (eg, proof of concept, usability, and beta-testing). In terms of frequency, of the 17 (17%) studies that specified frequency for interventions, 3 (3%) studies were month-based, 10 (10%) studies were week-based, and 4 studies required day-to-day engagement from participants. Of the total studies included, 46 (46%) online PNIs had variable frequency, 30 (30%) did not specify frequency, and there were 7 (7%) studies that reported interventions where the frequency was not applicable as the interventions only occurred once.

#### Mode of Delivery

Of the included studies, 56 (56%) of the 100 studies reported using an online mode of asynchronous or synchronous delivery for interventions, without any other components. A total of 13 (13%) studies used a format that was hybrid, with both online and offline intervention formats. In total, 22 (22%) studies included SMS text messaging as the main component of intervention delivery, of which 14 (14%) studies had SMS text messaging mixed with other intervention formats such as the use of telephone calls, educational videos, websites, and online support groups. Finally, 9 (9%) of the 100 studies used a mixed format including intervention components such as software programs and applications, telephone calls, in-person interactions, email, automated phone lines, and other online-based intervention formats.

#### Underlying Theories

In total, 78 (78%) studies did not specify the use of any underlying theories, models, and frameworks, that were used to guide the PNIs. Of the 22 (22%) studies that did specify an existing theory, a total of 21 different theories were identified. Four studies indicated that the intervention was based on more than one theory. Social cognitive theory followed by the self-determination theory, self-efficacy theory, behavior change theory, and community empowerment were the most common theories used. The most common working mechanism among these was a combination (n=20, 20%) of various mechanisms, which included coaching, education, peer support, navigation, self-management, and cognitive behavioral therapy among others. Self-management (n=19, 19%) and navigation (n=19, 19%) followed as other commonly identified mechanisms.

#### Outcome Measures

Multiple outcome measures were used in these studies; however, the most commonly used outcome measures included: the Short Form-36 survey questionnaire to measure participants’ health-related quality of life, the Patient Health Questionnaire-9 to measure participants’ psychological outcomes, and the Health Education Impact Questionnaire to measure participants’ knowledge and self-management related outcomes. Additional standardized and nonstandardized outcome measures were used to report on common intervention outcomes such as the interventions’ feasibility, acceptability, efficacy, effectiveness, uptake, use, and retention, and participants’ clinical symptoms or outcomes (physical, mental, emotional or psychosocial), lifestyle or behavioral changes, quality of life, user experience or satisfaction, adherence, knowledge, attitudes, and self-efficacy.

#### Outcomes and Impact of Online PNIs

Out of the 76 (76%) studies that reported quantitative findings (including RCTs and non-RCTs), 46 (60.5%) studies demonstrated significant improvements. Improvements were commonly demonstrated in the following outcomes: appointment adherence, intervention retention, knowledge, self-monitoring of symptoms, and physical and mental health symptoms. Of the 23 (23%) studies that reported qualitative findings, 8 (35%) studies identified specific themes and subthemes [62,66-72]. The included qualitative studies spoke of themes that described usability or user experience, as well as participants’ experience with the PNI as it related to self-management, education, knowledge, navigation, engagement, encouragement or support, and feedback.

## Discussion

### Principal Findings

This scoping review aimed to investigate the key characteristics of reported online PNIs to inform future intervention development and research evaluation. A total of 100 peer-reviewed studies were included. Overall, online PNIs are highly variable with various modalities for delivery, durations, frequencies, and contexts of support provided. Few studies reported participants’ sex, diagnoses, age, and race or ethnicity. Moreover, most of the literature was published in Western countries, resulting in a lack of data from non-Western countries, as well as PNIs that reflect the needs of individuals from non-Western countries. Despite this, we were able to ascertain through the results of 20 RCTs (the highest level of evidence) [73] that in general, online PNIs improve outcomes of patients’ self-management, knowledge, clinical symptoms (physical or mental health–based), and use and navigation of health care services.

The majority of the online PNIs were designed for physical health-based conditions (including cancer, heart disease, diabetes, and stroke), while few studies focused solely on mental health–based conditions. Our investigation revealed a notable scarcity of online PNIs specifically targeting multimorbidity of physical and mental health conditions (n=10), signifying a considerable gap in available interventions addressing the complex needs of individuals with multiple chronic conditions. This paucity carries significant implications, indicating an unmet need within the digital health landscape, that is needed to ensure comprehensive care for those navigating multifaceted health challenges. Participants in the RCTs ranged from 20 to 70 years of age. As with other reviews of digital health interventions to support the coordination of care [74,75], our review noted a lack of inclusion of particular groups of older adults (ie, 70 years and older), despite this group representing a large proportion of individuals living with chronic conditions [76] who could benefit from online health interventions [75,77]. Moreover, our review found a lack of literature exploring the impact of online PNIs on Indigenous populations and non-White populations such as Black, Asian, and Hispanic individuals, making it difficult to ascertain their unique needs to inform further online PNIs. As such, future research on online PNIs is encouraged to explore the interaction of racial and cultural factors of different groups to improve service delivery [78].

Our review highlights how future online PNIs can better support various patient populations. Only one study cited in our review noted the racial preferences of participants in which Black patients preferred the services of a Black (virtual) provider [79]. Ethnic minorities and other underserved populations often face unique barriers to accessing health services that patient navigation is able to assist with overcoming [10,80,81]. Social and environmental factors, such as finances, health literacy, and availability of health services, influence health access [82-84]. To overcome these barriers, it is necessary to create efficient processes for referring communities affected by social and environmental factors to suitable resources, ensuring that their needs are adequately met [85,86]. Culturally appropriate patient navigation can assist with learning about the unique information needs and barriers that face particular communities and facilitate an appropriate referral and support process to services [10,87]. While online PNIs can help overcome traditional barriers to seeking support, such as transportation [88], it is important to consider that shifting to online PNIs may also increase risks to access and equity as a result of digital inequity (ie, gaps in use and participation in the use of technology) [89]. Future research efforts on online PNIs should also consider the individual needs of target populations (eg, access and geographical location, income, and digital literacy), as well as the significance of an individual-based versus group-based mode of delivery of online PNIs.

We also noted the lack of consistent reporting of intervention characteristics. For example, the duration and frequency of interventions were not reported consistently or were variable among the included studies in our review. Moreover, multiple studies did not specify the exact frequency of their intervention. Similar trends were observed in a previous review on web-based peer support interventions where the authors reported “a lack of consistency” and variation regarding the reporting of intervention characteristics such as duration and frequency [75]. The reporting of intervention doses associated with improved outcomes is important to guide other jurisdictions looking to implement or build upon existing interventions [90]. Frameworks, such as the TIDieR, have been posited as helpful for guiding researchers in reporting a full description of complex interventions [55] such as PNIs. The TIDieR can help guide the reporting of future online PNIs to ensure transparency and improve the quality of patient navigation research. Relying solely on reported intervention characteristics, however, can imply a limitation of the personalization of interventions (ie, inflexibility in the duration and frequency tailored to participant needs). While this can be a great guide for replicating the interventions, and further testing and implementation, patients with chronic illness may require individualized approaches to care [91]. Further research is needed to understand how the duration, frequency, and support provided within existing online PNIs may evolve across the illness and care trajectory of patients. Moreover, the TIDieR is only beneficial for reproducibility in the setting specified by the original individual study and therefore cannot guide researchers to implement the intervention in different contexts or settings [90]. Researchers should then reply on implementation frameworks, such as the PRACTical planning for Implementation and Scale-up guide to provide practical direction on implementing online PNIs into new sessions [92].

Despite a substantial portion (78%) of the studies not explicitly delineating underlying theories or frameworks, the 22% of studies that did highlight a diverse array of theoretical foundations (ie, Social Cognitive Theory emerged prominently, followed by self-determination theory, self-efficacy theory, behavior change theory, and community empowerment among others). This diversity underscores the need for a more comprehensive and structured integration of theoretical frameworks within the design and implementation of online PNIs. Integration of frameworks within online PNIs can help researchers understand the underlying mechanisms driving these interventions and will help to establish standardized evaluation metrics. Moving forward, comprehensive research could delve into exploring the efficacy and synergies of combining multiple theories to inform the design and implementation of online PNIs effectively. Furthermore, investigating how specific mechanisms within these theories (eg, coaching, education, and peer support) contribute to PNI outcomes can enrich our understanding and potentially optimize intervention strategies. This calls for a systematic and comparative analysis to discern the differential impact of diverse theoretical orientations on the effectiveness, sustainability, and scalability of PNIs across various health contexts and participant demographics.

Digital health interventions often incorporate elements akin to navigation programs including patient education, remote monitoring, and personalized feedback. These features aim to empower patients in self-management and facilitate communication with health care providers. In contrast, navigation programs traditionally focus on guiding patients through complex health care systems, providing support in appointment scheduling, access to resources, and continuity of care. However, as digital health evolves, distinctions between these approaches can blur. Many digital health solutions now integrate navigation functionalities such as decision support tools and care coordination platforms. This integration raises questions about the delineation between virtual care and navigation programs, particularly regarding their roles in improving health outcomes and patient experience across different chronic diseases. Moving forward, future research should explore synergies between digital health interventions and navigation programs to optimize their combined impact on chronic disease management. This includes examining the effectiveness of integrated approaches in enhancing patient adherence, reducing health care disparities, and improving the overall quality of care.

Finally, we found that online PNIs are an accessible and user-friendly option for navigational support to patients. Similar trends were observed in other studies involving peer and professional navigators [10,19,20,36,93-101]. Similarly, positive and statistically significant outcomes were also reported in another scoping review on web-based peer support, where the authors found that interventions in 4 of their 6 included RCTs improved the health navigation, emotional self-management, self-efficacy, social participation, and attitudes of adults with chronic conditions [75]. Overall, these findings demonstrate how online PNIs could play a crucial role in improving health use and navigation among adults with chronic conditions and disabilities. Additionally, a common theme that was reported by patients, specifically among the qualitative findings of studies from our review was that online PNIs provided more accessibility, engagement, and encouragement to participants navigating their health. Gaining insight on what would construe the ideal patient navigator and ideal patient navigator program for patients with chronic health conditions is still in its infancy [102,103], and as such conducting more qualitative research with diverse patient populations would be valuable in refining and co-designing novel online PNIs and navigator roles.

### Limitations

Although a systematic, comprehensive review was conducted to identify key characteristics of online PNIs, the authors acknowledge that this scoping review has some limitations. First, search results were limited to publications in English studies published after 1989, and while the broad search strategy made it unlikely that potentially eligible publications were missed as a result, we may have created a bias toward studies from English-speaking countries, which might have contributed to the majority of data coming from Western countries. We also excluded gray literature. The majority of the data extraction was not completed in duplicate, which may have affected the reliability of the extracted data. Incomplete reporting on study characteristics by original study authors also made it challenging to comment on additional participant characteristics (eg, socioeconomic status, education level, and digital literacy) that would have provided valuable information.

### Conclusions

This review has mapped the existing literature on online PNIs, and in doing so, has identified several gaps that should be addressed in future research and intervention development efforts. Although many positive outcomes were reported for online PNIs, a lack of variation in included study samples, as well as a lack of consistency in reporting, was observed in the reporting of TIDieR intervention characteristics including the following: the publication country of studies, population characteristics such participants’ age, sex, and racial or ethnic background, duration and frequency of interventions, and the use of underlying theories and working mechanisms to inform intervention development. Future research and development efforts should consider using theories and models, expanding inclusion criteria, and reporting key intervention characteristics more consistently.

## Appendix

Multimedia Appendix 1 PRISMA-ScR (Preferred Reporting Items for Systematic Reviews and Meta-Analyses extension for Scoping Reviews.

Multimedia Appendix 2 Search Strategy.

Multimedia Appendix 3 Overview of study characteristics.

Multimedia Appendix 4 Intervention characteristics.

## Abbreviations

JBI: Joanna Briggs Institute
PHAC: Public Health Agency of Canada
PNI: patient navigation intervention
PRISMA-ScR: Preferred Reporting Items for Systematic Reviews and Meta-Analyses extension for Scoping Reviews
RCT: randomized controlled trial
TIDieR: Template for Intervention Description and Replication
