# Supplementary material for: Characteristics of Existing Online Patient Navigation Interventions: Scoping Review
Source: JMIR Med Inform. 2024 Aug 19;12:e50307. doi: 10.2196/50307 (PMC11369544; doi:10.2196/50307)
Supplement: Multimedia Appendix 2 [file medinform_v12i1e50307_app2.doc]

Database: Ovid MEDLINE: Epub Ahead of Print, In-Process & Other Non-Indexed Citations, Ovid MEDLINE® Daily and Ovid MEDLINE® <1946-Present>

Search Strategy:

--------------------------------------------------------------------------------

1 Patient Navigation/

2 (care adj2 navigat$).tw.

3 (community-based adj2 navigation).tw.

4 ("community based" adj2 navigation).tw.

5 (coach$ adj2 coordinat$).tw.

6 (coach$ adj2 co-ordinat$).tw.

7 (coach$ adj2 navigat$).tw.

8 (guided adj2 care).tw.

9 (navigation adj2 lay$).tw.

10 (navigation adj2 nurs$).tw.

11 (navigation adj2 professional$).tw.

12 (navigation adj2 intervention$).tw.

13 (navigation adj2 clinic-based).tw.

14 (navigation adj2 "clinic based").tw.

15 (navigation adj2 telephone-based).tw.

16 (navigation adj2 "telephone based").tw.

17 (navigation adj2 program$).tw.

18 (navigation adj2 support$).tw.

19 (navigation adj2 role$).tw.

20 (navigation adj2 model$).tw.

21 (navigation adj2 approach$).tw.

22 (navigation adj2 partner$).tw.

23 (navigation adj2 impact).tw.

24 (navigation adj2 option$).tw.

25 (navigation adj2 format$).tw.

26 (navigat?r? adj2 lay).tw.

27 (navigat?r? adj2 personal).tw.

28 (navigat?r? adj2 peer).tw.

29 (navigat?r? adj2 nurs$).tw.

30 (navigat?r? adj2 professional$).tw.

31 (navigat?r? adj2 intervention$).tw.

32 (navigat?r? adj2 clinic-based).tw.

33 (navigat?r? adj2 "clinic based").tw.

34 (navigat?r? adj2 telephone-based).tw.

35 (navigat?r? adj2 "telephone based").tw.

36 (navigat?r? adj2 program$).tw.

37 (navigat?r? adj2 support$).tw.

38 (navigat?r? adj2 community-based).tw.

39 (navigat?r? adj2 "community based").tw.

40 (navigat?r? adj2 role$).tw.

41 (navigat?r? adj2 model$).tw.

42 (navigat?r? adj2 approach$).tw.

43 (navigat?r? adj2 partner$).tw.

44 (navigat?r? adj2 impact).tw.

45 (navigat?r? adj2 option$).tw.

46 (navigat?r? adj2 format$).tw.

47 (patient$ adj2 coach$).tw.

48 (patient$ adj2 navigat$).tw.

49 (personal adj2 navigation).tw.

50 (peer adj2 navigation).tw.

51 (service$ adj2 coach$).tw.

52 (service$ adj2 navigat$).tw.

53 (system$ adj2 coach$).tw.

54 (system$ adj2 navigat$).tw.

55 or/1-54

56 Computer-Assisted Instruction/

57 Computers/

58 exp Cell Phones/

59 Electronic Mail/

60 Educational Technology/

61 exp Internet/

62 Mobile Applications/

63 exp Microcomputers/

64 exp Software/

65 User-Computer Interface/

66 exp Videoconferencing/

67 Web Browser/

68 Webcasts/

69 (cell adj phone$).tw.

70 chatroom$.tw.

71 (chat adj room$).tw.

72 computer$.tw.

73 (computer adj based).tw.

74 (computer adj mediated).tw.

75 (computer adj assisted).tw.

76 (computer adj supported).tw.

77 email$.tw.

78 e-mail$.tw.

79 ebased.tw.

80 e-based.tw.

81 e-health.tw.

82 ehealth.tw.

83 (electronic adj health).tw.

84 (hand adj held).tw.

85 Internet.tw.

86 (information adj technolog$).tw.

87 (mobile adj app$).tw.

88 (mobile adj phone$).tw.

89 online.tw.

90 on-line.tw.

91 "personal digital assistant?".tw.

92 smartphone$.tw.

93 smart-phone$.tw.

94 website$.tw.

95 web-site$.tw.

96 (web adj based).tw.

97 virtual.tw.

98 or/56-97

99 55 and 98

100 exp Animals/ not (exp Animals/ and Humans/)

101 99 not 100
