# Supplementary material for: Characteristics of Existing Online Patient Navigation Interventions: Scoping Review
Source: JMIR Med Inform. 2024 Aug 19;12:e50307. doi: 10.2196/50307 (PMC11369544; doi:10.2196/50307)
Supplement: Multimedia Appendix 3 [file medinform_v12i1e50307_app3.docx]

| **Author, Year, Country** | **Objectives** | **Study Design** | **Sample Size (Overall)** | **Sample Size (Overall); Intervention, Control** | **Participant Condition** | **Participant Ages** | **Participant Race** | **Study Results** |
| --- | --- | --- | --- | --- | --- | --- | --- | --- |
| Abbey B. Berenson  et al., 2016  United States | At the University of Texas Medical Branch (UTMB), we surveyed 500 patients attending five prenatal clinics in 2012 to determine whether a postpartum vaccination program would be acceptable among women residing in southeast Texas.12 Over 80% said they were willing to receive a free HPV shot in the hospital after childbirth. Based on these findings, UTMB obtained funding to begin a prevention program that offered counseling for pregnant and postpartum women about HPV and the HPV vaccine. Here, we report on the success of this program during the first 20 months of its implementation. | Non-randomized experimental study | Not specified | Intervention = 575  Control = Not specified | Postpartum women receiving human papillomavirus (HPV) vaccination program (3 dose series) - physical | Age range provided only: 14-20 years - n=130 (22.6%)  21-26 years - n=445 (77.4%) | Of intervention group: Non-Hispanic White = n: 141 (24.5%)  Non-Hispanic Black = 120 (20.9)  Hispanic = 307 (53.4)  Non-Hispanic Others = 7 (1.2) | Among those screened on the postpartum unit, only 25.4% had previously received any HPV vaccines. At the time we conducted this assessment, 80.8% of patients had received at least 1 vaccine, and completion rates rose from 15.5% at baseline to 65.1% as a result of this program. Among new initiators who completed the series (n=436), 265 (60.8%) patients completed the series within 6 months as recommended by the CDC. The average time between the first and second dose was 2.3±2.1 months and the average time between the second and third dose was 5.6±2.5 months. A total of 1,651 vaccine doses were provided for the 575 new initiators and 103 incompletely vaccinated patients who participated in this program. Almost 59% of doses were funded by Medicaid, 36% by CPRIT, and 5% by private insurance. CPRIT funded 2%, 42% and 78% of the first, second, and third doses, respectively. A higher proportion of those that did not miss follow-up appointments completed the vaccine series compared to those that missed at least 1 appointment (87.9% vs. 70.2%, p<0.001). Among 575 postpartum HPV vaccine initiators, 182 (31.7%) attended all appointments while 393 (68.3%) missed at least one follow-up appointment. Further analysis of missed appointment data showed that non-Hispanic blacks and patients with two or more prior pregnancies were more likely to miss one or more follow-up appointments (Table 3). Women ≥21 years of age and those who received automated text messages were less likely to miss an appointment. Automated phone calls and text messages were received by 87.0% and 78.8% of patients, respectively, prior to any appointments. The median number of times PNs called a patient due to missed appointments was 4 (interquartile range 1–8). Overall, 12.7% of women in the project received letters and 6.1% received emails because they missed appointments and could not be reached by phone. Of the 72 patients that initiated the HPV vaccine postpartum and were lost to follow-up, 34 patients moved out of Galveston County (5.9% of vaccine initiators), 29 patients (5.0%) changed their telephone number or address and could not be found, and 9 (1.6%) were lost to follow-up for other reasons. From ABSTRACT: Of 1,038 patients approached, only 161 (15.5%) had previously completed the vaccine series. Of the 877 patients who had not completed the series, 661 (75.4%) received at least one dose postpartum, with 575 patients receiving their first dose and 86 receiving their second or third doses. By April 2015, initiation rates had increased as a result of this program from 25.4% before the program was initiated to 80.8% and completion rates from 15.5% to 65.1%. Missed appointments for injections were less likely among those who received text message reminders and more likely among those with ≥2 prior pregnancies. Those who were Hispanic or had received an influenza vaccination in the last year were more likely to initiate and complete the series through this program. Patients who missed 1 or more follow-up appointments were less likely to complete the vaccine series. |
| Ahmed Fadhil et al., 2019  United States | To assess the acceptability of this form of coaching by the users, and the feasibility of delivering behavior counseling using the conver- sational agent medium. Specifically, we are interested in assessing user trust and general satisfaction with the virtual agent to guide our next steps in development and help form hypotheses for future research. Our research work aims at answering the following research questions: RQ1: What is the overall user experience of using a CoachAI system? RQ2: Will user experience change over time when using CoachAI over 4 weeks? RQ3: How much do users prefer a direct human agent support or to what extent does the conversational agent satisfy their needs? | Other: Validation study | 19 | Intervention = 19 | All subjects were healthy, with no health condition and were motivated to participate in the experiment and perform health promotion activities. | Aged: 19–53, std. deviation 1⁄4 9.371 | Not specified but participants were either native Italian speakers or spoke Italian fluently | The system calculated participants’ overall adherence to the plan and reported their total adherence at the end of each plan expiration. The adherence was categorized into high and low adherence groups. Users performing above the threshold were categorized as highly adherent, and vice versa. Participants were categorized according to their adherence to the plan into high and low adherence, with respectively 10 and 9 participants each. The TAM dimensions were analyzed with a multivariate ANOVA (MANOVA) with “adherence level” as between subject factor. The MANOVA shows a significant effect of the between subject factor for usefulness (F(1,16) 1⁄4 6.5, p < .01), fun (F(1,16) 1⁄4 4.5, p < .01), and attitude (F(1,16) 1⁄4 6.9, p < .01). No differences were found for the ease of use and intention. Participants highly rated both attitude and intention toward using the system. Most participants gave a rating above average to both dimensions. Considering the questionnaire on the dimensions of TAM (►Fig. 7), a one-sample t-test was used to compare the means for each scale to the scale middle value (score 1⁄4 4). The scales “ease of use,” “attitude,” and “intention” are significantly higher than the middle score (respectively: t(17) 1⁄4 4.9, p < .01; t(17) 1⁄4 2.5, p < .05; t (17) 1⁄4 3.1, p < .01). A repeated measure ANOVA (with time as a within factor) showed a difference only for the scale “intention” between the three different weeks. Participants’ intention to change risky behavior or adopt a new measure was evaluated through the motivation and volition phases of the HAPA model.41,47 The HAPA stage model was adopted to measure participants’ intention to change or improve self-efficacy, outcome expectancy, and risk perception. Users in this model can either progress their behavioral intention to change or they may also fall into relapses and recycle through the stages. We compared these stages with individual’s overall adherence to the activities. Participants were asked to provide their answers to the HAPA questionnaire over the month. We analyzed individual’s data about physical activity, healthy diet, and overall health expectation and mapped these data with the perceived self-efficacy, outcome expectancy, and risk perception. |
| Aliza Selter Christina et al., 2018  Canada | This study was performed to (1) describe patient engagement with the Limbr program, (2) describe the patient-perceived utility of the Limbr program, and (3) assess the validity of the Your Activities of Daily Living module as a quantifier of pain and disability level among patients with CLBP. | Pilot study | 35 | Intervention = 35 | Chronic low back pain or discogenic back pain - physical health | Mean (SD): 46 (16) years | Not specified | A total of 93 participants were enrolled; of these, 35 (38%) completed the program (age: mean 46, SD 16 years; female: 22/35, 63%). More than half of completers finished assessments at least every 3 days and 70% (19/27) used the rehabilitation component at least once a week. Among respondents to a Web-based feedback survey, 76% (16/21) found the daily notifications helped them remember to complete their exercises, 81% (17/21) found the system easy to use, and 62% (13/21) rated their overall experience good or excellent. Baseline Your Activities of Daily Living score was a significant predictor of baseline ODI score, with ODI increasing by 0.30 units for every 1-unit increase in Your Activities of Daily Living (P<.001). Similarly, hierarchical linear modeling analysis indicated that Your Activities of Daily Living daily assessment scores were significant predictors of ODI scores over the course of the study (P=.01). |
| Amanda Gehrke et al., 2018  United States | The present study examined BCS and nurse case managers’ views about the components of a new IT approach to potentially compliment SCPs: the Cancer Survivor Profile- Breast Cancer (CSPro-BC) app. | Phenomenological study | 11 | Intervention = 11 | Physical health - Breast cancer (survivors) | 35-44 27.3%, 45-54 36.4%, 55-64 27.3%, 65-74 9.1% | Caucasian 72.7%, Black 9.1%, Asian 9.1%, Declined to respond 9.1% | From abstract: BCS (N = 11) were middle-aged and a median of 2.4 months post active treatment. Likert ratings indicated that the majority of BCS (91%) found the graphic profile represented each of the 18 prob- lem areas in an understandable manner, and all BCS thought that it was a useful communication tool to discuss problem areas with their nurse. No BCS endorsed the statement that it Bseemed like a waste of time—I knew this already.^ Ratings indicated that 82% of BCS thought the graph could Bstand on its own with little explanation.^ It was also observed that the majority of scores (bars on the graph) fell into the yellow or Bwatch^ category. Patients often wanted to discuss these yellow areas with the nurse navigator and requested additional resources for these areas, as well. While most BCS (89–91%) thought the problem-specific resources would be helpful, some were not certain.  From abstract: Structured questionnaires indicated the following: survey covered meaningful problem areas, profile display was clear, and nurse’s involvement was helpful. Follow- up interviews (2 weeks later) revealed that BCS shared their profile with others, but most BCS did not use the resources and those who did thought there were too many. |
| Anetta Hinchliffe & W. Kerry Mummery, 2008  Australia | The purpose of this study was to employ usability testing of an existing health promotion website (www.10000steps.org.au) to inform modifications to the website and to identify common usability themes in the redevelopment of this site that may serve to guide future website development and maintenance in the field of health promotion. | Other: This study conducted usability testing of an existing health promotion website to inform modifications and to identify common usability themes that should be addressed by organisations developing or maintaining a health promotion website | A purposeful sample of 12 users was involved in the usability testing sessions. Six users were recruited at the time of each testing session. | A purposeful sample of 12 users was involved in the usability testing sessions. Six users were recruited at the time of each testing session. | Not specified - website for physical activity health promotion | Female: 6 years±4.4, Male: age 47 years±4.1 | Not specified | Comparing the problem counts of UT1 and UT2 showed a significant decrease in the number of unique problems (t12=2.95, p=0.004) and the problems-per-user (t12=2.54, p=0.03). Table 1 provides an overview of the problems found in both usability testing sessions  The mean time taken to complete the 14 tasks in UT1 was 21.59 minutes (SD±4.8) and in UT2 was 10.18 minutes (SD±6.2). Overall, the modifications to the website resulted in the mean time to complete the tasks decreasing by 52% (9.05 minutes). An independent sample t-test revealed this as a significant decrease in time (t10=3.56, p=0.005) Internal consistency (Cronbach’s alpha) for the usability factor (α=0.88) and credibility and content factor (α=1.00) were high, indicting acceptable reliability of the measure. Analysis showed a significant improvement in the usability score (t10=-2.636, p=0.025) with the means improving from 3.13 (SD±0.59) on the pre-test to 4.10 (SD±0.68) subsequent to the modifications. No significant change was observed in terms of credibility and content (t10=-0.542, p=0.599), with the pre-modification mean being 4.33 (SD±0.51) compared with a post-modification mean of 4.50 (SD±0.55) |
| Anjana Das et al., 2019  United States | To pilot a peer mobilization approach to explore the feasibility of connecting with unreached virtual networks of MSM (men who have sex with men) in Mumbai who may not visit hot spots and promoting HTS (HIV testing services) within these networks. | Pilot study | 247 | Intervention= 247 | HIV (human immunodeficiency virus) - physical health Syphilis - physical health | Mean/median age not provided, only range:  Less than 20 years - n = 70 (28%) 20-24 years - n = 101 (41%) More than 25 years - n = 76 (31%) | Participants identified as Indian men. | In the period January to July 2017, messages on social media were sent to 5,530 MSM and 1,030 MSM made online inquiries. Through social media and coupon referrals, a total of 274 individuals attended the clinic, of whom 27 were ineligible because they either had received targeted intervention services (n=23) or were less than 18 years old (n=4). Thus, 247 MSM were enrolled, which included 22 primary seeds (first-wave peer mobilizers), subsequent waves of peer mobilizers, and others unwilling to be peer mobilizers. The numbers of MSM recruited from each network generated from the 22 primary seeds (not shown) varied greatly. The mean size of the 5 largest networks was 39.8 (range 13–81), while the mean size of 11 networks was 3.8 (range 1–7); 6 primary seeds did not refer others. |
| Anne Looijmans et al., 2019  UK | To determine the effectiveness of a twelve-month multimodal lifestyle approach, including a web-based tool to improve patients’ cardiometabolic health, versus care-as usual. | Randomized controlled trial | 244 | Intervention = 97  Control = 104 | Severe mental illness (such as schizophrenia, other psychotic or bipolar disorders) - Mental health | Intervention = 44.3 (10.9)  Control = 48.6 (10.2) | Not specified | General multilevel linear mixed models adjusted for antipsychotic medication showed that differences in WC change between intervention and control were − 0.15 cm (95%CI: − 2.49; 2.19) after six and − 1.03 cm (95%CI: − 3.42; 1.35) after twelve months intervention; however, the differences were not statistically significant. |
| Astrid Torbjørnsen et al., 2014  United States | To assess whether the use of a mobile health self-management intervention, the Few Touch Application (FTA) diabetes diary [24], with and without a theory-based health counseling intervention, was superior to usual care in terms of glycated hemoglobin A1c (HbA1c) levels, self-management, behavioral change (diet and physical activity), and health-related quality of life after 4 months. Further, the secondary aim was to describe sociodemographic, clinical, and lifestyle characteristics of persons volunteering to participate in such a lifestyle intervention. | Randomized controlled trial | 151 (Data that were not available were considered missing and the results were based on the intention-to-treat approach). | Intervention Arm 1 - FTA: 51 Intervention Arm 2 - FTA with health counseling: 50  Control = 50 | Type 2 diabetes - physical health | Age (years), mean (SD): Intervention Arm 1 - FTA: 58.6 (11.8) years Intervention Arm 2 - FTA with health counseling: 57.4 (12.1) years | Not specified | Data were analyzed from 124 individuals (attrition rate was 18%). The groups were well balanced at baseline. There were no differences in HbA1c between groups after 4 months, but there was a decline in all groups. There were changes in self-management measured using the health service navigation item in the heiQ, with improvements in the FTA group compared to the control group (P=.01) and in the FTA with health counseling group compared with both other groups (P=.04). This may indicate an improvement in the ability of patients to communicate health needs to their health care providers. Furthermore, the FTA group reported higher scores for skill and technique acquisition at relieving symptoms compared to the control group (P=.02). There were no significant changes in any of the domains of the SF-36. Demographic info: There were no statistically significant differences between the groups in terms of the baseline variables, except for rheumatism and depressive symptoms (Table 2). Significantly more participants in the FTA group had rheumatism compared with both of the other groups (n=11, 4, and 3 in the FTA, FTA with health counseling, and control groups, respectively, P=.03). More individuals had depressive symptoms (a CES-D score ≥16) in the control group (n=17) compared with the FTA group (n=10) and the FTA with health counseling group (n=7, P=.045). Of the 151 participants, the mean age was 57 years (SD 12), 62 (41.1%) of participants were women, and 83 (55.0%) had less than 12 years of education. The mean HbA1c was 8.2% (SD 1.1) or 66 mmol/mol (SD 12), the mean BMI was 31.7 kg/m2 (SD 6.0), and 58.1% (75/129) were obese [43]. Only 9 of 131 participants (6.9%) did not receive glucose-lowering medication. In total, almost half of the participants (72/151, 48%) reported 2 or more comorbidities and 36 of 151 (23.8%) reported heart disease. Primary Outcomes and Estimations: In total, 118/151 (78.2%) participants provided HbA1c data at 4 months. There were no statistically significant differences in HbA1c level changes from baseline between the 3 groups (P=.65) after 4 months (Table 4). Adjustments for age, gender, and education did not affect the estimates. The mean HbA1c level declined in all groups: –0.41 (95% CI –0.71 to –0.11) in the FTA with health counseling group, –0.23 (95% CI –0.47 to 0.01) in the FTA group, and –0.39 (95% CI –0.75 to –0.03) in the control group. |
| Benjamin E. et al., 2021  United States | To determine whether the use of patient navigators, in conjunction with social worker support,could increase adherence to recommended follow-up eye appoint-ments | Randomized controlled trial | 344 | Intervention = 172  Control 172 | Physical health - Glaucoma | 60.8 (11.4) | 66.2% African American, 15.4% White, 4.8% Asian, 11.0% Hispanic, More than one race 2.7% | Timely attendance at the first visit was higher for EI than UC (74.4% vs 39.0%; average relative risk [aRR] = 1.85; 95% CI,1.51–2.28; P < .001). Rates of adherence to recommended annual follow-up during year 1 were 18.6% in the EI group and 8.1% in the usual care group (aRR = 2.08; 95% CI, 1.14–3.76; P = .02).The aRR across years 2 and 3 was 3.92 (95% CI, 1.24–12.43; P =.02) |
| Bonnie Spring et al., 2017  United States | The ENGAGED study examined the efficacy of two abbreviated (8 in-person session) versions of the Diabetes Prevention Program (DPP), as compared to a self-guided version. The control self-guided program (SELF) provided DPP sessions on DVD and utilized paper self-monitoring diaries. Having produced modest weight loss in a prior primary care trial,17 SELF has been recommended as low cost weight loss standard of care.18,19 One abbreviated intervention (technology-supported: TECH) used a custom-designed smartphone app for diet and weight self-monitoring, and integrated social media and passively monitored physical activity data from an accelerometer. The other abbreviated intervention (standard: STND) used paper and pencil self-monitoring. Both TECH and STND were hypothesized to produce greater weight loss than SELF because they included in-person group treatment sessions.20 Based on previous research showing greater self-monitoring with technology relative to paper methods,21 TECH was hypothesized to yield better self-monitoring adherence and therefore greater weight loss than STND. | Randomized controlled trial | 96 | Intervention = 32  Control = 32 | Obesity (intervention for weight loss) - physical health | Intervention Age, mean (SD) (y): 40.4 (10.7)  Control Age, mean (SD) (y): 37.3 (13.3) | Ethnicity of all participants (control + 2 intervention groups): Hispanic or Latino: 19.8% Not Hispanic or Latino: 80.2% Race of all participants (control + 2 intervention groups): White: 57.3% Black or African American: 31.3% | Weight loss was greater for TECH and STND than SELF at 6 months [−5.7kg (95% CI: −7.2, −4.1) vs. −2.7kg (95% CI: −5.1, −0.3), p<.05]), but not 12 months. TECH and STND did not differ except that more STND (59%) than TECH (34%) achieved ≥5% weight loss at 6 months (P < 0.05). Self-monitoring adherence was greater in TECH than STND (P <0.001), greater in both interventions than SELF (P <0.001), and covaried with weight loss (r(84) = 0.36 − 0.51, P<.001). Study Participants: At baseline, participants had a mean (SD) age of 39.3 (11.7) years and BMI of 34.6 (3.0) kg/m2. Eighty-four percent were female; 57.3% were white, 31.3% were black. Baseline participant characteristics appear in Table 1. The treatment groups showed no baseline differences on age, sex, race, ethnicity, marital status, education, or weight. Attrition at the final 12 month follow-up assessment was greater for SELF (25.0%) than either STND (12.5%) or TECH (3.1%) treatments (p = .02), and was not differential between the STND and TECH treatments (p=.20) (Figure 1). Treatment Fidelity: Across the study’s duration, 105 coaching calls were assessed for treatment fidelity from 46 participants. Overall, fidelity was 95.0%; three coach re-trainings were held. Weight Loss: At 12 months, participants showed a mean ± 95% CI weight change from baseline of −5.6(−8.5, −2.8) kg in STND, −3.1(−5.9, −0.3) kg in TECH, and −2.7(−5.7, 0.4) kg in SELF (Table 2). When TECH and STND were combined, weight change was significantly greater than SELF at 3 (P < 0.005) and 6 months (P < 0.05), but not 12 months (Table 2 and Figure 3). When measured as a continuous variable, there was no difference in weight change between TECH and STND at any time point. At 6 months, weight loss of at least 5% occurred more often in the experimental treatments (47%) than in SELF (13%) (P < 0.005) and more often in STND (59%) than TECH (34%) (P <0.05). At 12 months, weight loss of at least 5% was observed in 47% of STND, 28% of TECH and 25% of SELF participants; these differences were not significant. Self-Monitoring Adherence: Table 3 compares adherence to diet, physical activity, and weight self-monitoring across the three treatment conditions during the 6 months of the intervention. Diet, activity, and weight self-monitoring were greater in TECH and STND than SELF (P <0.001). Self-monitoring of all behavioral outcomes also was greater in TECH than STND: diet (P <0.05), activity (P<0.001), and weight (P <0.001). The amount of weight loss at 6 months covaried with the amount of self-monitoring of diet [r(84)=.509, p<.001], physical activity [r(84)=.460, p<.001], and weight [r(84)=.364, p<.001]; the correlations did not differ as a function of treatment condition. |
| Boris Hansel et al., 2017  Canada | The objective of the study was to evaluate a fully automated Web-based intervention designed to help users improve their dietary habits and increase their physical activity. | Randomized controlled trial | 120 | Intervention = 40 - At the end of the study, 13 subjects (8 in theintervention arm and 5 in the control arm) were unable to becontacted to collect dietary data, and 17 subjects (12 in theintervention arm and 5 in the control arm) did not attend thelast visit.  Control = 60 | Physical health - T2DM and abdominal obesity | Intervention = 57.6 (8.1)  Control = 55.5 (10.3) | Not specified | Using an intention-to-treat analysis, the DQI-I score (54.0, SD 5.7 in the ANODE arm; 52.8, SD 6.2 in the control arm; P=.28) increased significantly in the ANODE arm compared to the control arm (+4.55, SD 5.91 vs -1.68, SD 5.18; between arms P<.001). Body weight, waist circumference, and HbA1c changes improved significantly in the intervention. The DQI-I score was 54.0 (SD 5.7) in the e-coaching arm and 52.8 (SD 6.2) in the control arm (P=.28 between arms). The intention-to-treat analysis at 16 weeks showed that e-coaching resulted in a significant improvement in the DQI-I score: +4.55 (SD 5.91) in the intervention arm and-1.68 (SD 5.18) in the control arm (P<.01 between arms; Table2). Moreover, changes in dietary intake tended to differ between arms for lipids (P=.02), saturated fats (P<.01), sodium (P=.07),and empty calories (P=.06), always towards healthier foods in the intervention arm. Compared to usual care, the ANODE program was associated with reduced body weight, waist circumference, and HbA1c(Multimedia Appendix 2). A significantly higher proportion of e-coaching subjects achieved weight loss >3% (20/60, 33.3%of e-coaching subjects vs 4/60, 6.7% of control subjects on intention-to-treat analysis; P<.01) and weight loss >5% (12/60,20.0% vs 2/60, 3.3% on intention-to-treat analysis; P<.01;Figure 3). Only two subjects (2/60, 3.3%) in the ANODE arm and no subject in the control arm achieved >10% weight loss, respectively (P=.15 between arms).No significant differences in terms of change in blood pressure, plasma lipids, amino transferases, gamma glutamyl aminotransferase, uric acid, fasting glucose, VO2max or hs-CRP were observed between the two arms at 4 months |
| Carmen G. et al., 2013  Germany | Purpose: Individuals with cancer frequently report significant gaps in information, support, and health care service navigation at diagnosis and in the survivorship phase. A comprehensive web-based tool called the Oncology Interactive Navigator™(OIN) appears promising in addressing these gaps. The present qualitative inquiry explores the perceptions of individuals concerning the OIN™, as a complementary resource to support psycho-social adjustment to cancer and guide access to cancer care services. | Qualitative research | 20 | Intervention = Colorectal cancer: 11 Melanoma: 9 | Physical health: Colorectal cancer (newly diagnosed) Melanoma (newly diagnosed) | Colorectal cancer: Mean age=60.2 years (no standard deviation provided). Melanoma: Mean age=58.9 years (no standard deviation provided). | Not specified | Theme 1 - a valuable comprehensive, readily accessible, and reliable source of cancer information and support  Sub-themes for Theme 1 1 - Comprehensive 2 - A complement to information received from HCPs 3 - A means of cross-verifying cancer information 4 - Institutionally supported and reliable 5 - Accessible 24/7  Theme 2 - a useful means to control/pace exposure to cancer information  Sub-themes for Theme 2 1 - Access to the right amount and type of cancer information 2 - Ability to partition cancer information into manageable chunks 3 - Preference for early introduction  Theme 3 - a catalyst for cancer knowledge acquisition and understanding among patients and family members.  Sub-themes for Theme 3 1 - Improving patients’ and family members’ knowledge and understanding of cancer 2 - Consequences of improved cancer knowledge |
| Cassidi D. Kalejta et al., 2019  United States | To evaluate the impact of this automated approach on patient comprehension, patient satisfaction, time, savings, and cost. | Other: Unclear | 8965 | Intervention = 8965 | Prenatal/pregnancy - Physical health | Not specified | Not specified | Of 12,891 patients who underwent NIPT at SCPMG between April 2017 and December 2018, 10,170 (79%) elected to enroll in the automated workflow. Of the registered patients, 8,965 (88%) completed the automated workflow as determined by the suc‐ cessful download of their test result. The remaining 12% (1,205) of patients did not complete the automated workflow which we hypothesized could be due to a variety of reasons including having a positive NIPT, of which the patient would have been notified by the ordering provider; or the patient entered referral information incor‐ rectly which would not allow the system integration to match the patient's test result with their Maven account. Of the 12%, approxi‐ mately 7% (80) of the patients viewed the negative result disclosure video but did not download the NIPT result. We hypothesized that this cohort of patients perhaps did not want to learn the predicted chromosomal sex of the baby. Of the 8,965 that completed the process, 8,855 watched the video and answered the comprehension questions once, while the remain‐ ing 110 watched the video and answered the questions multiple times. Of the 8,965 that com‐ pleted the process, 2,121 (24%) completed the survey. The ma‐ jority of patients, 1,762 of the 1,961 respondents, were between the ages of 31 and 40 (90%) with an average household income of greater than $100,000 for 774 of the 1,832 (42%) respondents |
| Charlene C. et al., 2011  United States | The Mobile Diabetes Intervention Study, reported here, evaluated a diabetes-coaching system, using mobile phones and patient/provider portals for patient-specific treatment and communication. The hypothesis tested was that mobile telephone feedback on self-management of blood glucose results and lifestyle and clinical management offered to patients with type 2diabetes and their providers can reduce glycated hemoglobin levels over 1 year. | Randomized controlled trial | 163 | Intervention 1: 23 Intervention 2: 22 Intervention 3: 62  Control = 56 | Type 2 diabetes - physical health | Intervention  Mean (SD) age (years): Intervention 1: 52.8 ± 8.0 Intervention 2: 53.7 ± 8.2 Intervention 3: 52 ± 8.0  Control Mean (SD) age: 53.2 ± 8.4 years | Intervention 1: Black (non-Hispanic): 43.5% White (non-Hispanic): 52.2% Other: 4.3%  Intervention 2: Black (non-Hispanic): 45.5% White (non-Hispanic): 40.9% Other: 13.6%  Intervention 3: Black (non-Hispanic): 27.4% White (non-Hispanic): 62.9% Other: 9.7%  Control: Black (non-Hispanic): 48.2% White (non-Hispanic): 46.4% Other: 5.4% | The mean declines in glycated hemoglobin were 1.9% in the maximal treatment group and 0.7% in the usual care group, a difference of 1.2% (P<0.001) over 12 months. Appreciable differences were not observed between groups for patient-reported diabetes distress, depression, diabetes symptoms, or blood pressure and lipid levels (all P>0.05). The 163 study patients had a mean baseline glycated hemoglobin of 9.4% (range 7.5–15.5) (Table 1). Mean age was 52.8 years, 50.3% were female,39.3% were African American, and 31.3%were college educated. The mean duration of diabetes was 8.2 years. Most participants(76.1%) were obese (BMI$30 kg/m2).Participants had a mean PHQ-9 of 5.2(minimal to mild depression scores). Most participants had hypertension (63.2%)and hypercholesterolemia (58.3%). CPDS patients had higher baseline glycated hemoglobin than UC (9.9 vs. 9.2%,P=0.04). No other baseline patient variables differed significantly among the four study groups. |
| Charles R. Jonassaint et al., 2017  UK | We studied differences in CCBT use and self-reported change in depression and anxiety symptoms among 91 African Americans and 499 White primary care patients aged 18–75, enrolled in a randomised clinical trial of collaborative care embedded with an online treatment for depression and anxiety This trial examined whether a collaborative care approachdelivering CCBT, either alone or combined with an InternetSupport Group (ISG), is effective for treating mental health in aprimary care setting. | Randomized controlled trial | 590 | Not specified. Study participants are listed in African American vs White participants. African American - 91, White - 499 | Mental health - Depression and anxiety | Not specified. Study participants are listed in African American vs White participants. Age of African American - 39.9 (13.9), White 43.6 (14.2) | African American and White | Compared with White participants, African Americans were less likely to start the CCBT programme (P=0.01), and those who did completed fewer sessions and were less likely to complete the full programme (P=0.03). Despite lower engagement, however, African Americans who started the CCBT programme experienced a greater decrease in self-reported depressive symptoms (estimated 8-session change:−6.6v.−5.5;P=0.06) and similar decrease inanxiety symptoms (−5.3v.−5.6;P=0.80) compared with White participants. Of the 2884 patients PCPs referred, 954 met eligibility criteria, 704 consented and were randomised, and 601 were assigned to either of the two groups with CCBT access (91 African American, 499 White, 11 other). Compared with White people, African Americans were younger, had higher baseline PHQ-9 scores and were less likely tobe using pharmacotherapy at study entry (76%v.90%,P<0.01;) |
| Christine Sawicki et al., 2019  United States | In 2016, CVS Specialty implemented 2-way clinical messaging technology (provider messages the patient, patient can in turn message the provider). Patients receive personalized messages based on their specific clinical and medication profiles. With receipt of each message, patients have the opportunity to ask questions and communicate difficulties or successes. We hypothesized that using discreet clinical messages would enhance communication and would be more effective than 1-way texting in improving adherence to TKIs among CML patients. | Cohort study | 558 | Intervention = 279  Control = 279 | Patients who were initiated on TKI therapy (imatinib mesylate, dasatinib, bosutinib, or nilotinib) for treating chronic myeloid leukemia (cancer) - physical health | Intervention = Age at first fill, mean ± SD = 53.3 (13.3) years  Control = Age at first fill, mean ± SD = 54.4 (14.5) years | Intervention: % African American, mean ± SD = 12.7% (16.9)  Control: % African American, mean ± SD = 13.7% (20.3) | Patients receiving clinical messaging had on average a 7.64% higher MPR score (MPR: 73.94% vs. 66.30%) compared with the control arm (P=0.0063). This translates to 22% more patients being optimally adherent while exposed to clinical messaging (P=0.022). Patients in the exposed group had a mean 32-day increase in average length of therapy compared with the control group (243 days vs. 275 days, P=0.0043), potentially driving an increase in adherence. Additional drivers included a 5.4 percentage point reduction in first fill drop-off rates (4.66% vs. 10.04%, P=0.0149). Persistency after 12 months was similar between the study arms (41%). |
| Christophe JP Smeets et al., 2018  Canada | The aim of this study was to verify whether supplementing the usual care with the CardioCoach follow-up tool is feasible and safe, and whether the tool is more efficient in implementing the guideline recommendations for β-blocker and ACE-I. | Randomized controlled trial | 24 | Intervention = 14  Control = 10 | Heart disease - Physical health | Intervention 63 (15)  Control 60 (15) | Not specified | Patients’ satisfaction and adherence for medication intake (10,018/10,825, 92.55%) and vital sign measurements (4504/4758, 94.66%) were excellent. However, the number of technical issues that arose was large, with 831 phone contacts (median 41, IQR 32-65) in total. The semiautomatic remote uptitration was safe, as there were no adverse events and no false positive uptitration proposals. Although no significant differences were found between both groups, a higher number of patients were on guideline-recommended medication dose in both groups compared with previous reports. |
| Chul Hyun et al., 2020  United States | This study aimed to assess the efficacy of a newly developed mobile texting app (HepTalk) in overcoming these barriers and improving patient engagement and health care access among HBV-infected and non-immune individuals. Objective is to further examine the efficacy of mobile texting in engaging patients in collaborating in their own viral hepatitis B care, we developed a text messaging app, HepTalk. We evaluated the effects of its use in two groups of individuals similar to those in our previous study: people with CHB, who are not currently accessing care and thus need to see HCPs for further evaluation; and nonimmune individuals who need vaccination at a health care facility. | Non-randomized experimental study | 82 | Intervention - CHB group 21  Control = Nonimmune group 61 | Physical health - Chronic hepatitis B (CHB) | Intervention CHB group: Mean 54  Control Nonimmune group: Mean 47 | Korean-American participants | On average, patient navigators sent and received 14 and 8 messages per participant, respectively, during the 6-month period. The themes of the messages were similar to the following 4 categories: finding providers, scheduling appointments with providers, health education, and financial issues. Of the 82 participants, 78 were linked to care within 6 months (a 95% linkage rate). |
| David Ebert et al., 2013  Switzerland | The purpose of the current study is to evaluate the effectiveness of a recently developed transdiagnostic Inter-net-based maintenance treatment (TIMT) [22] when compared to treatment as usual (TAU) [23]. We tested the hypothesis that TIMT + TAU participants will display significantly better maintenance of improvement after discontinuing inpatient treatment than will participants in a TAU-only condition. | Randomized controlled trial | 400 | Intervention = 200 - Although not all participants reached the end...aiming at an intention-to-treat design, we included all participants who were randomly assigned to either condition.  Control = 200 | Affective, neurotic, and/or behavioral disorders - mental health | Intervention = Mean (SD) - 45 ± 8.88 years  Control = Mean (SD) - 45 ± 9.80 years | Nationality provided - German: 99% for intervention and 97% for control | Primary Outcome - Change in Psychopathological Symptoms: As expected, there was (a) a significant decrease in GPS between t1 and t2 in both conditions (with no interaction between T1–T2 and treatment condition), (b) a significant increase in GPS in the TAU-only condition between t2 and t3 (as indicated by the significantly positive regression coefficient for T2–T3 in the TAU-only condition), which was significantly attenuated in the TIMT + TAU condition (as indicated by the significant T2–T3 × condition interaction effect), and (c) a significant increase in GPS in the TAU-only condition between t2 and t4 (as indicated by the significantly positive regression coefficient for T2–T4 in the TAU-only condition), which was significantly attenuated in the TIMT + TAU condition (as indicated by the significant T2–T4 × condition interaction effect). Effect sizes were small to medium for the difference in change between conditions at 3-month follow up and medium for differences in change between conditions at 12-month follow-up.  Reliable Change, Remission, and Recovery: At each follow-up assessment, the TIMT + TAU group consisted of significantly less patients with reliable symptom deteriorations than the TAU-only group. At 3-month follow-up, TIMT + TAU patients had a 71% lower risk of being deteriorated than TAU-only patients (relative risk = 0.29) and a 61% lower risk at 12-month follow-up (relative risk = 0.39). The number of patients needed to treat to reduce the amount of deteriorations from discharge to 12-month follow-up by 1 was 5.56. Moreover, significantly more patients of the TIMT + TAU group were in remission at each follow-up than in the TAU-only group ( table 4 ). TIMT + TAU patients were 68% more likely to be remitted at 3-month followup than TAU-only patients (odds ratio = 1.68), and they were 121% more likely to be in remission at 12-month follow-up (odds ratio = 2.21). Furthermore, after having achieved remission, significantly more TIMT + TAU participants were still remitted at 12-month follow-up and achieved recovery compared to TAU-only patients. TIMT + TAU participants were 73% more likely to be recovered at 12-month follow-up than TAU-only patients (odds ratio = 1.73). |
| Dayana M et al., 2019  Ireland | We believe that the development of an educational mobile app to help women perform PFMT, such as the Continence App, would reduce adherence barriers and facilitate its execution by them. In this context, the aim of this study was to describe the development of the Continence App as an educational technology to promote PFMT adherence and to prevent UI in postpartum women. | Other: Study of technological production carried out in two stages: construction and validation | N/A. | N/A. | Postnatal urinary incontinence prevention - physical health | N/A. | N/A. | Most of health experts were physiotherapists (n = 07) and professionals with a PhD (n = 07). Regarding the CVI, 100% of the evaluated items obtained values of 0.86 or greater. Most of information technology/computing/communication experts were male (81.8%) and of the information technology area (63.6%). Except for “restart sessions”, “ways of presenting suggestions”, “user interaction” and “motivates questioning”, all the other aspects received between 81.8% and 100% positive responses. All items evaluated by the target audience obtained a minimum of 94.3% positive responses from the participants. In this step, points for adjustment were identified in relation to the application content and interface, which were promptly corrected. |
| Deanna Kerrigan et al., 2019  United States | To determine the impact of a community empowerment model of combination HIV prevention (Project Shikamana) among female sex workers (FSW) in Iringa, Tanzania. | Randomized controlled trial | 387 | Intervention HIV positive: 171  Control HIV negative: 216 | HIV - Physical health | Intervention HIV positive: 30.20 (7.2)  Control HIV negative: 25.90 (5.8) | Female sex workers (FSW) in Iringa, Tanzania. | The analysis included 171 HIV-positive and 216 HIV- negative FSW who completed baseline and 18-month study visits. Participants in the intervention were significantly less likely to become infected with HIV at 18-month follow-up (RR 0.38; P = 0.047), with an HIV incidence of 5.0% in the intervention vs. 10.4% control. Decreases in inconsistent condom use over time were significantly greater in the intervention (72.0%–43.6%) vs. control (68.8%–54.0%; RR 0.81, P = 0.042). At follow-up, we observed significant differences in behavioral HIV care continuum outcomes, and positive, but nonsignificant, increases in viral suppression (40.0%–50.6%) in the intervention vs. control (35.9%–47.4%). |
| Deborah Morrison et al., 2015  UK | Aimed to develop an evidence based, theory informed, online resource to support self-management in adults with asthma, called ‘Living well with Asthma’, as part of the RAISIN (Randomized Trial of an Asthma Internet Self-Management Intervention) study. To describe the development and optimisation of the Living well with Asthma website which we developed using the LifeGuide open access software package. | Other: Article aims to describe the development of the Living well with Asthma website | N/A | N/A | Asthma - Physical health | N/A | Focus groups were conducted with White British individuals | The website asked users to aim to be symptom free. Key behaviours targeted to achieve this include: optimising medication use (including inhaler technique); attending primary care asthma reviews; using asthma action plans; increasing physical activity levels; and stopping smoking. The website had 11 sections, plus email reminders, which promoted these behaviours. Feedback on the contents of the resource was mainly positive with most changes focussing on clarification of language, order of pages and usability issues mainly relating to navigation difficulties. |
| Donna L Berry et al., 2014  UK | The purpose of this analysis was to compare verbal reports of SxQOL (symptoms and quality of life issues) between the study groups with regard to: 1) reported severity, pattern, alleviating/aggravating factors and requests for help for the full set of 26 ESRA-C SxQOL issues; and 2) reports of individual SxQOL issues within the full set, plus 3) to determine whether any observed differences would account for differences in symptom distress. | Other: Analysis of the results of a prior prospective randomized controlled trial (ESRA-C II) | 517 | Intervention = 256  Control = 261 | Cancer (different types) - physical health: | Intervention = Median (range): 55 (22-86) years  Control = Median (range): 59 (22-87) years | Not provided/included. | Patients with audio-recordings were younger in the intervention group than in the control group (p < .0001). Out of 517 patients, 27 (13 control and 14 intervention) did not discuss any problematic SxQOL issue during the clinic visits. There was no significant difference (p = 0.41) between study groups in number of problematic SxQOL issues discussed at all during clinic visits, with a median of 4 issues discussed by control group patients, and 3 by patients in the intervention condition. Patients initiated general discussion of an average 56% of problematic SxQOL issues in the control group and 55% in the intervention group (p =0.97). Family members initiated 4% of the problematic SxQOL issues in the control group and 5% in the intervention group (p = 0.35)...The percentage of problematic SxQOL issues which patients or caregivers reported using any specific coached statement during the clinic visit, was significantly higher (p = 0.002) in the intervention group than that in the control: a median of 85% of problematic SxQOL were reported as coached in the intervention group versus 75% in the control (Table 2). After adjusting for covariates, group remained significantly associated (p = 0.0009); intervention group patients had an approximate 9% higher rate of describing problems with a coached statement (Table 3). Discussion Section: The patients in the ESRA-C II randomized trial who received an educational coaching intervention to aid verbal report of problematic SxQOL applied the reporting framework as coached (severity, pattern, aggravating/alleviating factors and help request), reporting these specific details without prompting, significantly more often than control group patients. When examining individual SxQOL issues, we found that reports for the majority of individual SxQOL issues were more frequent in the intervention group. Even though our study was not powered to compare individual SxQOL issues, we found that fatigue, pain and physical function were reported significantly more often by the intervention group. Conclusion Section: Electronic education and coaching provided to patients with a variety of cancers of all stages resulted in significantly more specific verbal reports of SxQOL concerns made to treating clinicians in face to face visits. Results from Abstract (may be duplicate): 517 (256 intervention) clinic visits were audio-recorded. General discussion of problematic SxQOL was similar in both groups. Control group patients reported a median 75% of problematic SxQOL using any specific coached statement compared to a median 85% in the intervention group (p = .0009). The median report index of coached statements was 0.25 for the control group and 0.31 for the intervention group (p = 0.008). Fatigue, pain and physical function issues were reported significantly more often in the intervention group (all p < .05). Clinicians' verbalized responses did not differ between groups. Patients' verbal reports did not mediate final SD outcomes (p = .41). |
| Donna M. Zulman et al., 2015  United States | Despite the growing availability of eHealth technology, it is unclear whether existing tools are meeting the needs of patients with high levels of illness burden. The US Department of Health and Human Services has recommended enhancement of health information technology, including eHealth tools, to improve care for patients with MCCs.22 To inform these efforts, we conducted a qualitative study of patients with MCCs who use technology for health-related purposes. Our objectives were to (1) understand self-management and health care navigation challenges that patients face because of their multiple health problems and (2) identify opportunities to enhance existing technology, or develop new applications and tools, to better support these patients. | Qualitative research | 53 | Intervention = 53 | Multiple chronic conditions*† (Conditions reflect the ten most common conditions reported by patients. A full list of conditions is available in Appendix 4) - both physical and mental health:  High blood pressure - n=40 (75%)  Chronic pain - 34 (64%)  Arthritis/rheumatism - 26 (49%)  Depression - 21 (40%)  Diabetes - 18 (34%)  Headaches/migraines - 17 (32%)  Post-traumatic stress disorder - 16 (31%)  Lung/breathing problem - 12 (23%)  Cancer - 9 (17%)  Prostate problems - 8 (15%) | Mean (SD): 59 (11) years | Race (N=52)* Individuals could indicate more than one response item White, non-Hispanic - n=43 (81%) Black, non-Hispanic - 3 (6%) Hispanic - 5 (9%) Other, non-Hispanic - 7 (13%) | Demographic info: Participants in the ten focus groups (n=53) had a mean (SD) age of 59 (11), 74 % were male, and 43 % had an annual household income<$50,000. Participants reported receiving primary care within a range of health care systems, including the VA system (51 %), academic-affiliated clinics (34 %), and Kaiser (a large health maintenance organization) or community clinics (19 %) (Table 1). The mean (SD) number of chronic conditions among participants was five (2), with the most common conditions including hypertension (75 %), chronic pain (64 %), arthritis (49 %), depression (40 %), diabetes (34 %), post-traumatic stress disorder (31 %), lung disease (23 %), cancer (17 %), and heart failure (13 %) (Table 1). Most participants reported using technology for health-related purposes daily to several times per week (51 %) or several times per month (40 %). The most common reasons for using technology included searching for health information (96 %), communicating with a health care provider (92 %), tracking medical information (83 %), tracking medications (77 %), and supporting decision-making about treatment (79 %) |
| Elizabeth L. Clemens et al., 2018  United States | This study was designed to test the use of wireless smart-phone HBPM to improve BP control among patients with newly diagnosed or persistently uncontrolled HTN. The study protocol included the enhanced use of electronic health records (EHRs) to allow care providers to easily monitor their patients’ home measurements. The authors hypothesized that patients’ awareness of their disease, facilitated by easy and frequent self-monitoring, and their healthcare providers’ access to enhanced information would improve this population’s HTN control. | Cohort study | 484 | Intervention Total sample size (across four sites): 131  Control Total sample size (across four sites): 353 | New or existing uncontrolled hypertension (HTN) - physical health | Intervention = Only provided n (size) of age group, +/- 60 years,*n(%) - 48 (36.6%)  Control = Only provided n (size) of age group, +/- 60 years,*n(%) - 210 (59.5%) | Listing only for intervention arm:  White - n=107 (84.3%) Black - 14 (11.0%) Other - 6 (4.7%) | The total study population included 484 patients with mean age 60 years (range 23–102 years),47.7% female, and 84.6% Caucasian. Mean preintervention BP was 137.8mm Hg systolic and 81.4 mm Hg diastolic. Mean (average) BP control rates improved for patients who received HBPM from 42% to 67% compared with matched control patients who improved from 59% to 67% (p<0.01).  Data were collected from October 2014 to May 2015; 131patients were included in the analyses, and no patients were excluded due to out-of-range arm circumference.  Thirty-seven patients with 17 unique providers, representing 3 clinic sites, participated at the study site; 94 additional patients were enrolled in parallel quality improvement projects at the other 3 clinics, followed similar protocols, and used the identical device. A total of 353 matched control patients were included for comparison. |
| Elyse R. Park et al., 2020  United States | To adapt and assess the virtual delivery of a mind-body group resiliency program, the Stress Management and Resiliency Training-Relaxation Response Resiliency Program (SMART-3RP), to meet the needs of parents of children with learning and attentional disability (LAD) | Other: Mixed methods study - 2 phases | Phase 2: 54 | Intervention - Phase 2: 18  Control - Phase 2: 21 | Mental health - stress | Intervention - Phase 2: 47 (6.2)  Control - Phase 2: 47 (4.9) | Phase 2: White: 90.6%, Asian: 3.8%, Black or African American (3.8%) | Fifty-three parents (mean age = 46.8; 90.6% female) participated nationally in the pilot trial. 62.5% of participants completed ≥ 6/8 sessions; 81.8% reported continued daily/weekly relaxation response exercise practice. T1–T2 comparisons found that IG versus WC participants showed sig- nificant improvements in distress [VAS], ∆M = − 1.95; d = .83 and resilience [CES], ∆M = 6.38; d = .83, as well as stress coping [MOCS-A] ∆M = 8.69; d = 1.39; depression and anxiety [PHQ-4], ∆M = − 1.79; d = .71; social support [MOS-SSS], ∆M = 5.47; d = .71; and empathy [IRI], ∆M = 3.17; d = .77; improvements were sustained at the 3 month post intervention follow-up.  Participants (n = 53) attended a median of 6 out of 8 ses- sions; 62.5% of participants completed ≥6/8 sessions, and participation rates were similar across both groups. Among enrolled participants, 40 (75.5%) completed the time 2 sur- vey and 35 (66.0%) completed the time 3 survey. The larg- est proportion of dropout (38.7%) occurred in the interven- tion group condition between time 1 and time 2, with one group conducted in the late spring which overlapped with the end of the school year. Study completers (n = 40) and non-completers (n = 13) did not differ by demographic factors assessed at enrollment. |
| Emily S Ross et al., 2021  Canada | We report on a pilot study of a one-way SMS text messaging intervention (Txt2Prevent) aimed at supporting patients with ACS after hospital discharge in an assessor-blinded randomized controlled trial. The study’s primary objective was to assess the effect of the Txt2Prevent intervention on self-management domains compared with usual care. The other objectives were to compare quality of life, self-efficacy, medication adherence, and health care resource use between the 2 groups as well as to assess the feasibility of the study protocol and acceptability of the Txt2Prevent intervention. | Randomized controlled trial | 76 (37+38=75; 1 participant excluded from analysis, admitted for noncardiac reason - intent-to-treat) | Intervention = 37  Control = 38 | Acute coronary syndrome (ACS) - physical health | Intervention = Age, mean (SD) years: 59.5 (9.1)  Control = Age, mean (SD) years: 61.1 (9.6) | Not specified | There were no statistically significant differences between the groups for the heiQ domains (adjusted mean difference [Txt2Prevent minus usual care] for each domain—Health-directed activity: –0.13, 95% CI –0.39 to 0.13, P=.31; Positive and active engagement in life: 0.03, 95% CI –0.19 to 0.25, P=.76; Emotional distress: 0.04, 95% CI –0.22 to 0.29, P=.77; Self-monitoring and insight: –0.14, 95% CI –0.33 to 0.05, P=.15; Constructive attitudes and approaches: –0.10, 95% CI –0.36 to 0.17, P=.47; Skill technique and acquisition: 0.05, 95% CI –0.18 to 0.27, P=.69; Social integration and support: –0.12, 95% CI –0.34 to 0.10, P=.27; and Health services navigation: –0.05, 95% CI –0.29 to 0.19, P=.69). |
| Estelle Everett et al., 2018  Canada | The objective of this pilot study was to calibrate the Sweetch app and determine the feasibility, acceptability, safety, and effectiveness of the Sweetch app in combination with a digital body weight scale (DBWS) in adults with prediabetes | Other: Prospective, single-arm, observational study | 55 | Intervention = Calibration cohort: 9, Intervention Cohort: 38 | Prediabeties/Diabetes - Physical health | Calibration cohort: 57.6 (0.9), Intervention Cohort: 57.2 (-9.1) | Calibration cohort: White: 44% Black: 56%, Other: 0%, Intervention Cohort: White: 82%, Black: 18%, Other: 0% | The study retention rate was 47 out of 55 (86%) participants. There was a high degree of acceptability of the Sweetch app, with a median (interquartile range [IQR]) score of 78% (73%-80%) out of 100% on the validated System Usability Scale. Satisfaction regarding the DBWS was also high, with median (IQR) score of 93% (83%-100%). PA increased by 2.8 metabolic equivalent of task (MET)–hours per week (SD 6.8; P=.02), with mean weight loss of 1.6 kg (SD 2.5; P<.001) from baseline. The median change in A1c was −0.1% (IQR −0.2% to 0.1%; P=.04), with no significant change in fasting blood glucose (−1 mg/dL; P=.59). There were no adverse events reported. |
| Eun-Shim Nahm et al., 2019  United States | To evaluate an interactive electronic Cancer Survivorship Patient Engagement Toolkit (CaS-PET) | Other: Single-group pre-/post-test deign. | 30 | Intervention = 30 | Physical health - Cancer | 56.5 (SD 13.6) | Black: 18, White: 10, Other: 2 | At the end of the intervention, participants were asked about their perceptions of the usefulness of SCPs. and WBC. The former was assessed using one item with a yes/no response option; the latter was assessed using a three-item usefulness sub scale of the Health Web Site Usability Questionnaire on a seven-point Likest-type scale from 1 (strongly disagree) to 7 (strongly agree). In addition, experiences with using the WBC program and SCPs were assessed using open-ended questions. |
| Eva Haukeland Fredriksen et al., 2016  Canada | The aim of this study was to understand how Web-based discussion forums influence maternal health literacy; hence, we explored the role of interactions in Web-based discussion forums among women who experienced health problems during pregnancy. More specifically, we explored why media-literate women experiencing the medically unexplained condition, pelvic girdle pain (PGP), during pregnancy participated in Web-based discussion forums and how they appraised and applied the information and advice that they gained from the Web-based interaction with other women. | Qualitative research | 11 | Intervention = 11 | Pregnancy/PGP - Physical health | Age range: 22-39 | Not specified | In our study sample, The interaction in Web-based discussion forums influenced maternal health literacy in terms of increased health-related knowledge and competencies, increased awareness of health promotion and health protection, and increased system navigation. The women appraised and selectively applied information and advice that resonated with their own experiences. For many, the information provided online by other women in the same situation was valued more highly than advice from health professionals. Women reported that they used their knowledge and competency in encounters with health professionals but hesitated to disclose the origin of their knowledge. Those with a high level of education in medicine-related fields raised a concern about the Internet as a source of horror stories and erroneous information and were actively engaged in trying to minimize potential negative effects, by providing biomedical information. |
| Francisco Monteiro-Guerra et al., 2020  Canada | This study aimed to execute the design process and early prototype evaluation of a personalized PA coaching app for posttreatment breast cancer survivors. In particular, the study explored a design combining behavioral theory and tailored coaching strategies. | Other: Not specified - The paper describes the 3 design stages of the tool: (1) user and context research, (2) app conceptualization and early prototyping, and (3) prototype testing. | Study 1: 14 Study 2: 8 | InterventionStudy 1: 14 Study 2: 8 | Breast cancer - physical health | study 1: 52.8 (SD8.8) study 2: 48.4 (SD8.0) | Not specified | The design process has led to the conceptualization of a personalized coaching app for walking activities that addresses the needs of breast cancer survivors. The main features of the tool include a training plan and schedule, adaptive goal setting, real-time feedback and motivation during walking sessions, activity status through the day, activity history, weekly summary reports, and activity challenges. The system was designed to measure users’ cadence during walking, use this measure to infer their training zone, and provide real-time coaching to control the intensity of the walking sessions. |
| Harm L. Ormel et al., 2018  Germany | This study aimed to examine whether using RunKeeper to increase self-reported PA is feasible in cancer patients and to evaluate patients’ opinion about using RunKeeper in a 12-week program. | Randomized controlled trial | 32 | Intervention = 16  Control = 16 | Cancer - Physical health | Intervention 35.3 (12.9)  Control 31.9 (9.4) | Not specified | Patient mean age was 33.6 years. Between T0 and T1, an increase in PA of 51% (medium estimated effect size r = 0.40) was found in PASE sum score in the intervention group compared with usual care. In addition, total minutes of PA increased with 46% (r = 0.37). These effects decreased over time (T2). Sedentary time decreased with 19% between T0 and T1 and 27% between T0 and T2. Usability was rated "good" and most patients found RunKeeper use helpful to improve PA.  4 qualita- tive semi-structured interviews were performed (see Supplementary Table S3). Two patients did not respond on our telephone calls and did not participate in this part. Most patients (n = 12) were enthusiastic about the RunKeeper app use. Eleven patients were still frequently using the RunKeeper app to self-monitor PA at T2. |
| Heewon Kim et al., 2020  UK | This study investigates the emerging patterns of patient-entered communication (PCC) that occur in an mHealth-based diabetes prevention program for older adults. | Qualitative research | 30 | Intervention = 30 | Diabetes - physical health | 68.17 (2.63) | Not specified | Coaches’ primary PCC strategies included: (a) triggering reflections on users’ routinized habits, (b) jointly determining a measurable health goal, (c) facilitating self- evaluations on recent behavior change, and (d) tailoring programs to adapt to users’ lifestyle or health status. |
| Hong Xiao Sara R. et al., 2015  United States | Despite the potential for wellness coaching to improve lifestyle behaviors for patients with prediabetes and to delay or prevent the onset of diabetes, little is known about optimal approaches to encourage the uptake of wellness coaching in populations of people with prediabetes. This evaluation was conducted as part of the Natural Experiments for Translation in Diabetes Study, which tests the effectiveness of population-targeted diabetes prevention and control policies. The goal of this randomized encouragement trial was to examine the effectiveness of different methods of encouraging patients with prediabetes to use the Wellness Coaching Center (WCC) at Kaiser Permanente Northern California (KPNC).We hypothesized that 3 outreach interventions (secured email message, telephone message, and letter) would increase the up-take of wellness coaching among KPNC patients with prediabetes. | Randomized controlled trial | 14584 | Intervention 1 of 3 (secure email message): 2667 Intervention 2 of 3 (interactive voice response - telephone message): 2675  Control/usual care (no contact): 2681 | Prediabetes (participants "had a fasting plasma glucose from 110 to 125 mg/dl - prediabetes or impaired fasting glucose as defined by the World Health Organization within 6 months before the study start date") - physical health | Intervention = Mean age (SD) years: Intervention 1 of 3 (secure email message): 58.3 (11.2) years Intervention 2 of 3 (interactive voice response - telephone message): 58.2 (11.1) years  Control = Mean age (SD) years: Control/usual care (no contact): 58.2 (10.9) years | Intervention 1 of 3 (secure email message): American Indian/Alaska Native: 0.2% Asian: 19% African American: 4% Hispanic: 9% Native Hawaiian or other Pacific Islander: 1% White: 62% Unknown: 5%  Intervention 2 of 3 (interactive voice response - telephone message): American Indian/Alaska Native: 0.6% Asian: 18% African American: 4% Hispanic: 10% Native Hawaiian or other Pacific Islander: 1% White: 62% Unknown: 5%  Control/usual care (no contact): American Indian/Alaska Native: 0.5% Asian: 19% African American: 4% Hispanic: 10% Native Hawaiian or other Pacific Islander: 1% White: 61% Unknown: 5% | The overall uptake rate across intervention arms was 1.9%. Se-cured email message had the highest uptake rate (3.0%), followed by letters and telephone messages (P < .05 for all pairwise comparisons). No participants in the usual-care arm (ie, no outreach)made an appointment with the WCC. For each year of increased age, the estimated odds of the uptake increased by 1.02 (odds ratio [OR] = 1.02; 95% CI, 1.01–1.04). Women were nearly twice as likely to make an appointment at the WCC as men (OR = 1.87;95% CI, 1.40–2.51). |
| Ingrid C Cnossen et al., 2014  Canada | Therefore we developed Head Matters (HM), a multimodal guided self-help exercise program for HNC patients during (C)RT. The aims of the present feasibility study were (1) to explore uptake, adherence, and exercise performance (by exercise levels and exercise categories) of the guided self-help exercise program HM in HNC patients, (2) to explore predictors of exercise performance, and (3) to gain insight into barriers and facilitators to exercise adherence. | Feasibility study | 34 | Intervention - 33 (34 at baseline, 1 patient passed, but only 21 completed the program) | Different types of head and neck cancer patients treated with primary (chemo)radiation or after surgery - physical health: Oral cavity Oropharynx Hypopharynx Larynx | Mean age of the participants was 60 years (range 21-77) | Not specified | Of 41 eligible patients, 34 patients were willing to participate (83% uptake). Of participating patients, 21 patients completed the program (64% adherence rate). The majority of participants (58%) had a moderate to high level of exercise performance. Exercise performance level was not significantly associated with age (P=.50), gender (P=.42), tumor subsite (P=1.00) or tumor stage (P=.20), treatment modality (P=.72), or Head Matters format (Web-based or paper) (P=1.00).  HM appears to be feasible in general, with an uptake percentage >50% (in the present study 83%), with an adherence rate >50% (in the present study 64%), and with a moderate to high performance level >50% of the patients performing exercises in all categories at least once a day on average (in the present study, 58% of the participants).  Adherence and exercise performance level: Of the 33 patients who were interested in performing exercises, 21 patients started and kept up exercising for 6 weeks (64% adherence rate). Of the 33 patients, 14 patients (42%) were performing the exercises at a low level (exercise frequency range of 4-167 during 6 weeks), 10 patients (30%) were exercising at a moderate level (exercise frequency range of 196-332), and 9 patients (27%) were exercising at a high level (exercise frequency range of 372-495). |
| Ingrid V. Bassett, et al., 2016  United States | Our objective was to perform a randomized controlled trial using health system navigators (phone calls, text messages), tailored to resource-limited settings, to evaluate their effect on ART initiation and TB treatment completion among newly diagnosed HIV-infected outpatients in Durban, South Africa. | Randomized controlled trial | HIV-positive = 1899 | Intervention = Pre-intervention = 967 Total outcome eligible (excluding participants who died) = 543 Total primary outcome/alive & outcome eligible = 212/543  Control = Pre-intervention = 932 | HIV (human immunodeficiency virus) - physical health Secondary - TB (tuberculosis) - physical health | Intervention Mean (SD) age only provided for pre-intervention sample = 35 (10) years  Control Mean (SD) age only provided for pre-intervention sample = 35 (10) years | Not clearly defined, but participants were from Durban, South Africa. South African, English-speaking or Zulu-speaking | From August 11, 2010 to January 16, 2013, were screened 6536 people. Of those, 4954 (76%) were eligible and 4903 (99%) enrolled (Fig. 1). The most common reasons for ineligibility included: previous HIV diagnosis (988, 62%),18 years old (277, 18%), and unwilling to share HIV/TB test results (208, 13%). A total of 1899 (39%) enrolled subjects were newly diagnosed with HIV at enrollment of whom 967 (51%) were randomized to the intervention and 932 (49%) to usual care. Overall, 49% were female and mean age was 35 years (SD 10). Baseline demographic characteristics were balanced (Table 1). HIV prevalence ranged from 31% to 52% among enrollment sites. A CD4 count was available for 1659 (87%) HIV-infected participants [772/932 (83%) in usual care and 887/967 (92%) in intervention]. Median CD4 was 192/mL (IQR72–346/uL) and similar across arms: usual care participants had median CD4 200/mL (IQR 72–363/uL) and navigator arm participants had median CD4 186/uL (IQR 72–332/uL). One thousand one hundred forty-six (60%) participants were ART-eligible based on South African guidelines at enrollment, with a median CD4 112/mL (IQR 47–203/uL). Of the 1899 HIV-infected participants, 1685 (89%) had available TB culture data. Three hundred sixty-nine (22%) were TB positive by acid-fast bacillus smear and/or culture. An additional 154 participants were diagnosed with TB outside the study by testing performed on the day of enrollment (included as eligible for primary outcome): 83 chest X-ray, 36 acid-fastbacillus smear, 1 biopsy, 15 ultrasound, 1 clinical indication, 9culture, 1 GeneXpert, and 8 unknown. Two hundred ninety-three (30%) of intervention arm participants and 230 (25%) of usual care participants were coinfected with TB. Eighty-two ofthe HIV/TB coinfected participants were not ART-eligible, representing 4% of the HIV-infected. Forty of these were in theintervention arm and 42 in usual care. |
| Jacqueline N. Casillas et al., 2019  United States | The goal of this study was to compare two innovative, affordable educational interventions – a text-messaging system and a peer navigator program – to traditional, standard-of-care online materials (“Health Links”) on their ability to inform AYAs on topics important to cancer survivorship care. The investigators assessed the ability of these three different approaches to improve AYA survivors’ (1) knowledge regarding late effects risks, and (2) knowledge, attitudes, and self-efficacy to seek survivor-focused care with continuous health insurance coverage. The research team hypothesized that the two intervention groups would have higher scores on late effects knowledge and cancer survivorship care knowledge, attitudes, and behaviors compared to the control group following study completion. These outcomes are important as improvement in AYA cancer survivors’ knowledge of, attitudes towards, and self-efficacy to seek survivor-focused care has the potential to improve long term morbidity and mortality outcomes through increasing early screening for late effects and improving health promotion behaviors. | Randomized controlled trial | 78 | Two intervention arms: Peer navigation arm - n = 25 Text-messaging arm - n = 28  Control = 25 | Cancer (different types) – physical health  Leukemia - n = 44 (56%) Lymphoma (Hodgkin & non-Hodgkin) - n = 44 (56%) Brain/central nervous system - n = 14 (18%) Kidney - n = 1 (1%) Neuroblastoma - n = 4 (5%) Bone/soft-tissue sarcoma - n = 8 (10%) Testicular - n = 1 (1%) Liver - n = 1 (1%) | Intervention = Mean (SD) age of two intervention arms: Peer navigation arm - 21(6) years Text-messaging arm - 21(5) years  Control = 20(5) years | Overall sample size (all intervention arms combined):  Non-Hispanic/Latino white - n = 29 (37%) Black - n = 2 (3%) Asian - n = 4 (5%) Hispanic/Latino - n = 36 (46%) Mixed race/ethnicity - n = 7 (9%) | - Table 1 shows demographics, health statuses, and baseline survey items regarding survivorship identity for the 78 study participants (completed the baseline survey and were randomized to groups). The percentage of non-completers did not differ significantly among the three arms (p=0.21; Fisher exact test). The sample was ethnically diverse. Eighteen percent reported having no current health insurance, and survivors who did not complete the follow-up survey were more likely to lack health insurance than survivors who completed the full study (14% {10/71} for completers versus 57% {4/7} for non-completers, p<0.05). Otherwise, there were no statistically significant differences between study completers (n=71) versus non-completers (n=7) nor between the three arms. Most survivors described their current overall and emotional health status as very good or excellent.  Table 2 summarizes late effects and survivorship care knowledge outcomes for study completers. Knowledge of the term “late effects” was high at baseline. The text-messaging group had a significant increase in late effects knowledge as well as survivorship care knowledge scale scores from pre- to posttest. The text-messaging group also had a significantly greater increase in the overall survivorship care knowledge scale score and two of three subscale items when compared to the control group.  Table 3 summarizes survivorship care attitude and self-efficacy outcomes for study completers. Both intervention groups showed increases on the survivorship care attitude scale compared to the control group, with medium effect sizes of 0.40 for the peer navigation arm and 0.33 for the text-messaging arm.[50] The peer navigation group also had a significant increase in attitude scale scores from pre- to posttest. The peer navigation group had significant increases with medium to large effect sizes for the late effects, survivorship care planning, and health insurance self-efficacy scales in addition to most subscale items; this group also showed significant increases in late effects and health insurance self-efficacy scale scores from pre- to posttest. |
| Jai N Darvall et al., 2020  Canada | Having previously demonstrated the acceptability and utility of the Fitbit Zip pedometer as a remote activity monitoring device in a pilot study [19], we conducted a feasibility randomized controlled trial of a pedometer-based intervention in a cohort of pregnant women with obesity. In particular, we aimed to evaluate the feasibility of self-monitoring of activity levels via the Fitbit Zip pedometer and the additional role of a behavioral intervention in reducing the incidence of excessive GWG. | Randomized controlled trial | 30 | 2 intervention groups: App group: 10 App-coach group: 10  Control 10 | Pregnant women with obesity - physical health | Intervention Mean (SD) age: App group: 30.0 (5.0) years App-coach group: 28.4 (5.8) years  Control Mean (SD) age: 30.2 (5.3) years | Not specified | A total of 30 participants were recruited within a 10-week period, with a dropout rate of 10% (3/30; 2 withdrawals and 1 stillbirth); 27 participants thus completed the study. Mean BMI in all groups was ≥35 kg/m2. Mean (SD) percentage of missing data days were 23.4% (20.6%), 39.5% (32.4%), and 21.1% (16.0%) in control, app group, and app-coach group patients, respectively. Estimated mean baseline activity levels were 14.5 active min/day and 5455 steps/day, with no significant differences found in activity levels between groups, with mean daily step counts in all groups remaining in the sedentary (5000 steps/day) or low activity (5000-7499 steps/day) categories for the entire study duration. There was a mean decrease of 7.8 steps/day for each increase in gestation day over the study period (95% CI 2.91 to 12.69, P=.002). |
| James Balmford et al., 2013  UK | The aims of this article are as follows: (i) To investigate whether uptake of the two auto-mated interventions differs as a function of recruitment source (information seeker versus cold-contacted) and/or recruitment modality(phone or web) (ii) To explore whether offering a choice of interventions results in greater overall uptake than offering only a single option (including where the integrated program is offered as a single option) (iii) To determine whether any of the above differ by sociodemographic or smoking-related factors (iv) To determine whether patterns of uptake are affected by concurrent use of other forms of cessation assistance. | Randomized controlled trial | 3530 | Not provided clearly in study - distinction by recruitment method - not intervention/control.  Cold contacted: 2195, Information Seeker: 1355 | Smoking addiction; Smoking cessation for smokers or recent quitters - both physical and mental health | Not provided clearly in study - distinction by recruitment method - not intervention/control. Cold-contacted: 44.4, Information seeker: 38.4 | Not specified. | From ABSTRACT: Participants were 3530 smokers or recent quitters recruited from two sources; those seeking smoking cessation information, mostly recruited over the phone, and a cold-contacted group recruited from an Internet panel. More participants (60.1%) initially accepted the intervention they had been offered than used it (42.5%). Uptake of each intervention differed substantially by both recruitment source and modality (phone or web). onQ was a little more popular overall, especially in the information seeker sample. Highest overall intervention uptake occurred in the choice condition. A web-based intervention is most attractive if the offer to use is made by web, whereas a phone-based intervention is more likely to be used if the offer is made over the phone. Providing automated interventions on multiple platforms allows for maximal choice and greatest overall use of some form of help...Smoking cessation outcomes from the trial are not a focus of this article and are reported elsewhere[13]; in brief, small effects were found for both interventions, but there was no evidence of a beneficial effect of offering or using both  Sample characteristics and retention: There were no differences in baseline measures across the intervention groups (minimum P-value 0.24), demonstrating that the randomization was effective. However, on all measures the information seeker sample differed significantly from those cold-contacted (Table I), with the pattern of differences confirming that the information seeker sample were more highly motivated to quit. At 1-month follow-up, data were obtained from 88.0% of the sample (n = 3106), mostly by phone (58.1% of completers). Retention did not differ by condition (P = 0.78), but was significantly higher in the information seeker sample (93.1%) than in the cold-contacted sample (84.9%), χ2 (1) = 53.25, P < 0.001. Phone follow-up was significantly more likely in the information seeker sample (88.9%) than in the cold-contacted sample (37.5%), P < 0.001. Of those followed up, 94.4% (n = 2932) were asked about their use of external cessation assistance.  Multivariate prediction of intervention uptake: Predictors of uptake of onQ (independent of any use of QuitCoach), uptake of QuitCoach (independent of onQ usage), uptake of both interventions and uptake of any intervention (i.e. either or both) was examined using logistic regression. The findings, displayed in Table IV, confirm the relationships reported earlier for condition, recruitment source and modality. In addition some demographic differences were found; males and younger smokers were less likely to use QuitCoach, whereas both programs were more likely to be used by the higher educated. As compared with those without a set quit date, those with a date were more likely to use each program, and those already quit were also more likely to use the QuitCoach. As expected, frequency of Internet use was a significant predictor of uptake of QuitCoach, but not of onQ; in fact, likelihood of uptake of onQ decreased as time spent on the Internet per day increased. On the other hand, familiarity with texting was a significant predictor of onQ usage but not of QuitCoach.  Relationship between intervention uptake and use of external cessation assistance: Use of medication was greater in the information seeker sample (P < 0.001), and in both samples, among those who used the interventions offered. All P-values were below P < 0.01, except onQ use in the information seeker sample (P = 0.013). Use of extensive external behavioural help (i.e. other than what was offered) was again significantly greater in the information seeker sample (P < 0.001); however, was not significantly related to intervention uptake. |
| Jamilia R. Sly et al., 2014  Germany | As health professionals begin to increasingly use this mobile technology to communicate with patients, it will be important to assess the initial efficacy of using SMS reminders in health interventions, especially among low-income, racial and ethnic minorities, and older adults (overage 50). It will also be important to understand which contexts and subpopulations are best suited for the use of SMS re-minders. Thus, the objective of this study was to determine the efficacy of SMS as reminders for SC completion and to conduct a power analysis to guide future randomized clinical trials (RCT) | Randomized controlled trial | 24 | Intervention 11  Control 13 | Colonoscopy screening - physical health | Intervention Mean (SD) age (years): 54.55 (5.07)  Control Mean (SD) age (years): 55.92 (5.48) | Overall race/ethnicity (control + intervention): African-American: 52.4% Hispanic/Latino: 47.6% | Only 46.2 % (n=6) of participants in the non-SMS group completed a SC compared to 72.7 % (n=8) of participants in the SMS group (see Table2). There was not a statistically significant difference in the completion rates between the two groups (p=0.19). Based on these results, power calculations indicated that a larger sample size (N=179) would have resulted in statistical significance. |
| Jason Roberge Andrew et al., 2020  United States | To assess whether the availability of a 45-day behavioral health–virtual patient navigation program decreases hospitalization among patients presenting to the ED with a behavioral health crisis or need. | Randomized controlled trial | 637 | Intervention 323  Control 314 | Behavioral health crisis or need - Mental health | Intervention 39.5 (16.8)  Control 39.8 (16.3) | Intervention: African American 32.5%, American Indian or Alaska Native 0.9%, Asian 1.2%, White 52.6%, Multicultural 0.6%, Other 10.5%, Unknown 1.6%  Control: African American 28.7%, American Indian or Alaska Native 0.3%, Asian 1.0%, White 58.0%, Multicultural 0.3%, Other 8.9%, Unknown 2.9% | Among 637 participants, 358 (56.2%) were men, and the mean (SD) age was 39.7 (16.6) years. The conversion rates were 55.1% (178 of 323) in the intervention group vs 63.1% (198 of 314) in the usual care group (odds ratio, 0.74; 95% CI, 0.54-1.02; P = .06). |
| Jason Roberge et al., 2020  United States | This study assesses the availability of a program that offers additional support. Some of the patients who would have defaulted to an admission can be discharged with the extra support. This program is an option for EDs and communities that do not have a dedicated crisis center or, as is the case in many communities with a center, when demand for crisis mental health care outweighs supply. | Randomized controlled trial | 637 | Intervention 323  Control 314 | Behavioral health crisis - mental health | Age, mean (SD), y: 39.5 (16.8) years | Age, mean (SD), y: 39.8 (16.3) years | The percentage of patient encounters with follow-up encounters having a self-harm diagnosis was significantly lower in the intervention group compared with the usual care group (36.8% [119 of 323] vs 45.5% [143 of 314];P= .03). |
| John C et al., 2020  United States | The Living Organ Video Educated Donors program (LOVED) is an 8-week, computer tablet-delivered, distance education, and peer mentorship program. It uses group video chat sessions and education videos with Black kidney transplant eligible ESRD patients to increase knowledge and skills to approach PDs. This unblinded, 2-arm, parallel randomized controlled trial (RCT) investigated the feasibility of LOVED as a LDKT promotion program. Primary feasibility outcomes consisted of program tolerability, defined by program retention rates, fidelity, defined by adherence to program elements and attitudinal and LDKT knowledge. Secondary outcomes included the number of transplant center living donor screenings, PDs completing transplant evaluations and LDKTs at 6 and 12 months. Implications of this study will aid in defining if LOVED is a feasible program prior to a large, multi-center trial. | Randomized controlled trial | 48 | Intervention 24  Control 24 (27 pre-intervention) | Living donor kidney transplants (LDKT) - physical health | Intervention Age, year [mean ± SD]: 50.9 ± 9.2  Control Age, year [mean ± SD]: 47.9 ± 10.0 | 100% Black | Primary feasibility results for LOVED (n = 24) and usual care (n = 24) arms included LOVED program tolerability (i.e., 95.8% retention), program fidelity (i.e., 78.9% video education adherence and 72.1% video chat adherence). LDKT attitudinal and knowledge results favored the LOVED group where a statistically significant effect was reported over 6- months for willingness to approach strangers (estimate ± SE: -1.0 ± .55, F(1, 45.3) = 7.5, P = .009) and self-efficacy to advocate for a LDKT -.81 ± .31, F(1, 45.9) = 15.2, P < .001. Estimates were improved but not statistically significant for willingness to approach family and friends, LDKT knowledge and concerns for living donors (all P’s > .088). |
| Judith Ann Adams et al., 2019  United States | "The purpose of this project was to improve the quality of care for HIV patients by increasing patient engagement in care as evidenced by decreasing the no-show rate. Project goals included initiating text messaging to allow for another patient-centered option for the reminder system, instituting a screening process to identify patients at risk for missing the next appointment, adopting a risk-stratified protocol for increasing patient engagement in care, and rescheduling missed appointments within 24 hours." | Not specified | Not specified or very clear, but some info: Patients with scheduled appointments (n=1642) received the appointment reminder system of their choice: phone call (n=339), text message (n=217), and e-mail (n=67). A total of687 patient charts were reviewed for risk stratification, resulting in 79% (n=544) being assessed as at low risk for missing their next appointment,19% (n=130) were at medium risk, and 2%(n=13) were at high risk. PVP episodes were completed for 67% (n=74) of the medium-risk patients; however, 10 of the patients canceled their appointments and were not rescheduled during the project period. | Not specified | HIV (human immunodeficiency virus) - physical health | Mean/median not provided or clear for intervention group - there also appear to be multiple intervention groups (previsit interventions). However, overall, study participations (HIV clinic patients) were 18 years or older. | Not specified or clear. | Compared to the previous year, similar appointment totals and completion rates of appointments was found after implementation of the interventions. The project resulted in a 3.8% reduction rate (from 30.5% from January-May, 2016 to 26.5 between January-May, 2017) in the overall no-show rate in the first 5 months of implementation (compared to the previous year). |
| Katherine E. Miller et al., 2019  United States | This study intended to evaluate the use and perceptions of CBT-I Coach among Veterans Affairs (VA)- trained CBT-I clinicians | Other: Phenomenological study | 108 | Survey participants: 108 | Insomnia - Mental health | Survey participants: 20-29: 09%, 30-39: 32.4%, 40-49: 12%, 50-59: 34.3%, 60+: 20.4% | Not specified | Overall perceptions of CBT-I Coach were favorable. Fifty percent of clinicians reported using CBT-I Coach, with 98% intending to continue use. The app was perceived to increase sleep diary completion and homework compliance. Clinicians viewed the app as providing accessibility to helpful tools and improving patient engagement. Of those not using the app, 83% endorsed intention to use it. Reasons for nonuse were lack of patient access to smart phones, not being aware of the app, not having time to learn it, and inability to directly access app data. Those who reported using CBT-I Coach had more favorable perceptions across all constructs (p < .01 – p < .001), except relative advantage, com- pared to nonusers. Users perceived it as less complex and more compatible with their practice than nonusers. |
| Kathryn H et al., 2021  United States | We have previously reported a community-based participatory research approach to develop a tablet-based supportive care intervention (Nurse AMIE) and explore initial feasibility and accept-ability [2]. Herein, we report on the results of a pilot study that aimed to further evaluate acceptability, feasibility, patient satisfaction, cost, and initial efficacy of the Nurse AMIE tablet-based supportive care platform. | Other: Partial crossover design | 17 | Intervention 17 | Metastatic breast cancer - physical health | Mean (SD): 60 (11.6) years | White: 16/17 participants  Other: 1/17 participants | Patient satisfaction: Figure1 presents descriptive data regarding whether participants regarded the interventions to be helpful. We collected patient satisfaction ratings on a total of 662 intervention days. Walking was rated as helpful most often (on 83% of days offered). The psychological interventions (e.g., cognitive behavioral therapy instruction and reframing symptoms) were least helpful (51% not helpful,49% helpful).  Intervention cost: The tablets cost $179.99. Cellular data cost$45.00 over 3 months. Non-healthcare provider navigator staff time was $28.77 per hour, with an estimate of 12 h of time per patient. Taken together, the intervention cost estimate is $570.23. These costs do not include the cost of software development for the tablet-based application or healthcare costs for the patients. No additional healthcare visits were incurred as a result of the intervention. |
| Kathy J. Helzlsouer et al., 2016  United States | The aim of this pilot study was to assess the feasibility of an interdisciplinary centralized virtual navigation pro­gram to support treatment completion among newly di­agnosed low-income patients with breast cancer. | Randomized controlled trial | 98 | Intervention Virtual navigation program: 49  Control Web-based information component: 49 | Physical health - Stage 0 to III breast cancer patient | Intervention 52.9 (9.9)  Control 48.7 (10.0) | Navigator group: 34.7% White, 63.3% Black, 2.0% Other Comparison group: 30.6% White, 65.3% Black, 4.1% Other | At the 12-month follow-up, no statistically significant differences were observed in self-rated pain, distress, fa­tigue, mood, and quality-of-life scores. There were no statistically significant differ­ences between the 2 study groups in missed visits (1 or more missed visits: 6.1% for the intervention and 8.2% for the comparison group [P = .85]). Although a higher proportion of patients on the intervention arm had at least 1 ED visit (39% vs 29%) and hospitalization (43% vs 33%), these differences were not statistically signifi­cant (P = .19 and P =.39, respectively; data not shown).  Patients on the intervention arm were contacted an average of 29 times during the 12-month period. The median contact duration was 30 minutes (range, 2-140 minutes). |
| Kelli D et al., 2021  UK | To address this important gap, we conducted a pilot study of an automated, freely available, internet based PCST program, painTRAINER, which has been shown to improve multiple key out-comes among patients with osteoarthritis [17]. Specific-ally, we evaluated the feasibility and acceptability of painTRAINER among patients with SLE and conducted a preliminary assessment of efficacy. We chose to study painTRAINER because pain is a commonly reported symptom among individuals with SLE and because the cognitive and behavioral coping skills included in this intervention can apply to other symptoms and health-related challenges. | Other: Randomized pilot and feasibility study | 60 | 15 post-intervention (initially 30, but only 15 ended up using the intervention)  Control 30 | Systemic Lupus Erythematosus - physical health | Intervention Mean (SD) age - 15 of the intervention users: 51 (14) years  Control Mean (SD) age: 47 (12) years | For 15 of the intervention users: 33% Caucasian  For control: 37% Caucasian | Effect sizes for the painTRAINER group (relative to the wait list group) were small, with changes being greatest for the PROMIS Depression score (d = − 0.32). Among those randomized to the painTRAINER group, 50% accessed the program (“painTRAINER users”). Most of those who did not access the program stated that they did not receive instructions via email. Effect sizes for “painTRAINER users” (relative to wait list) were larger than for the whole painTRAINER group: Pain Catastrophizing d = − 0.60, PROMIS Pain Interference d = − 0.3., PROMIS Depression d = − 0.44, LupusPRO Health-Related Quality of Life d = 0.30. |
| Kerstin Denecke et al., 2018  United States | Concept was presented in the article | Not specified | N/A | N/A | impaired joint function/Endoprosthetic hip surgery (hip TEP) - Physical health | N/A | N/A | Not specified |
| Lara Weinstein et al., 2019  United States | The aim of this study was to examine the outcomes, feasibility, and acceptability of a pilot mammography decision support and navigation intervention (DSNI) for women with SMI living in supportive housing settings. This study was grounded in the preventive health model (PHM) theoretical framework, described in the next section. Our primary hypothesis was: The DSNI approach will increase knowledge, promote favorable attitudes, and decrease decisional conflict relating to screening mammography, as well as support mammography completion. This hypothesis was tested through baseline and follow-up surveys with the participants regarding knowledge, attitudes, and decisional conflict, and measure of the number of completed mammograms. Our secondary hypothesis was that the DNSI will be feasible to implement in supportive housing settings and acceptable to women with SMI. Feasibility will be measured by process measures of the decision support and navigation interventions of the study including percentage of women completing the decision support module, number of navigation attempts, number of completed navigation conversations, participant assessment of the intervention, and navigator reported barriers to navigation, and barriers to mammogram completion. Acceptability will be assessed through semistructured interviews with the participants regarding their experience with the decision support and navigation components of the intervention. | Other: Mixed methods study including quantitative and qualitative components (using participatory methods) | 21 | Intervention = 21 | Serious mental illness (SMI) and mammography screening - both mental and physical health | Age, y, mean (SD): 53.19 (5.62) years | African American: 76%  White: 14% Other/unknown: 10% | Among study participants, 67% received a mammogram. The mammogram DSNI was feasible and acceptable to women with SMI living in supportive housing settings. From baseline to 1-month follow-up, decisional conflict decreased significantly (P= .01). The patient navigation process resulted in 270 attempted contacts (M= 12.86, SD = 10.61) by study staff (phone calls and emails with patient and/or case manager) and 165 navigation conversations (M= 7.86, SD = 4.84). A barrier to navigation was phone communication, with in-person navigation being more successful...Conclusions: The process and outcomes evaluation support the feasibility and acceptability of the mammography DSNI. |
| Lena Sanci Sylvia et al., 2019  Canada | The primary objective of this study was to assess the impact of Link on young people’s PA compared with usual help-seeking strategies immediately post intervention. Secondary objectives were to compare the intervention and control participants on measures of PA 1 and 3 months after using Link and on negative affect (NA), psychological distress, barriers to seeking help, and help-seeking intentions at 1 and 3 months postintervention. | Randomized controlled trial | 413 | Intervention 205  Control 208 | Various mental health conditions - mental health | Characteristics between young people who withdrew and those who completed the study were similar, with the mean age of participants at baseline being 20.7 (SD 2.3) and 21.3 (SD 2.1) in the intervention and control arms, respectively (0 missing responses in both arms).  Control Characteristics between young people who withdrew and those who completed the study were similar, with the mean age of participants at baseline being 20.7 (SD 2.3) and 21.3 (SD 2.1) in the intervention and control arms, respectively (0 missing responses in both arms). | Only provided the following: Intervention: Aboriginal or Torres Strait Islander = 2.0%  Control: Aboriginal or Torres Strait Islander = 3.0% | In total 413 young people were recruited to the trial (intervention, n=205; control, n=208) and 78% (160/205) of those randomized to the intervention arm visited the Link website. There was no evidence to support a difference between the intervention and control arms on the primary outcome, with PA increasing equally by approximately 30% between baseline and 3 months in both arms. NA decreased for the intervention arm compared with the control arm with a difference of 1.4 (95% CI 0.2-2.5) points immediately after the intervention and 2.6 (95% CI 1.1-4.1) at 1 month. K10 scores were unchanged and remained high in both arms. No changes were found on the BASH or GHSQ; however, participants in the intervention arm appeared more satisfied with their help-seeking process and outcomes at 1 and 3 months postintervention. |
| Lorna Cook et al., 2019  Canada | The primary objective of this study was to test whether guided Web-based RFCBT (i-RFCBT) would prevent the incidence of major depression relative to usual care in UK university students. The secondary objective was to test the feasibility and estimated effect sizes of unguided i-RFCBT. | Randomized controlled trial | 235 | Intervention Guided i-RFCBT: 50, Unguided I-RFCBT: 48  Control Waitlist control: 77 | Ruminating thoughts/depression - Mental health | Intervention Guided i-RFCBT: 20.43 (1.65), Unguided I-RFCBT: 20.53 (1.30)  Control Waitlist control: 20.27 (1.55) | Guided i-RFCBT: White: 91%, Unguided I-RFCBT: 88%, Waitlist control: 91% | Participants were randomized to guided i-RFCBT (n=82), unguided i-RFCBT (n=76), or usual care (n=77). Guided i-RFCBT reduced the risk of depression by 34% relative to usual care (hazard ratio [HR] 0.66, 95% CI 0.35 to 1.25; P=.20). Participants with higher levels of baseline stress benefited most from the intervention (HR 0.43, 95% CI 0.21 to 0.87; P=.02). Of the 6 modules, guided participants completed a mean of 3.46 modules (SD 2.25), with 46% (38/82) being compliant (completing ≥4 modules). Similar effect sizes and compliance rates were found for unguided i-RFCBT. |
| Lübomira Spassova et al., 2016  UK | In order to assess the efficacy of the CAPSYS approach concerning the reduction of stroke-related risk factors as well as the usability of the system, a dedicated study was designed and carried out, the results of which are presented in this article. | Randomized controlled trial | 94 | Intervention = 48 (32 participants completed intervention/final questionnaires - intention to treat analysis was used)  Control = 46 | Stroke or cerebro-cardiovascular (CVA) risk factors - physical health | Intervention Mean age [years](±SD): 60.7 (±11.3)  Control Mean age [years](±SD): 59.6 (±12.1) | Not provided. | The statistical evaluation of primary measures revealed significant decreases of systolic blood pressure (mean of the differences=–9 mmHg; p=0.03; 95 % CI=[–17.29, –0.71]), LDL (pseudo-median of the differences=–7.9mg/dl; p=0.04; 95 % CI=[–18.5, –0.5]) and triglyceride values (pseudo-median of the differences=–12.5 mg/dl;p=0.04; 95 % CI=[–26, –0.5]) in the intervention group, while no such changes could be observed in the control group. |
| Lynn F. Reinke et al., 2011  United States | The purpose of this exploratory study was to test the feasibility and preliminary efficacy of an interactive Web-based seminar to equip patients diagnosed with COPD with the knowledge and skills to engage in conversations about their end-of-life care wishes. Results will be used as a foundation for larger studies on the effectiveness of using communication technologies to facilitate end-of-life communication-coaching for patients who are geographically distributed. | Feasibility study | 7 | Intervention 7 | Patients with severe to very severe chronic obstructive pulmonary disease (COPD) (GOLD Stage III-IV) + end-of-life care - physical health (may include mental health due to end-of-life aspect) | 64 +/- 4 years | Caucasian: 86% African American: 14% | Prewebinar, 6 of the 7 patients had completed advanced care planning forms but only half had shared these with their clinicians. Most patients felt confident about discussing end-of-life preferences. At 3 months, all participants had taken further action on end-of-life planning. Five felt the webinar was an acceptable option if unable to participate in person. |
| Margarita Elkjaer et al., 2010  UK | The aims of this randomised control trial were first to investigate whether a self-administered, web-based e-health treatment programme during1 year was feasible for Danish and English speaking patients with ulcerative colitis on 5-ASA therapy, second to determine whether this e-health approach could improve patient compliance, knowledge, QoL, disease outcomes, and safety and third to investigate whether the e-health approach could decrease healthcare costs primarily in Denmark. | Randomized controlled trial | Denmark & Ireland: 333 | Intervention Denmark: 105 at baseline visit, but only 89 completed the trial to 12 months. Ireland: 51 at baseline visit, but only 40 completed the trial to 12 months.  Control Denmark: 106 (at baseline visit) Ireland: 41 (at baseline visit) | Mild/moderate ulcerative colitis and 5-aminosalicylate acid (ASA) treatment - physical health | Not specified clearly, but inclusion criteria was for patients aged 18 to 69 years. | Not provided. | 88% of the web patients preferred using the new approach. Adherence to 4 weeks of acute treatment was increased by 31% in Denmark and 44% in Ireland compared to the control groups. In Denmark IBD knowledge and QoL were significantly improved in web patients. Median relapse duration was 18 days (95% CI10 to 21) in the web versus 77 days (95% CI 46 to 108)in the control group. The number of acute and routine visits to the outpatient clinic was lower in the web than in the control group, resulting in a saving of 189 euro/patient/year. |
| Maricianah Atieno Onono et al., 2019  UK | Objective was to evaluate the impact of this intervention on women’s adherence to recommended antenatal (ANC) and postnatal care (PNC) regimes and facility birth | Cohort study | 1166 | Intervention 349  Control 817 | Pregnancy - physical health | Intervention 24.9 (5.5)  Control 24.7 (5.8) | Women from Kenya | Cases (women who received the intervention) had five times higher odds of having four or more ANC visits (aOR=4.7, 95% CI 3.20 to 7.09), three times higher odds of taking between 30 and 60 min to reach a health facility for delivery (aOR=3.14, 95% CI 2.37 to 4.15) and four times higher odds of undergoing at least four PNC visits (aOR=4.10, 95% CI 3.11 to 5.36). |
| Marie Ferrua et al., 2021  Germany | The first objective of this paper was to provide a comprehensive description of the CAPRI-COVID intervention; the second objective was to provide feedback on the usefulness of this platform for cancer patients by thoroughly assessing the tracking indicators, analyzing NNs activities, and assessing the experience of patients. | Non-randomized experimental study | 129 (130 originally, 1 consent withdrawal) | Not specified | Multiple types of cancers - physical health Breast Head and neck Hematological Gynecological Digestive Renal Skin Lung Other | Intervention Mean age (SD) for CAPRI intervention: 55 (16) years  Two intervention arms (telephone and CAPRI app) - no control | Not specified. | All 130 (could be an error, actually 129) patients (median age: 59 years; 59.2% female) were monitored during the study period. There were no deaths or admissions to the intensive care unit attributable to COVID-19; 7.8% of patients were hospitalized (excluding scheduled hospitalization), and 17.1% were admitted to the emergency department at least once during the monitoring period. NNs carried out 1412 regular monitoring calls (average of 10.9 calls per patient), while 55% of the patients downloaded the CAPRI App.  Demographic Data: As a part of the CAPRI-COVID intervention, 130 patients with COVID-19 were monitored; findings from 129 are presented in Table1(one patient requested the withdrawal of their consent for the use of personal data). Sixty-two patients completed the survey, with a response rate of 48.1%. Fifty-five percent of patients were enrolled after hospitalization forCOVID-19 and 45.0% after RT-PCR test performed in the out patient department (Fig.1). Among all patients, 19.4%did not show symptoms of COVID-19. The median time from symptom onset to the start of monitoring was 12 (1–66) days, with a mean duration of 14.7 (5–39) days.  Tracking indicators: There were no deaths or admissions to the intensive care unit attributable to COVID-19. Ten (7.8%) patients were hospitalized (excluding scheduled hospitalization), and 23 (17.1%)were admitted to the emergency department at least once during monitoring. Among hospitalized patients, 50% had already been hospitalized for COVID-19.  NN activities: NNs conducted 1412 regular telemonitoring calls to assess the evolution of COVID-19-related symptoms, with an average of10.9 calls per patient. For patients using the CAPRI App, the number of calls decreased to 6.2 calls per patient compared to13.9 for those monitored via phone. NNs received 35 incoming calls from 22 patients (13 patients monitored via phone and 9 via CAPRI App). Thus, 1447 interventions were analyzed....The CAPRI-COVID intervention involved an average of two full-time NNs, 7 days a week, over a total duration of 70 days.  CAPRI app: Only 114 (88.4%) patients had a valid e-mail address (required to access the CAPRI App) at inclusion. Among them,71 (62.3%) downloaded the CAPRI App, and 50 (43.9 %)completed the tracking data (average 16 times, 1.1 times per day) (Fig.1). Three patients were not compliant with the monitoring via the CAPRI App and switched to phone monitoring. The rate of responses to the survey indicates that patients using the CAPRI App were overall satisfied and did not con-sider the procedure too constraining (n=32)(Fig.2). The main reason given by patients for not downloading the CAPRI App was their lack of familiarity with computers/smartphones (cf.Table2). Patients who used the CAPRI App infrequently preferred regular phone contact with NNs (n=7). |
| Martin Angelo et al., 2021  United States | The overall goal of this quality improvement project was to improve CRC screening rates among adult Hispanic patients using a mail-in FIT and an unlicensed, bilingual PN for support and education using secure SMS. The project-specific aims were as follows:  - Improve CRC screening rates among Hispanic adults aged 50–75 years by 25% after three months of project implementation.  - Have a 50% participant response rate to SMS received by a PN regarding the post-CRC screening questionnaire. | Other: Quality improvement project using a retrospective chart review | 91 | Intervention Pre-project implementation: 36 Post-project implementation: 55 | Colorectal Cancer Screening - physical health | Mean/median age not provided - only age range: 50-75 years for all participants. | Participant eligibility criteria included being Hispanic. | Based on the retrospective chart review, 36 patients who self-identified as Hispanic and were aged 50–75 years were randomly selected. Of the 36 patients in the chart review, only 11 had completed a method of CRC screening. A total of 55 patients were then invited to participate in the project. Of the 55 patients invited, 36 participants completed the FIT (65% recruitment rate). Of the 36 participants who completed the FIT, only two did not respond to the post-FIT screening questionnaire (95% retention rate). Two participants opted out of the questionnaire because of discomfort in answering questions. All participants self-identified as Hispanic and were aged an average of 58 years. Language preference was Spanish for most participants, most completed some form of formal education, and all reported incomes above the poverty level. Table 1 provides a detailed description of the demographics of all participants.  The first aim of this project focused on improving the FIT screening rate in Hispanic adults at WCMFC aged 50–75 years by 25% after three months of project implementation. The screening rate increased from pre– (n = 11, 31%) to post–project implementation (n = 36, 66%) (p < 0.001), which exceeded the goal of a 25% improvement in screening rate.  The second aim assessed whether the secure SMS sent by the PN had an influence on participants’ decisions to complete the FIT. Of the 36 eligible patients who completed the FIT screening, 34 responded. Among the respondents, 23 reported that having a PN reach out via telephone influenced their decision to complete the at-home FIT. The majority of participants were in favor of having a Spanish-speaking PN reach out to them because Spanish was the preferred language of 41 of the participants. Of note, 24 were dissatisfied with receiving SMS with a link to a Spanish-translated CRC educational video. Of those who provided a non-positive response, verbal feedback was provided. Overall, 31 participants felt that completing the FIT was easy. The majority of the participants (n = 29) reported that they are likely to complete the test again the following year. Table 2 provides a summary of the questionnaire results and qualitative responses of the participants. |
| Martina Nitsch et al., 2016  Canada | The aim of this study was to explore engagement and corresponding usability issues of the Healthy Body Image Program—a guided online intervention for individuals with body image concerns or eating disorders. The secondary aim was to demonstrate the value of usability research in order to investigate engagement. | Other: iterative usability study based on a mixed-methods approach | 9 | Intervention - Based on an iterative usability study design approach, we aimed to conduct tests in two rounds with no more than 5 participants per round, since usability testing with 5 users reveals 85% of usability problems and more than 5 users would produce repetitive information | Individuals with body image concerns or eating disorders- Mental and Physical health | Only range provided - 18-25 | Not specified | Participants were satisfied with the overall usability of the program. The average usability score was 77.5/100 for the first test round and improved to 83.1/100 after applying modifications for the second iteration.  The majority of participants liked the layout of the program and described it as “friendly,” “youthful,” and “pretty,” similarly emphasizing the “nice colors and graphics” as well as the “easy format.” A few users mentioned that the interface seemed familiar to them and that it looked like a start-up, which was interpreted positively by some, and negatively by others, as it seemed to “be just another algorithm.” |
| Marybeth Allen et al., 2008  United States | Not provided exclusively, but some indication: "To describe development of an Internet-based health coaching intervention."  "The focus of the Internet-portal-based intervention was to improve the care of adult primary care patients with chronic pain, depression, or difficulty walking. The specific aim of the intervention was to improve patient-reported quality of life, diagnosis and treatment of the three conditions, patient clinician communication, and satisfaction with primary care. Consistent with social cognitive theory (Bandura, 1977), the aim was to achieve these objectives through empowering patients to work collaboratively with their primary care doctors and encouraging them to set goals for their healthcare visits. The intervention was designed to promote patient self-efficacy specifically in the domain of communication with providers. Patients were guided to organize their thoughts and find constructive ways to communicate their health priorities through nurse e-coach e-mailed advice and tools offered entirely through the secure PatientSite Internet portal."  "An important aim of the study was to improve detection of the three chronic conditions. It was expected that, by informing patients of their screened condition, coupled with motivational e-coaching messages, patients would be more likely to discuss their condition with their clinician during their upcoming visit." | Randomized controlled trial | 121 | Intervention 121 | Chronic (musculoskeletal) pain Mobility difficulty Depression Physical and mental health | Mean/median not specified - age range and % provided. Participants (patients) were aged 20 years and above.  20-39 years - 17.4% (n = 21) 40-49 years - 22.3% (27) 50-59 years - 35.5% (43) 60+ years - 24.8% (30) | White - 90.8% (n = 109) Black - 5.8% (7) Other - 3.3% (4) Hispanic ethnicity - 1.7% (2) | An earlier study of PatientSite users showed that, in general, they are younger (though 7% are at least age 65 years or older) and healthier than nonusers and are more likely to be White (Weingart, Rind, Tofias, & Sands, 2006). The 121 intervention participants ranged in age from 22 to 82 years, with 60% aged 50 years and older. More than half were women (59%), most were White (91%), and 69% had attended 4 or more years of college. |
| Meelim Kim et al., 2020  Canada | The goal of this study was to test a novel approach to losing weight and maintaining the new weight after participation in an intensive and comprehensive human coaching program based on CBT modules via digital tools, such as the Noom Coach app and InBody Dial. | Randomized controlled trial | 70 | Intervention Digital CBT: 44  Control = 25 | Obesity - Physical & mental health | Intervention Digital CBT: 23.3 (3.5)  Control 21.0 (2.7) | Not specified - University students in Seoul, South Korea | Mean weight loss at 8 weeks in the digital CBT group was significantly higher than in the control group (– 3.1%, SD 4.5, vs –0.7%, SD 3.4, P=.04). Additionally, the proportion of subjects who attained conventional 5% weight loss from baseline in the digital CBT group was significantly higher than in the control group at 8 weeks (32% [12/38] vs 4% [1/21], P=.02) but not at 24 weeks. |
| Meg Simione Laura et al., 2021  UK | The purpose of this study is to examine behavior (i.e, diet, screen time, physical activity), psychosocial (i.e., anxiety), and WIC program enrollment changes from the first to third trimester for low-income women at high risk for obesity participating in the systems-oriented First 1000Days Program in the greater Boston area. We hypothesized that program participation would lead to improvements in behavioral and psycho-social risk factors and promote the use of social sup-port services during pregnancy. | Other: Secondary analysis using a quasi-experimental pre-post design | 264 | Intervention = 264 | Obesity and obesity risk factors among mother-infant pairs - physical health | Years, mean (SD): 30.8 (5.51) | White, Non‑Hispanic: 44.7% Hispanic or Latino: 31.1% Black, Non‑Hispanic: 4.5% Other: 19.7% | Women completed surveys at their initial and third trimester prenatal visits (n=264). Mean age (SD) was 30.2 (5.51) years and 75% had an annual household income of <$50,000. Mean pre‑pregnancy body mass index (BMI) was 27.7kg/m2 and 64% started pregnancy with a BMI≥25kg/m2. In multivariable adjusted models, we observed decreases in intake of sugary‑drinks (−0.95 servings/day; 95% CI: −1.86, −0.03) and in screen time (−0.21h/day; 95% CI: −0.40, −0.01), and an increase in physical activity (0.88days/week; 95% CI: 0.52, 1.23) from the first to third trimester. We also observed a decrease in pregnancy‑related anxiety score (−1.06units; 95% CI: −1.32, −0.79) and higher odds of enrollment in Women, Infant, and Children (WIC) program (OR: 2.58; 95% CI: 1.96, 3.41). |
| Michelle J. Naughton et al., 2021  Netherlands | To evaluate the feasibility of implementing systematic patient symptom monitoring during treatment using a smartphone. | Feasibility study | 313 | Intervention = Breast: 193, Ovarian: 70, N: Endometrial: 50 | Ovarian, endometrial, and breast cancer - Physical health | Mean (range) Breast: 55.2 (26-82), Ovarian: 62.9 (35-87), Endometrial: 63.1 (43-87) | 85% were non-Hispanic white | At program entry, 74.1% of all patients were already enrolled in MyChart. Patients not enrolled were asked to enroll, with refusals ranging between 37 and 50% of the non-enrolled patients. Reasons for refusing were that they did not have reliable access to the internet or computers, preferred to call or talk to health professionals in person, or simply were not interested in using MyChart. Approximately 55% of patients over age 65 refused enrollment in MyChart, primarily among the ovarian and endometrial patient groups.  iPhones were provided to 42 (13.4%) patients across all can- cer types. Demographic characteristics of the patients who received iPhones were compared with those who already had a smartphone, with patients receiving iPhones having incomes below $50,000/year (p = 0.03) and an educational level of high school or less (p < 0.0001). Program staff had few difficulties training patients to operate the phones correctly or in patients’ adherence to completing surveys after receiving the iPhones.  Adherence averaged between 75 and 77% overall with responses varying by cancer type, as well as the month of assessment. Patients were censored at the time of their formal withdrawal from the program or death, so that the monthly percentages only include active patients who completed the surveys at each time point.  Alert values for PHQ-9 scores ≥ 10 occurred in roughly one-third of the gynecologic oncology patients, with 3% expressing suicidal ideation. The majority of these patients were already receiving behavioral health services, with those not under care referred to behavioral health services in their areas |
| Min-Kyung Lee et al., 2021  Canada | This study aims to evaluate the usefulness of a novel user utility score (UUS) as a tool to measure patient engagement by using a mobile health application for diabetes management. | Other: Secondary Analysis of a Randomized Controlled Trial  conducted a subanalysis of results from a 12-month randomized controlled trial | 72 | Intervention = UUS: 0-4 - 38  Control = USS: 5-8 - 34 | Type 2 Diabetes - physical health | Intervention UUS: 0-4 - 50.58 (8.52)  Control USS: 5-8 - 52.38 (7.13) | Korean | There was a significant between-group difference in glycated hemoglobin test (HbA1c) levels for the 12-months study period (P=.011). The HbA1c decrement at 12 months in the UUS:5-8 group was greater than that of the UUS:0-4 group [–0.92 (SD 1.24%) vs –0.33 (SD 0.80%); P=.049]. After adjusting for confounding factors, UUS was significantly associated with changes in HbA1c at 3, 6, and 12 months; the regression coefficients were –0.113 (SD 0.040; P=.006), –0.143 (SD 0.045; P=.002), and – 0.136 (SD 0.052; P=.011), respectively. |
| Monika Jurkeviciute et al., 2020  Canada | The objective of our study is to identify contextual factors that determine similarities and differences in the value of an eHealth intervention between two contexts. We also aim to reflect on and contribute to the discussion about the specification, assessment, and relativity of the “value” concept in the evaluation of eHealth interventions. | Other: Observational study | Patients in Italy (n=53), Patients in Sweden (n=54) | Patients in Italy (n=53), Patients in Sweden (n=54) | Cognitive impairments - Mental health | Italy: 77.6 (5.3) Sweden: 74.8 (5.9) | Not specified | The value of an eHealth intervention applied to similar types of populations but differed in different contexts. In Sweden, patients improved cognitive performance (MMSE mean 0.85, SD 1.62, P<.001), reduced anxiety (EQ-5D-5L mean 0.16, SD 0.54, P=.046), perceived their health better (EQ-5D-5L VAS scale mean 2.6, SD 9.7, P=.035), and both patients and health care professionals were satisfied with the care. However, the Swedish service model demonstrated an increased cost, higher workload for health care professionals, and the intervention was not cost-efficient. In Italy, the patients were satisfied with the care received, and the health care professionals felt empowered and had an acceptable workload. Moreover, the intervention was cost-effective. However, clinical efficacy and quality of life improvements have not been observed. |
| Nadim Mahmud et al., 2019  United States | To pilot a text message navigation program to improve colonoscopy adherence | Other: prospective study | 71 | Intervention = 21  Control 50 | Various conditions that require and/or patients who colonoscopy screening - Physical health | Intervention Median (IQR): 56 (48,63)  Control Median (IQR): 60.5 (53,67) | Control: White 40%, Black 52%, Asian 8%  Intervention: White 43%, Black 57%, Asian 0% | The arms had similar demographics and comorbidities. Intervention patients had higher colonoscopy appointment adherence (90% vs. 62%, p = 0.049). There were no significant differences in preparation quality or procedure completeness. Poststudy surveys indicated high patient satisfaction and perceived usefulness of the program. |
| Natalie Liling et al., 2021  UK | The aim of this project was to mitigate the negative impact of isolation on hospital stay of patients with COVID-19 by addressing their informational and psycho-social needs, empower and reassure them with accurate information and integrate the humanities into clinical care. | Other: Quality Improvement (Project) Report | 83 | Intervention 83 | Suspicion or confirmed cases of COVID-19 and psychosocial needs of patients - both physical and mental health | Median age (IQR): 40 (12–93) years | Race/ethnic background not specified, but participants were admitted to the Singapore General Hospital (may or may not be Singapore locals). | Six hundred and thirty patients were isolated for suspicions of COVID-19 in SGH from 24 January to 19 March, of whom 24 had confirmed COVID-19 infection. Three hundred and thirty-nine (53.8%) of them were female. The median age of all patients was 40 (12–93), with males a decade older (37 (15–93) years vs 47 (12–93) years). Chinese accounted for 413 (65.5%) of them, Malays were 59 (9.3%) and other ethnicities made up 158 (25%). Confirmed cases had a median length of stay of 15 days in isolation while suspect cases had a median length of stay of 2 days. |
| Natalie Stein & Kevin Brooks, 2017  Canada | Because of the shortcomings in traditional health care delivery channels to help patients achieve healthy lifestyle changes for lowering T2D risk, and the potential for mobile technologies to provide effective and compassionate interventions, there is a role for conversational AI to provide highly scalable health coaching to effect positive change in behaviors known to lowerT2D risk. This study’s objectives were to (1) investigate conversational AI use and relationships with weight loss and meal healthiness, and (2) investigate user engagement and acceptability of the HCAI. We hypothesized that AI users would lose weight and improve meal healthiness. | Other: Retrospective longitudinal observational study | 70 | Intervention = 70 | Overweight and obese (body mass index ≥25) (at risk of Type 2 diabetes) - physical health | Mean (SD): 46.9 (1.89) years | Not specified. | Data were analyzed for participants (N=70) who met engagement standards set forth by the Centers for Disease Control and Prevention criteria for Diabetes Prevention Program, a clinically proven weight loss program focused on preventing diabetes. Weight loss (standard error of the mean) was 2.38% (0.69%) of baseline weight. The average duration of app use was 15 (SD1.0) weeks, and users averaged 103 sessions each. Predictors of weight loss included duration of AI use, number of counseling sessions, and number of meals logged. Percentage of healthy meals increased by 31%. The in-app user trust survey had a 100%response rate and positive results, with a satisfaction score of 87 out of 100 and net promoter score of 47.  User Statistics: Participant baseline characteristics are presented in Table 2.Users were 74.5% (35/47) female with average age 47 years. Baseline weight was 98.0 kg (SD 3.16) and BMI was 37.0 kg/m2(SD 1.40). Standard error of the mean (SEM) is included. |
| Natasha M Simske et al., 2019  United States | The aim of the present study is to evaluate patient satisfaction with TSN services and the impact of these services on patient perceptions about recovery. It is hypothesized that patients interacting with TSN will have greater satisfaction, optimism, and self-efficacy, indicating a constructive impact of this program and benefits of future widespread application. | Cohort study | 485 | Intervention 211  Control 1 (no TSN): 135  Control 2 (prior to TSN existence): 139 | Musculoskeletal injuries and trauma (physical, social, and psychological) - both physical and mental health | Intervention Mean (SD): 43.0 (17.1) years  Control Mean (SD) age:  Control 1 (no TSN): 46.3 (19.9) years  Control 2 (prior to TSN existence): 4.3 (17.6) years | Not specified. | On a Likert scale from 0 to 5, patients were highly satisfied (mean 4.24), with no differences based on TSN exposure. Patients exposed to TSN programming reported greater perceived likelihood of recovery: mean 3.73 vs 3.41 vs 3.38, Group 1 vs Group 2 vs Group 3 (P=.05) and regarding return to daily activities: 3.69 vs 3.49 vs 3.10,P=.003. Fifty-three percent of Group 1 patients exposed to TSN programming utilized peer relationships and 42% read the educational materials provided. Support groups were also popular, with 26% of patients attending at least 1 session. Patients who recalled utilization of TSN services were overall highly satisfied with these services, mean 4.42....Conclusion: Patients were overall highly satisfied with their hospital stay, with those exposed to TSN services reporting greater perceived likelihood of recovery and return to daily activities. Development of nontraditional services, including peer visitation and support groups, appears to enhance expectations about recovery.  3.1. Response rate: Four hundred eighty-five surveys were sent to patients, with 160responses for an overall response rate of 32.9%. Group 1 had the highest response rate, 35.5%, with 75 of 211 sent surveys being returned. Group 2 had the second highest, 34.1%, with 46 of 135patients returning surveys. Group 3 had the lowest response rate,28.1%, with only 39 of 139 patients responding to mailed surveys. Response rates were no different between groups(P=.57).  3.2. Demographics: The mean age of patients who received surveys was 43.0 years(SD=18.1), and 65.8% were male. The most common mechanism of injury was a motor-vehicle collision (MVC), (n=155,32%), followed by falls (n=154, 31.8%). The most prevalent injuries were to the tibia or ankle (n=148, 30.5%) and to the femur (n=129, 26.6%). Overall, patients were well matched. Survey groups were only dissimilar in terms of motorcycle collisions (MCCs), pedestrian collisions, and upper extremity injuries. Group 1, the TSN-exposed cohort, had substantially more operative upper extremity fractures (19%,P=.0011). All other demographic and injury variables did not reach statistical significance (Table 1). |
| Nichole Kang et al., 2020  United States | To collect data from feedback surveys and case records from clients and Navigators of SELPHI. | Qualitative research | 59 | 59 | Pregnancy/parenting - Physical health | 19.84 (1.61) | Hispanic: 15.3%, Non-hispanic: 81.4%, refused: 3.4% | SELPHI used ICT to support program implementation. Navigators contacted clients via phone, text, email, video call, and social media messaging and recorded their contact attempts in the database. SELPHI enrolled 59 clients; Navi- gators made a total of 799 contact attempts using ICT and an additional 150 in-person contacts. Most SELPHI clients engaged in at least one in-person meeting with their Navi- gator, with the average client meeting face to face between three and four times.  Within the 6-month implementation period, stakeholders learned several lessons about the role that technology can and cannot play in case management with expectant and parenting youth and found both successes and challenges with using ICT for these services.  Having multiple contact methods for each client avoids gaps in case management services. In the Navigator Feed- back Survey, three of the four Navigators identified lapsed phone service as a communication barrier for their clients and reported that this barrier was mitigated by access to multiple communication methods, |
| Olga Solonowicz et al., 2022  United States | We conducted a randomized controlled quality improvement study to formally determine whether a bowel preparation-specific, text message program containing timed-release instructions would be associated with improved outcomes for patients scheduled for colonoscopy. Our primary aim was to evaluate whether the digital program was associated with a reduction in no-show rates compared with patients getting standard navigation (paper instructions and nursing calls). Our secondary aim was to evaluate the effect of digital navigation on a combined metric of no-shows with same-day cancellations no-shows with cancellations within 7 days of the scheduled procedure, bowel preparation quality, and patient satisfaction. | Randomized controlled trial | 1625 | Intervention 833  Control 792 | Bowel Preparation Quality for Patients Undergoing Colonoscopy - physical health | Intervention Age, median (IQR): 57 (50-65) years  Control Age, median (IQR): 57 (50-66) years | Not specified | A total of 1625 patients were randomized (SMS=833,control=792). No-show rates were significantly lower in the SMS group compared with the control group (8% vs. 14%;P<0.0001).Similar results were found for no-show/same-day cancellations (10%vs. 16%;P=0.0003), and no-show/cancellations within 7 days (18%vs. 26%;P=0.0008). There was no difference in adequate bowel preparation for all colonoscopies between the groups (89% vs. 87%;P=0.47). However, rates of adequate bowel preparation for screening/surveillance colonoscopies were significantly higher in SMS versus control groups (93% vs. 88%;P=0.04).  Demographic info: After exclusion of unreachable patients, we included1625 eligible patients who were scheduled for a colonoscopy and enrolled in the study over the 3-month time period. After randomization, 792 patients were enrolled in the control group and 833 patients in the intervention group(Fig. 2). Patient characteristics including baseline comorbidities for both groups are compared in Table 1. The median age of the overall cohort was 57 years. Most patients(97%) reported English as their preferred language. There was no significant difference in the history of patient no-shows or the indication for the scheduled and/or completed colonoscopy between the 2 groups.  Primary and secondary outcomes: In total, 172 of 1625 (11%) scheduled patients did not show up on the day of their procedure. There was a significant reduction in no-show rates in the intervention group compared with the control group (8%, n=63/833 vs. 14%,n=109/792;P<0.0001; Table 2). Similar results were found in the secondary outcomes of combined no-show/same-day cancellations, and no-show/cancellations within 7 days of the scheduled procedure. A total of 216 of 1625 (13%)patients had a combined no-show/same-day cancellation. Patients in the text message group were significantly less likely to no-show/same-day cancel than controls (10%,n=86/833 vs. 16%, n=130/792;P=0.0003). In addition,356 of 1625 (22%) patients had a no-show/cancellation within 7 days, and the intervention group showed significant reduction compared with the control group (18%, n=154/833 vs. 26%, n=202/792;P=0.0008)...The effect of the text message intervention remained significant across nearly all subgroups (Fig. 3). However, the intervention was not significantly associated with a reduction in no-shows in patients over the age of 65, retirees, patients with a prior history of no-shows, and patients receiving Miralax as their bowel preparation agent. On a multivariable analysis adjusting for plausible biological and demographic confounders, the intervention was significantly associated with the reduction of no-shows [odds ratio (OR), 0.51; 95% confidence interval (CI), 0.35-0.74;P=0.0004] Additional variables that were independently associated with no-shows included single marital status (OR,1.72; 95% CI, 1.15-2.59;P=0.009), Medicaid insurance(OR, 2.09; 95% CI, 1.33-3.29;P=0.001), history of no-shows(OR, 1.66; 95% CI, 1.09-2.50;P=0.02), and patients with a prior colonoscopy (OR, 0.08; 95% CI, 0.05-0.12;P<0.0001; Table 3). |
| Owen Katalinic et al., 2013  UK | Trial objectives were to improve access to services, self-management of health conditions and health education, and to reduce social isolation. | Non-randomized experimental study | 102 | iPad: Paediatric palliative care service: 14  Stroke service: 39 Brain Injury rehab service: 21  Intel health guide: Cardiac coaching service: 28 | Paediatric palatine care services, stroke services, brain injury rehab services, cardiac coaching service - Physical health | iPad: Paediatric palliative care service: 6 (6) Stroke service: 67(14) Brain Injury rehab service: 42(17)  Intel health guide: Cardiac coaching service: 66(11) | Not specified | The main findings were:  1. Telehealth can play a useful role in improving accessto services, particularly for those who live in ruralareas.  2. Detailed planning and high-level support for telehealth is essential in establishing a framework for telehealth.  3. Rapid resolution of problems is essential in maintaining clinician engagement.  4. Both patients and clinicians readily accept and learn how to use new technologies, particularly where using them saves significant amounts of time.  5. Usability and ease of use are critical in ensuring theacceptance of the technologies.  6. The lack of broadband Internet in some regional and rural areas rules out the use of telehealth in those areas.  7. Internet bandwidth and latency plays a critical role in the quality and experience of the video conference.  8. Latency and quality of video conferencing over mobile broadband networks is highly variable, depending on the local signal strength.  9. Technical problems that affect the Wide Area Network can have serious effects on the delivery of home-based telehealth services.  10. Initial concerns that equipment would be lost or stolen from patient’s homes have proved unfounded. To date, there have been no losses of home telehealth equipment.  11. Age does not appear to be a barrier to using home telehealth technologies and video conferencing technology.  12. The use of iPads for clinical therapeutic purposes hassignificant potential and is well accepted by cliniciansand patients. This will continue to be explored. |
| P. W. Colson et al., 2020  United States | To assess the effectiveness of a combination behavioral intervention strategy to improve once-daily oral PrEP adherence among Black MSM and TGW in a community setting, compared to a standard-of-care approach | Randomized controlled trial | 204 | Intervention 41  Control 103 | HIV | Intervention n (row%)  18-29: n=50 (54.9%), 30-49: n=32 (40.0%), 50 and above: n=19 (57.6%)  Control n (row%)  18-29: n=41 (45.1%), 30-49: n=48 (60.0%), 50 and above: n=14 (42.4%) | 42 (20.6%) Latinx, Not Latinx 162 (79.4%) | total of 204 participants were enrolled and randomized; 35% were lost to follow-up. PrEP adherence was 30% at 12-months; no intervention effect was observed (p = 0.69). Multivariable regression analysis found that lower adherence was associated with low education and depressive symptoms. We found that an enhanced adherence intervention did not improve PrEP adherence. Findings point to the need for innovative methods to improve PrEP adherence among Black MSM and TGW.  Because DBS were only able to be collected among 46 of 128 participants (35.9%) interviewed at the 6-month time point and 47 of 132 participants interviewed (35.6%) at the 12-month time point, DBS were not utilized to estimate the primary adherence outcome measure. The sensitivity and specificity of self-reported compared to DBS adher- ence at 6-months were 60.9% and 73.9%, respectively. The sensitivity and specificity of self-reported compared to DBS. adherence at 12-months were 45.5% and 88.0%, respec- tively. Notably, there were 6 false negatives comparing self- reported to DBS adherence at 6-months (i.e. participants self-reporting non-adherence but DBS results indicating adherence) and 3 false negatives at 12-months. The median number of medical visits attended for the 93 participants who attended subsequent medical visits was 1 visit (IQR = 1 visit) and the median number of medication bottles picked up for the 125 participants who picked up medication was 3 bottles (IQR = 5 bottles); there was no difference by study arm (p = 0.61 and p = 0.32, respectively). Examining self-reported adherence among all partici- pants over the 12-month study period, 52.9% were adher- ent at 3 months, 42.2% at 6 months, 35.8% at 9 months, and 32.4% at 12 months. Approximately 30% of participants in both arms were adherent, and 60% were non-adherent as per 57% threshold. In both arms, non- adherent participants comprised two roughly equally sized groups of participants who either reported little or no use of PrEP or were lost to follow-up and therefore assumed to be non-adherent.  Sixty of the 101 enPrEP participants (59.4%) met at least once with a peer navigator. The maximum number of visits was 17; the median number of visits was 1 (IQR = 3 visits). According to study navigator forms, peer naviga- tors assisted enPrEP participants with obtaining housing, employment (resume preparation, interviewing skills, job referrals), PrEP adherence counseling, and other needs iden- tified by the participants. Twenty of the enPrEP participants (19.8%) attended at least one of the 13 support group meet- ings. The maximum number of support groups attended was 6; the median was 0 (IQR = 0). No significant linear relationship with 12-month adherence was found for either utilization of intervention components (p = 0.28 for support groups; p = 0.56 for peer navigation visits).  All enPrEP participants received SMS text messages as reminders for adherence and for clinical and research appointments; they were not expected to reply. There was little utilization of the online support group. |
| Paula Anne Newman-Casey et al., 2018  United States | The objective of this study was to assess adult glaucoma patients’ access to and experiences with e-health technologies. We also aimed to evaluate whether technology use differs by medication adherence status and whether technology use differs between older and younger glaucoma patients. | Other: prospective survey study | 185 | 185 | Glaucoma - Physical health | 66.0 (13.7) | Not specified | Eighty percent had good technology access. Seventy-three percent of subjects with greater technology access wanted online glaucoma information and yet only 14% of patients had been directed to online resources by physicians. There was no relationship between technological connectivity and adherence (p=0.51). Nonadherent patients were younger (mean age 58 years vs. 66 years for adherent patients, p = 0.002). Non-adherence was associated with negative feelings about online searches (68% vs. 42%, p = 0.06). |
| Paula Anne Newman-Casey et al., 2020  United States | To assess the efficacy and feasibility of the Support, Educate, Empower (SEE) glaucoma coaching program on medication adherence among glaucoma patients with low adherence. | Other: Uncontrolled intervention study with a prepost design | 46 | 39 completed SEE coaching | Glaucoma - Physical health | 64 years of age (standard deviation [SD], 10.8 years | 46% white | A total of 48 participants were enrolled. The participants were 54% male, 46% white, and on average 64 years of age (standard deviation [SD], 10.8 years), with an average worse-eye mean deviation (MD) of e7.9 dB (SD, 8.8 dB). Those completing the SEE program (n 1⁄4 39) did not differ significantly from those who dropped out (n 1⁄4 9) on gender, race, age, MD, or baseline adherence. Medication adherence improved from 59.9% at baseline to 81.3% (P < 0.0001) after completing the SEE program. Ninety-five percent of participants showed an improvement in adherence (mean relative improvement, 21.4%; SD, 16.5%; range, e3.2% to 74.4%; median, 20.1%). Fifty-nine percent of participants showed adherence of >80% on completing the SEE program. |
| Ponrathi Athilingam et al., 2016  United States | The aim of the developmental project was to design interactive educational modules in a smartphone platform specific for patients with congestive heart failure (HF). This article describes the theoretical foundation of the development of educational materials in a smartphone platform for patients with HF. | Other: Theory-Based Development and Beta Testing | Not specified. | 10 | Heart failure - physical health | Mean (SD) - 63.0 (14.1) years | White - 70%  Other (study did not specify) - 30% | Results of Beta Testing: The 10 participants who completed the beta testing had a mean age of 63 years and ranged from 43 to 81 years of age, of whom 60% were 65 years or older. As shown in Table 2, all10 of the participants were employed full time or part time, 60% were men, 70% were white, and 40% lived alone....All 100% (N = 10) of the participants owned a mobile phone (50% were smartphones), 50% used text messaging, and 60% reported using a mobile phone very well and 40% fairly well.  All participants (100%) reported moderate to extreme confidence in using the app, 95% were very likely to use the app, 100% re-ported the design was easy to navigate, and content on heart failure was appropriate. Almost 70% of participants reported learning new in-formation from the app on HF education and that the information provided in the app was found to be a good re-fresher and of value to patients with newly diagnosed HF for whom they will recommend the app. Having the information accessible on a mobile phone was reported as a health coach or companion promoting persistent engagement to improve health outcomes by all patients. All participants owned a mobile phone, 50% owned smartphones. Those who did not own smartphones (50%) were also able to navigate the app easily.  Clinicians (n = 4) rated the content's congruence with current medical practice and clinical guidelines. Nurses (n = 4) |
| Qiwei L. Wua et al., 2020  Ireland | The purpose of this Short Communication is to report an exploratory analysis of data from the US National Cancer Institute’s 2018 Health Information Trends Survey (HINTS) on factors predicting cancer survivors’ utilization of electronic communication (e-communication) with health care providers.... Guided by SCT (social cognitive theory), this investigation examined the following research question:  Research question: To what extent do cancer patients’ demographic and health-related characteristics, past health care experiences (frequency of seeing providers, quality of communication), and use of technology for health-related purposes (seeking online health information, using health tracking devices such as Fitbit) predict their utilization of e-communication with providers? | Other: Exploratory analysis (published in a Short Communication article) | 593 | 593 | Cancer - physical health (likely different types, but not specified) | Mean (SD) age: 68.09 (13.073) years Age Range: 23~97 years | White - n=481 (81.10%)  Non-White - 112 (18.90%) | In this pre COVID-19 sample, 42 % respondents (N = 252) did not engage in any type of electronic communication (e.g., emailing, texting, data sharing) with providers. In multivariate analyses, predictors of more electronic communication with clinicians included frequency of seeking health-related information online (ß = .267, p<.001) and better communication experiences with clinicians (ß = .028, p = .034), while no demographic variable showed significance. The technology use variables (online health information seeking, health tracking) were significantly higher predictors of electronic communication with clinicians  (triangle sign - DR2 = .142, p < .001) than was past experiences with clinicians (DR2 = .029, p = .016).  Participant characteristics: Most participants were females (N = 344, 58 %) and reported good to excellent physical (N = 443, 74.7 %) and mental health (N = 525, 88.5 %) (Table 2). Education levels varied, and time since cancer first diagnosed cancer ranged from 0 to 87 years (M = 13.83, SD = 13.39). Approximately 42 % respondents (N = 252) had not engaged in any e-communication with providers.  Statistical analyses: In bivariate analyses, e-communication was higher among the younger (r = -.198, p < .001) and more educated respondents  (r = .224, p < .001), as well as those who more often sought health  information online (r = .480, p < .001), tracked health data (r = .430,  p < .001), and who visited providers more often over the last 12  months (r = .173, p < .001)...In multivariate analysis, demographic and health factors (Model 1) explained 7.4 % of the variance in patients’ e-communication, with education being the only significant predictor (ß = .171, p = .001). Variables related to past experiences with providers (Model 2) explained an additional 2.9 % of the variance in patients’ e-communication. While education remained a significant predictor (ß = .179, p < .001), the influence of past patient-centered communication experiences (ß = .026, p = .069) and the frequency of seeing doctors (ß = .071, p = .061) did not reach statistical significance. Technology-use factors (Model 3) explained an additional 14.2 % of the variance in the outcome variable. In this model, patients’ online information-seeking  behavior (ß = .273, p < .001) and past patient-centered communication experiences (ß = .028, p = .031) predicted their e-communication, with marginal effects associated with the frequency of seeing doctors (ß = .057, p = .104) and using electronic methods to track health data (ß = .186, p = .051) (see Table 3 for full regression results). |
| Rishi J. Khusial et al., 2020  United States | To assess the clinical effectiveness and technology acceptance of myAirCoach-supported self-management on top of usual care in patients with asthma using inhalation medication.  We assessed the clinical effectiveness of the mHealth-supported myAirCoach self-management system in patients with asthma compared with usual care and present the results of 2 linked and simultaneously performed studies. | Other: 2 studies: an RCT (Netherlands) and a before and after study (UK) | RCT: 30 B&A: 12 | Intervention RCT: 15 B&A: 12  Control RCT: 15 B&A:N/A | Asthma - physical health | Intervention RCT: 51.3 (13.2) B&A:41.3 (13.8)  Control RCT: 49.1 (11.0) B&A:N/A | Not specified | In study 1, asthma control improved in the intervention group compared with controls (Asthma Control Questionnaire difference, 0.70; P = .006). In study 2, asthma control improved by 0.86 compared with baseline (P = .007) and quality of life by 0.16 (P = .64). |
| Robert D Reid et al., 2011  UK | To compare CardioFit to usual care to assess its effects on physical activity following hospitalization for acute coronary syndromes | Randomized controlled trial | 233 | Intervention 79  Control 108 | Heart disease - Physical health | Intervention 56.7 (9.0)  Control 56.0 (9.0) | Not specified | The CardioFit internet-based physical activity expert system significantly increased objectively measured (p = 0.023) and self-reported physical activity (p = 0.047) compared to usual care. Emotional (p = 0.038) and physical (p = 0.031) dimensions of heart disease health-related quality of life were also higher with CardioFit compared to usual care.  Preliminary analyses showed that age, physical activity level prior to hospitalization, first hospitalization for CHD, and being employed were significantly related to pedometer outcomes at follow up; these variables were used as covariates for subsequent analyses of pedometer data. The repeated measures analysis of covariance for pedometer-measured steps per day showed a main effect only for group. The number of steps per day was higher in the CardioFit group compared to usual care.  Preliminary analyses showed that physical activity level prior to hospitalization, and first hospitalization for CHD were significantly related to self-reported physical activity at follow up; these variables were used as covariates for subsequent analyses related to self-reported activity. |
| Russell E. Glasgow et al., 2003  United States | To evaluate the effects at 10 months following randomization of adding Tailored Self-Management (TSM) training and Peer Support (PS) maintenance components to a basic information-based Internet nutrition intervention. | Randomized controlled trial | Not specified in this article | Not specified in this article | Diabetes - Physical Health | Not specified in this article | Not specified in this article | Website use measured by log ons varied highly across time and conditions, despite our efforts to keep all participants involved with the website throughout the intervention. As seen in Fig. 1a and b, there was consistently greater website use over the first 3-month period for all conditions. Usage dropped off gradually across all conditions during the second 3 months, with the lowest usage rates occurring during the 7- to 10-month period. In addition, across all time periods of the study both the PS and the TSM conditions resulted in more frequent log ons than those not receiving these interventions, with the PS conditions resulting in the most frequent website usage at all time points.  Ten-month assessment results revealed significant improvements from baseline across conditions on the majority of outcomes (Table 2), and the magnitude of these improvements was approximately the same as that seen at earlier follow-ups [22]. Improvements were largest for the targeted dietary outcomes of the Kristal FFB and the Block Fat Screener, next largest for the psychosocial outcomes (perceived barriers and support; depression scores), and more modest for other behavioral outcomes (e.g., improvements in medical care) and biological outcomes (e.g., 12 mg/dl reduction from baseline in cholesterol).As can be seen in Table 3, the pattern of changes generally favored the TSM and PS conditions compared to conditions not receiving these components. However, multivariate analyses to evaluate potential differences between treatment conditions failed to reveal significant incremental effects of either TSM or PS at the 10-month assessment, with the exception of the psychosocial outcomes. Follow-up univariate analyses revealed that this overall effect was due to the PS conditions producing significantly greater improvement on the Diabetes Support scale than among those not in these conditions. Because of the relatively good baseline levels on some measures such as HbA1c, we also conducted analyses of “differential outcome by baseline level” [45,46] in which we included interaction terms between treatment and baseline level on the relevant dependent variable (using median splits). These analyses failed to reveal any differential effects. |
| Ryan P. et al., 2017  UK | To describe the development, implementation and acceptability of the mPeer2Peer intervention, and then discuss implications of the find- ings from this pilot study to the development of future, larger-scale clinical trials in this population. | Qualitative research | 19 | Intervention 15  Control 4 | HIV- Physical health | Median (IQR): 49.3 (45.0-54.6) | 89% African American | Of 19 patients enrolled in the study, 17 participated for at least 2 months and 15 completed the entire 9-month study protocol. The acceptability of the peer navigation intervention was rated favorably by all participants interviewed, who felt that peer support was instrumental in helping them re-engage in HIV care. |
| Ryan P. Westergaard et al.,  2017  UK | mPeer2Peer was a two-component intervention that used a smartphone application and patient navigation delivered by peer health workers (“peer navigators”) to sup-port HIV treatment for patients who had been marginally engaged in care. Peer navigators were trained to deliver intensive psychosocial and logistical support for patients with substance use and other barriers to HIV care. Both patients and peer navigators used a smartphone-based mHealth application, which was developed specifically for this study as a means to enhance communication and enable timely, individually tailored support interventions. This study targeted patients with past or current illicit drug use, whom our prior research has shown to experience frequent lapses in HIV care [24]. Specifically, we aimed to recruit adult patients who were aware of their HIV status, had been linked to HIV care and were pre-scribed ART, but had not successfully achieved viral sup-pression. In this paper, we describe the development, implementation and acceptability of the mPeer2Peer intervention, and then discuss implications of the findings from this pilot study to the development of future, larger-scale clinical trials in this population. | Qualitative research | 19 | 19 (17 completed 2 months of the protocol, and 15 completed the 9-month protocol) | HIV and substance use disorder - both physical and mental health | Median age, years (IQR): 49.3 (45.0–54.6) | African American (%): n=17 (89%) | Between September 2013 and November 2014, 19 individuals were enrolled and randomly assigned to receive the mPeer2Peer intervention. The baseline characteristics of the intervention recipients are shown in Table 1. The study sample was reflective of the population living with HIV in Baltimore, i.e., predominantly Black, male, and low-income, with a median age of 49. 3 years. Most participants reported they were taking antiretroviral therapy at the time of enrollment, yet all but one had an HIV viral load greater than 1000 copies/mL.  Study retention and losses to follow‑up: Of the 19 patients randomized into the intervention group, 15 (78.9%) were followed for the entire 9-month study period. Two participants were immediately lost to follow-up and had no contact with the study team after the enrollment visit; two others were lost to follow-up after month 2 and 6, respectively. Collectively, these 19 participants contributed 143 person-months of follow-up after enrollment. |
| S. Wilson Beckham et al., 2021  UK | In Tanzania, a two-community randomized control trial (RCT) was conducted to evaluate a community empowerment HIV prevention program [23,24]. Using data from this trial, this article seeks to describe family planning use and pregnancy among a population of FSW in Iringa, Tanzania, investigate any differences by HIV status, and evaluate whether engagement in the Shikamana intervention was associated with modern contraceptive use.  This study consists of secondary analysis from a two-community randomized controlled trial following a longitudinal cohort over 18 months. Implemented a year-long community empowerment intervention consisting of 1) a community-led drop-in-center; 2) venue-based peer education, condom distribution, and HIV testing; 3) peer service navigation; 4) sensitivity trainings for providers and police; and 5) text messages to promote engagement. | Other: Secondary analysis of data from 2 RCTs | 339 | Intervention 185 (54.87%)  Control 153 (45.13%) | Physical health: HIV positive or at risk of HIV in female sex workers (FSWs) and family planning | Overall sample size age provided:   Less than/equal to 30 years - n = 186 (54.87%) More than 30 years - n = 154 (45.43%) | Ethnicity:  Groups not local to the region (Iringa region of Tanzania) - n = 139 (41.12%)  Local groups (Hehe, Bena) - n = 199 (58.99%) | Among the 339 participants with follow-up data on family planning, 60% reported current family planning use; 6% reported dual use of modern contraception and condoms; over 90% had living children; and 85% sought antenatal care at their most recent pregnancy. Among the 185 participants in the intervention arm, the adjusted relative risk (aRR) of family planning use among female sex workers who reported ever attending the Shikamana drop-in-center and among female sex workers who reported attending a family planning-related workshop was respectively 26% (aRR 1.26 [95% Confidence Interval (CI): 1.02–1.56]) and 36% (aRR 1.36 [95%CI: 1.13–1.64) higher than among those who had not attended.  Participant characteristics: Just over half of participants were living with HIV. Nearly 30% of participants had ever attended the Shikamana center and only around 17% of participants had ever attended a specific family planning session.  Prevalence of family planning use: Nearly 84% of participants reported ever using modern FP (Table3). Of all study participants, 61% reported current modern family planning use, though 69% of participants who reported that they were not currently trying to become pregnant at the follow-up survey were currently using contraception. As such, over 31% of participants not currently trying to become pregnant demonstrate unmet need for family planning.  Bivariate analysis of modern family planning use: Community, number of clients a week, and history of FP use were significantly associated with current FP use(Table4). Of those currently using FP, nearly 60% of participants were from the intervention community, whereas more than half of those not currently using family planning were from the comparison community. About 45% of participants had two or more clients a week. A larger proportion of those currently using family planning (52%) had two or more clients a week com-pared to those not currently using family planning(35%). |
| Saeed Moradian et al., 2018  Canada | This study aims to evaluate the usability of the ASyMS, a mobile phone–based technology, from the perspective of Canadian patients with cancer receiving chemotherapy to identify existing design, functionality, and usability issues and elicit their views, experiences, and satisfaction with the ASyMS. | Other: mixed-method approach to data collection with user-based testing - usability study | 10 | 10 | Cancer - physical health | Median (range): 68 (18-78) | Not specified | Results from the posttest questionnaire indicated that 80% (8/10) of participants had great motivation to use the ASyMS, 70% (7/10) had positive perceptions of the successful use of the ASyMS, and all (10/10, 100%) had a positive attitude toward using the ASyMS in the future. |
| Sean Arayasirkul et al., 2020  United States | This study assessed the preliminary efficacy and impact of a digital HIV care navigation intervention among young people living with HIV in San Francisco. | Other: Prospective Study (single arm, pre-post design) | Originally 120 enrolled, only 60 completed the study (control not included in the 120). Overall n and %:  Transwoman - n= 17 (14.2%)  Man - n = 103 (85.8%) | 60 (who completed the intervention - 6 months): Transwoman - n = 8 (13.3%)  Man - n = 52 (86.7%) | HIV (human immunodeficiency virus) - physical health | Mean (SD) age of participants who completed the study: 27.93 (4.07) years | Black, non-Hispanic/Latinx - n = 11 (18.3%)  Hispanic/Latinx - 14 (23.3)  Multiple races, non-Hispanic/Latinx - 17 (28.3)  White, non-Hispanic/Latinx - 18 (30.0) | Table 1 presents baseline sociodemographics and HIV care continuum outcomes for the Health eNav sample overall (n=120) and according to the intervention completion status. Except for incarceration and recent receipt of primary HIV care, characteristics according to the intervention completion status were not significantly different from those of the overall sample. The mean age of the participants was 27.75 years (SD 4.07). Most participants (103/120, 85.8%) identified as men. The sample was racially/ethnically diverse, with most participants identifying as Hispanic/Latinx, followed by white, multiple races, and black/African American, and few identifying as Asian or American Indian/Alaska Native. About half (68/120, 56.7%) of all participants completed some college education, yet most lived in unstable housing and had a monthly income of US $1300 or less. Recent incarceration was less likely in participants who completed the intervention than those who did not complete the intervention (11.67% vs. 26.67%, χ21=4.36, P=.04).  In terms of baseline HIV care continuum outcomes, majority (99/120, 82.5%) of the participants had recently received primary HIV care, yet this was more likely in those who completed the intervention than in those who did not complete the intervention (54/60, 90% vs 45/60, 75%; χ21=4.68, P=.03) (Table 1). The 6-month follow-up surveys were completed by 73.3% (88/120) of participants (Table 2), and these participants were not characteristically different from the overall sample at baseline. Table 2 presents the longitudinal results from the GEE models. After analyzing HIV care continuum outcomes over the 6-month study period, we observed that participants had increased odds of viral suppression at 6 months compared with baseline. We observed no statistically significant additive or multiplicative interactions on comparing outcome effects over time according to intervention completion. However, on testing for stratum-specific effects, we found that viral suppression increased over time among those who completed the intervention (83.89% probability of viral suppression at 6 months vs 69.60% probability of viral suppression at baseline; probability difference 14.29%, 95% CI 2.66%-26.41%). No corresponding stratum-specific difference in viral suppression was observed among those who did not complete the intervention. |
| Sean Arayasirkul et al., 2020  United States | This study examines whether the use of MI skills (e.g., OARS) promotes change talk in an SMS text messaging intervention for young people living with HIV in San Francisco. We undertake a novel method of analyzing text message intervention data in order to characterize the microprocesses of change talk. | Feasibility study | Not specified. | 120  Trans woman - n = 17/120 (14.2%)  Man (MSM) - n = 103/120 (85.8%) | HIV (human immunodeficiency virus) - physical health | Mean (SD): 27.8 (4.1) years. | Black, non-Hispanic/Latinx - n = 22 (18.3%)  Hispanic/Latinx - 38 (31.7)  Multiple races, non-Hispanic/Latinx - 28 (23.3)  White, non-Hispanic/Latinx - 32 (26.7) | Across sociodemographic, open-ended questions and affirmations were the most frequently utilized MI skills; reflective listening and summarizing were less common. While 50% or less of Black, Hispanic, or White participants received reflective listening, more than 60% of participants with multiple or “other” race/ethnicity received this skill.  Results show that as the MI skill levels increase, the median instances of change talk tends to increase.  Based on adjustment of total number of texts sent/received, "high levels of OARS are associated with an increased count of change talk compared to low levels (p< .01 for all comparisons)." |
| Shantanu Nundy et al., 2013  Canada | In this study, we pilot-tested a text message–based self-management intervention in an urban, largely African American population for 30 days following hospitalization for ADHF. Our study aims were to assess the feasibility and acceptability of the intervention and to test the hypothesis that the intervention was associated with improvements in self-management. | Other: The pilot was designed as a single-arm prospective study. | 15 | 15 | Acute decompensated heart failure (ADHF) - physical health | Mean (range): 50 (23-69) years | African American: 93%  White: 7% | During the open-ended survey, participants reported that the intervention improved self-management directly by providing reminders, but also indirectly by increasing disease awareness and reinforcing the importance of self-management. They liked that the system served as a reminder and provided feedback. One participant stated, “I knew I wasn’t being forgotten,” and another that, “It’s nice to know that someone cares.” Only 2 participants identified elements they did not like about the system: 1 complained that it was hard for him to text and the other wanted more text messages. |
| Sharon M. Bigelo et al., 2021  United States | The purpose of this paper is to describe the implementation, associated interventions, and outcomes of the PFRC’s proactive virtual resource center navigation model. | Other: report summarizes patient visits between 3/24/2020 and10/23/2020 at NCCH | 586 | 586 | cancer - physical health | 18-39: 12.1%, 40-64: 46.8%, 65+: 41.1% | White 46.1%, Black 25.1%, Asian 1.2%, Native 0.2%, Latinx 1.0%, Other 26.4% | The program engaged with 586 adult cancer patients over 1459 encounters. The most common risk factors included distance (59.7%), complex care (48.8%), and new treatment start (43.5%). The most common interventions were core education (69.4%), emotional support (61.2%), and education (35.7%). Statistical differences were found between Spanish-speaking (n = 118) and non-Spanish-speaking patients (n = 468). While Spanish-speaking patients had fewer risk factors (1.95 vs. 2.80, p ≤ .0001), they had nearly double the number of visits (4.27 vs. 2.04, p ≤ .0001) and 69% more interventions (8.26 vs. 4.90, p ≤ .0001). Many patients (42.7%) required follow-up visits. |
| Silvia Cacho-Elizondo et al., 2013  United States | This article concerns a mobile coaching service providing support for people trying to stop smoking. The service takes the form of short text messages (SMS or MMS) sent to cell phones to help individuals in a range of situations or anti-smoking activities. The principal objective of this study is to identify drivers fostering the intention to adopt such a service in the young smokers segment in France. The article is structured as follows. Firstly, the conceptual framework is presented and after that the model of the intention to adopt the mobile coaching service is introduced. The methodology is then described along with the operationalization of the underlying hypotheses. After reporting the main findings and managerial and social implications, the paper concludes by considering limitations and avenues for future research. | Other: Explanatory model | N/A. | N/A | Smoking addiction (nicotine, tobacco) - mental health | No intervention actively tested - study about intention to adopt mobile coaching intervention for smoking cessation. Survey participants age ranges provided:  14-17 years - n=48 18-21 years - n=38 22-25 years - n=21 Over 25 years - n=6 | Not specified/provided. | Descriptive analysis:   The sample shows a homogeneous distribution between men (52%) and women (48%). 76% of participants are aged between 14 and 21 (N=86). More than 80% of participants (N=92) state that they smoke every day or nearly every day. Smokers can be grouped into three categories according to the number of cigarettes smoked per day: light smokers (N=32, 28%), medium smokers (N=40, 36%) and heavy smokers(N=41, 36%) (see Tables 2 and 3). Of the 113 participants in the study, only 37 stated that they intended to stop smoking in the next 12 months; 76 did not share that intention. However, the average intention to adopt the service does not differ significantly between the two groups, i.e. between people who say they want to stop smoking and the rest (F=2.62 (0)).  Among the people who intend to stop smoking in the next 12 months (N=37), most are Heavy Smokers (more than 10 cigarettes a day) and Light Smokers (fewer than 5 cigarettes a day). Medium Smokers (5 to 9 cigarettes a day) have proportionally less intention of giving up smoking; probably because, according to the arguments collected in the exploratory study they are less worried about their budget and their health, or are under less pressure from their entourage.  One of the barriers to adopting the service....is the lack of human contact in the coaching service. This is observed more with individuals over 18 than individuals under 18. Analysis of the variance shows a significant difference between the averages for the two groups of individuals. Of the 43 individuals who would not use this service because of the lack of human contact, 22 think it could help someone else to stop smoking.  Discussion: The intention to adopt the text message-based mobile coaching service to help stop smoking was tested with a mostly young target. This is precisely the group with the greatest risk of developing tobacco-related illnesses in the long term, since the lower the age at which people start smoking, the higher their risks of serious tobacco-related illnesses. Yet the study observed that these young smokers do not always intend to stop smoking in the short term. It is important to find innovative ways to motivate them to try and start the process of giving up smoking. With this in mind, mobile services can play an important role because young people are generally heavy users of cell phones and generally find new technologies attractive (Syed & Nurullah, 2011). |
| Stephen Miller et al., 2020  Canada | To explore the usability, acceptability, and utility of one such symptom assessment technology, Ada, in a primary care setting. | Other: Descriptive usability and acceptability study, | 523 | 523 | Various conditions/symptoms | 39.79 (17.4) | Not specified | Over a 3-month period, 523 patients participated. Most were female (n=325, 62.1%), mean age 39.79 years (SD 17.7 years), with a larger proportion (413/506, 81.6%) of working-age individuals (aged 15-64) than the general population (66.0%). Participants rated Ada’s ease of use highly, with most (511/522, 97.8%) reporting it was very or quite easy. Most would use Ada again (443/503, 88.1%) and agreed they would recommend it to a friend or relative (444/520, 85.3%). We identified a number of age-related trends among respondents, with a directional trend for more young respondents to report Ada had provided helpful advice (50/54, 93%, 18-24-year olds reported helpful) than older respondents (19/32, 59%, adults aged 70+ reported helpful). We found no sex differences on any of the usability questions fielded. While most respondents reported that using the symptom checker would not have made a difference in their care- seeking behavior (425/494, 86.0%), a sizable minority (63/494, 12.8%) reported they would have used lower-intensity care such as self-care, pharmacy, or delaying their appointment. The proportion was higher for patients aged 18-24 (11/50, 22%) than aged 70+ (0/28, 0%). |
| Steven C. Martino et al., 2019  United States | We conducted a simulated clinician-choice experiment, comparing choices and decision-making processes of participants randomized among four experimental arms | Randomized controlled trial | 688 | No comments: 175, Conventional Comments: 176, Tagged Comments: 179, Tagged Comments + Navigator: 158 | Various health conditions - Physical and mental health | No comments:18-29 14.3%, 30-44 19.4%, 45-60 30.9%, >60 35.4% ,   Conventional Comments: 18-29 13.6%, 30-44 22.2%, 45-60 30.1%, >60 35.4% ,   Tagged Comments: 18-29 14.0%, 30-44 20.7%, 45-60 29.1%, >60 36.3% ,   Tagged Comments + Navigator: 18-29 12.0%, 30-44 22.8%, 45-60 29.8%, >60 35.4% , | No comments: 79.4% White  Conventional Comments: 78.4 % White  Tagged Comments: 74.3% White  Tagged Comments + Navigator: 79.8 | Introducing patient comments enhanced engagement with the quality information but led to a decline in decision quality, particularly the consistency of choices with consumers’ stated preferences. Labeling comments helped erase the decline in decision quality, although the highest percentage of preference-congruent choices was seen in the navigator arm. Engagement with the quality information and satisfaction with choices available were likewise highest in the navigator arm. Findings held for high- and low-skilled decision makers. Thus, navigator assistance may be a promising strategy for equitably promoting higher quality choices in information-rich contexts  Overall, patients tended to be satisfied with the website (75% said they would recom-mend the site to others who were choosing a doctor) and report that the metrics on the site were easy to understand (70%). However, less than half the sample (46%) said that it was easy to make trade-offs among the metrics included on the site and more than two thirds selected clinicians who were inconsistent with their stated preferences.  The design and content of the website had little evident impact on the scope of outcomes considered, ease of making trade-offs, and consumers’ ability to understand particular quality metrics (all p values for the omnibus test of cross-arm differences ≥0.13; see Table 3). |
| StutiDang et al., 2017  Canada | To test the usability and obtain patient perspectives of a mobile phone intervention for heart failure in a minority urban county hospital population | Randomized controlled trial | 42 | 42 | Heart failure patients - Physical health | age ranged from 27 to 83 years with a mean–standard deviation (SD) of 53.0–9.4 | 76% White Hispanics, 21% African Americans, | From abstract: Forty-two participants aged 53.0 – 9.4 years (mean – standard deviation) were randomized to the mobile-monitoring intervention group. They included the following: 67% males, 76% White Hispanics, 21% African Americans, and 52% with high school education or less. Over the 3-month inter- vention period, 26 (62%) participants used the system over 50% of the time. Overall, on a 1.0–7.0 scale for both, program satisfaction scores were excellent (mean 6.84 – 0.46), and the usability ratings were all above 6.0. Comparing 1- to 3-month responses, there was a substantial increase in the percentage of participants who felt the system was easy to use after they had gotten used to it(84%vs.94%) and that navigating the system was not complicated (78% vs. 84%). |
| Susan D. Newman et al., 2019  UK | To develop educational content and pilot test the use of tablet computers (iPads), online content management platform (iTunes U) and video conferencing (FaceTime) for delivery of a peer supported, spinal cord injury self-management intervention, using a community-engaged research approach | Cross sectional study | 10 | 10 | Spinal Cord Injury - Physical health | Median (range): 48.5 (36-70) | White: 30%, Black: 70% | Participants were receptive and satisfied with the iPad and iTunes U platform and the video chat experience. Statements by our participants demonstrated a clear preference for interactive and multimedia platforms to promote engagement with educational materials. The use of FaceTime to facilitate contact between the participant and PN demonstrated satisfactory usability and acceptability. |
| Susan E. Appling et al., 2016  United States | To develop and evaluate a centralized “virtual” navigation program to support breast cancer patients through their adjuvant treatment. | Randomized controlled trial | 98 | Intervention 49  Control 49 | Breast cancer - Physical health | Intervention 52.9 (9.9)  Control 48.7 (10.0) | Intervention: White: 34.7% Black: 63.3% Other: 2.0% Control: White: 30.6% Black: 65.3% Other: 4.1% | From abstract: Of the participants, 67% were minorities, mostly African American. Slightly more of the group randomized to the navigator team reported that the application was easy to use (77.6% vs 71.4%) and with higher confidence (71.4% vs 67.3%), but these differences were not statistically significant. |
| Tanya Millard et al., 2016  United States | The aim of this paper was to evaluate the effectiveness of an online self-management program in improving health outcomes and well-being for gay men living with HIV in Australia. | Randomized controlled trial | 132 | Intervention 62  Control 64 | HIV- Physical health | Intervention 42.6 (10.5)  Control 42.0 (10.5) | Not specified | From abstract: A total of 132 gay men with HIV in Australia were randomly allocated to the intervention (n = 68) or usual care control (n = 64) groups. Maximum likelihood marginal-linear modelling indicated significant improvement in the intervention group on the PROQOL-HIV subscales of body change (p = 0.036), social relationships (p = 0.035) and emotional distress (p = 0.031); the HeiQ subscales of health-directed activity (p = 0.048); constructive attitudes and approaches (p = 0.015); skill and technique acquisition (p = 0.046) and health service nav- igation (p = 0.008); and the Positive Outlook Self-Efficacy Scale on the subscales of relationships (p = 0.019); social participation (p = 0.006); and emotions (p = 0.041). |
| Tuula Karhula et al., 2015  Canada | The purpose of this 1-year trial was to study whether a structured mobile phone-based health coaching program, which was supported by a remote monitoring system, could be used to improve the health-related quality of life (HRQL) and/or the clinical measures of type 2 diabetes and heart disease patients. | Randomized controlled trial | Heart disease patients: 269, Diabetes patients 250 | Study broke down heart disease patients into a control and intervention group and then diabetes patients into a control and intervention group. Heart disease intervention group: 190, Diabetes intervention group: 180  Study broke down heart disease patients into a control and intervention group and then diabetes patients into a control and intervention group. Heart disease control group: 79, Diabetes control group: 70 | Type 2 diabetes and heart disease - Physical health | Study broke down heart disease patients into a control and intervention group and then diabetes patients into a control and intervention group. Heart disease intervention group: 69.6 (9.1), Diabetes intervention group: 180 66.6 (8.2)  Study broke down heart disease patients into a control and intervention group and then diabetes patients into a control and intervention group. Heart disease control group: 68.1 (9.4), Diabetes control group: 65.5 (9.6) | Not specified - Patients of South Karelia Social and Health care district | A total of 267 heart patients and 250 diabetes patients started in the trial, of which 246 and 225 patients concluded the end-point assessments, respectively. Withdrawal from the study was associated with the patients’ unfamiliarity with mobile phones—of the 41 dropouts, 85% (11/13) of the heart disease patients and 88% (14/16) of the diabetes patients were familiar with mobile phones, whereas the corresponding percentages were 97.1% (231/238) and 98.6% (208/211), respectively, among the rest of the patients (P=.02 and P=.004). Withdrawal was also associated with heart disease patients’ comorbidities—40% (8/20) of the dropouts had at least one comorbidity, whereas the corresponding percentage was 18.9% (47/249) among the rest of the patients (P=.02). The intervention showed no statistically significant benefits over the current practice with regard to health-related quality of life—heart disease patients: beta=0.730 (P=.36) for the physical component score and beta=-0.608 (P=.62) for the mental component score; diabetes patients: beta=0.875 (P=.85) for the physical component score and beta=-0.770 (P=.52) for the mental component score. There was a significant difference in waist circumference in the type 2 diabetes group (beta=-1.711, P=.01). There were no differences in any other outcome variables. |
| V.C. Sánchez-Ortiz et al., 2011  Italy | To date, no study has used robust qualitative methodology to examine the views of people using internet-based self-care treatments for bulimic disorders. The aim of the present study was to develop an in-depth understanding of the views and experiences of iCBT participants using interviews and questionnaires. | Qualitative research | 9 | 9 | Bulimia nervosa and other related (eating) disorders - mental health | Mean (SD) age: 23.2 years (SD=3.5) | Race/ethnicity not specified, but:  Five participants were British nationality and four were international students. | Questionnaire responses echoed themes identified in the interviews. iCBT was received positively as a way of fitting treatment into busy lives. Comments on the layout of some of the package content and the practitioner support offered were identified as areas that could be modified or improved.  Participants provided detailed accounts of their experience of using iCBT. In-depth inter-views revealed five key themes, and some consisted of several categories.  1. Reasons for choosing this form of treatment  2. Experiences of treatment: - sub-themes - (I) Confidentiality/privacy; (II) Flexibility; (III) Ease of use; (IV) Feeling supported - including help with motivation; and (V) Content of programme  3. Impact of treatment: - sub-themes - (I) Expectations about outcome; (II) Effectiveness - Changes in ED symptoms; (III) Effectiveness - Other changes; and (IV) Tools for coping in the future  4. Comparison between iCBT and other forms of treatment: - sub-themes - (I) Counselling; (II) General Practitioner (GP); and (III) Other Forms of Self-help  5. Feedback: - sub-themes - (I) Timing; and (II) Other methods of support |
| Veronica P. S. et al., 2020  UK | To evaluate perceived usefulness, satisfaction, acceptability, intervention processes, resource management, and outcome effect variances of ThE CARE Intervention | Other: proof of concept (PoC) study | 20 | 20 | HIV - Physical health | 56.11 (11.05) | Not specified | ThE CARE intervention was found useful and participants “felt empowered” utilizing the app. Fourteen participants (70%) reported high-intensity distress and negative impact on life from neuropathic pain, anxiety (55%), fatigue (50%), and depressive symptoms (35%). Self-awareness and self-regulation also improved. |
| Xia Jin et al., 2019  Canada | The objectives of this study were (1) to assess the capability of the Easy Test model in increasing access to HIV testing and treatment services for MSM who have never tested for HIV and (2) to identify demographic and behavioral predictors of program uptake to inform future implementation. | Feasibility study | 879 | 879 | HIV- Physical health | < 25 years 26.6%, 25-34 50.3%, > or equal to 35 23.1% | Han 91.9%, Other 8.1 | Total of 879 individuals submitted Web-based requests for test kits. Their median age was 28 (interquartile range 24-34 years); 69.3% (609/879) had at least a college education, and 51.5% (453/879) had a monthly income between US $450 to $750; 77.7% (683/879) of the applicants submitted images of their test results, among whom 14.3% (98/683) had an HIV-positive result. Among the 42.9% (293/683) who were first-time testers, the HIV prevalence was 18.8% (55/293). Nearly three-quarters (71/98, 72.4%) of those with a positive test result were connected with a peer navigator and enrolled in treatment. Among the first-time testers, having multiple sexual partners (2-3 sexual partners: adjusted odds ratio [aOR] 2.44, 95% CI 1.08-5.50; 4 or above sexual partners: aOR 3.55, 95% CI 1.18-10.68) and reporting inconsistent condom use in the previous 3 months (aOR 7.95, 95% CI 3.66-17.26) were both associated with an HIV-positive result. An inverse dose response relationship between lifetime  HIV testing volume and HIV prevalence was also observed in this study (χ2 =55.0; P<.001). |
