# Supplementary material for: Characteristics of Existing Online Patient Navigation Interventions: Scoping Review
Source: JMIR Med Inform. 2024 Aug 19;12:e50307. doi: 10.2196/50307 (PMC11369544; doi:10.2196/50307)
Supplement: Multimedia Appendix 4 [file medinform_v12i1e50307_app4.docx]

| **Author, Year, Country** | **Intervention Type** | **Duration** | **Frequency** | **Mode of Delivery** | **Underlying Theories** | **Outcome Measures** | **Outcome** |
| --- | --- | --- | --- | --- | --- | --- | --- |
| Abbey B. Berenson  et al., 2016  United States | Patient | A program evaluation was conducted after 20 months | 3 vaccine doses - but frequency of PN not specified - variable. | Hybrid | Not specified | Bivariate analyses compared characteristics between patients who initiated the HPV vaccine postpartum with those who declined. In addition, adjusted multivariable logistic regression analysis was conducted to examine correlates of HPV vaccine initiation and completion among all enrolled patients, series completion among postpartum initiators and series completion within 6 months of postpartum initiation (the timeframe recommended by the CDC).22 We only included characteristics that had an association with the outcome with a p value <0.2 value in initial analyses of the associations. Age at first sexual intercourse and number of lifetime sexual partners were not included in the multivariable model due to excessive missing data. We also examined associations with missing an appointment for follow-up vaccine doses using adjusted logistic regression analysis. Statistical tests were based on 2-tail and a p value <0.05 was considered as statistically significant. All statistical analyses were conducted using Stata Statistical Software (Stata 14, College Station, Texas, USA). | Among those screened on the postpartum unit, only 25.4% had previously received any HPV vaccines. At the time we conducted this assessment, 80.8% of patients had received at least 1 vaccine, and completion rates rose from 15.5% at baseline to 65.1% as a result of this program. Among new initiators who completed the series (n=436), 265 (60.8%) patients completed the series within 6 months as recommended by the CDC. The average time between the first and second dose was 2.3±2.1 months and the average time between the second and third dose was 5.6±2.5 months. A total of 1,651 vaccine doses were provided for the 575 new initiators and 103 incompletely vaccinated patients who participated in this program. Almost 59% of doses were funded by Medicaid, 36% by CPRIT, and 5% by private insurance. CPRIT funded 2%, 42% and 78% of the first, second, and third doses, respectively. A higher proportion of those that did not miss follow-up appointments completed the vaccine series compared to those that missed at least 1 appointment (87.9% vs. 70.2%, p<0.001). Among 575 postpartum HPV vaccine initiators, 182 (31.7%) attended all appointments while 393 (68.3%) missed at least one follow-up appointment. Further analysis of missed appointment data showed that non-Hispanic blacks and patients with two or more prior pregnancies were more likely to miss one or more follow-up appointments (Table 3). Women ≥21 years of age and those who received automated text messages were less likely to miss an appointment. Automated phone calls and text messages were received by 87.0% and 78.8% of patients, respectively, prior to any appointments. The median number of times PNs called a patient due to missed appointments was 4 (interquartile range 1–8). Overall, 12.7% of women in the project received letters and 6.1% received emails because they missed appointments and could not be reached by phone. Of the 72 patients that initiated the HPV vaccine postpartum and were lost to follow-up, 34 patients moved out of Galveston County (5.9% of vaccine initiators), 29 patients (5.0%) changed their telephone number or address and could not be found, and 9 (1.6%) were lost to follow-up for other reasons. From ABSTRACT: Of 1,038 patients approached, only 161 (15.5%) had previously completed the vaccine series. Of the 877 patients who had not completed the series, 661 (75.4%) received at least one dose postpartum, with 575 patients receiving their first dose and 86 receiving their second or third doses. By April 2015, initiation rates had increased as a result of this program from 25.4% before the program was initiated to 80.8% and completion rates from 15.5% to 65.1%. Missed appointments for injections were less likely among those who received text message reminders and more likely among those with ≥2 prior pregnancies. Those who were Hispanic or had received an influenza vaccination in the last year were more likely to initiate and complete the series through this program. Patients who missed 1 or more follow-up appointments were less likely to complete the vaccine series....Offering the human papillomavirus vaccine postpartum dramatically increased initiation rates among postpartum patients. Patient navigation and text messages ensured that a high percentage completed all 3 doses. |
| Ahmed Fadhil et al., 2019  United States | Other: Remote health coaching | Participants’ behavior was measured during the 4-week validation study | Variable based on online behaviour/participation | Online | Not specified - Poor lifestyle represents a health risk factor and is the leading cause of morbidity and chronic conditions. Behavioural modification theory? | The objective was to measure participants’ engagement in health behavior change activities, their acceptance and use of the system, and their rating of system usability and design. Therefore, three methods (namely, HAPA, TAM, and Attrak- Diff) were used to validate our evaluation objectives and measure any outcome that might emerge from these valid tions. The intervention-measured effectiveness was categorized based on the studies of Lentferink et al45 and Morrison et al.46 This divides the intervention outcome into three catagories, namely highly effective, low effective, and ineffective interventions (see ►Table 1 for the effectiveness criteria). We listed the outcome measure per objective based on the results we obtained from users’ experiment. In ►Table 2 we list the methods and the evaluation objectives with the outcome per method. | The system calculated participants’ overall adherence to the plan and reported their total adherence at the end of each plan expiration. The adherence was categorized into high and low adherence groups. Users performing above the threshold were categorized as highly adherent, and vice versa. Participants were categorized according to their adherence to the plan into high and low adherence, with respectively 10 and 9 participants each. The TAM dimensions were analyzed with a multivariate ANOVA (MANOVA) with “adherence level” as between subject factor. The MANOVA shows a significant effect of the between subject factor for usefulness (F(1,16) 1⁄4 6.5, p < .01), fun (F(1,16) 1⁄4 4.5, p < .01), and attitude (F(1,16) 1⁄4 6.9, p < .01). No differences were found for the ease of use and intention. |
| Aliza Selter Christina et al., 2018  Canada | Other: Combination of patient navigation which includes support from a health coach. | 3 months | Variable | Online | Conditions for which exercise therapy has been shown to be effective, such as chronic lower back pain (CLBP) [6], stand to benefit greatly from mHealth integration because sustained adherence to exercise-based rehabilitation is vital for recovery [7-9]. | 1. Patient Engagement Patient engagement was assessed using three outcome variables: (1) the frequency of interactions across the visual self-reports, (2) a binary outcome representing at least one viewing of the physical therapy videos versus none watched, and (3) the frequency of messages to the health coach. |  |
| Amanda Gehrke et al., 2018  United States | Patient | The CSPro-Breast requires approximately 15 to 20 min to complete | Not specified | App | Not specified - In order to optimize today’s healthcare delivery, especially for complex chronic illnesses, collaboration among providers and patients is imperative [52, 53]. When this kind of team- work is executed successfully, treatment outcomes can often be improved, particularly in the case of prepared, proactive providers working with informed, activated patients | Perceptions of the CSPro-BC app were evaluated using a multi- ple method design, including both quantitative and qualitative approaches | BCS (N = 11) were middle-aged and a median of 2.4 months post active treatment. Likert ratings indicated that the majority of BCS (91%) found the graphic profile represented each of the 18 prob- lem areas in an understandable manner, and all BCS thought that it was a useful communication tool to discuss problem areas with their nurse. No BCS endorsed the statement that it Bseemed like a waste of time—I knew this already.^ Ratings indicated that 82% of BCS thought the graph could Bstand on its own with little explanation.^ It was also observed that the majority of scores (bars on the graph) fell into the yellow or Bwatch^ category. Patients often wanted to discuss these yellow areas with the nurse navigator and requested additional resources for these areas, as well. While most BCS (89–91%) thought the problem-specific resources would be helpful, some were not certain.  Structured questionnaires indicated the following: survey covered meaningful problem areas, profile display was clear, and nurse’s involvement was helpful. Follow- up interviews (2 weeks later) revealed that BCS shared their profile with others, but most BCS did not use the resources and those who did thought there were too many. Nurses (N = 3) said the app increased appointment time, but prompted them to discuss areas often not covered in typical BCS follow-up. Common themes that emerged from follow-up surveys with participants include: In terms of content:Additional information needed, profile layout helpful, could be better, Experienced information overload with online resources, Preferred another format, In terms of use: Beneficial to share profile with others, Profile beneficial to care, Online resources not accessed Nurse navigator feedback: Each nurse navigator indicated the CSPro-BC app rationale was clear, and that the graphic output provided understandable and clinically useful patient informa- tion. The nurse navigators also noted that the process of using the CSPro-BC app facilitated communication around sensitive topics (e.g., sexual function/intimacy) that BCS are often hes- itant to discuss during follow-up visits. The nurse navigators expressed that the additional time allocated to review the pro- file and provide feedback did lengthen the visit. When asked whether the additional time was justified, the nurses reported that it was, but also mentioned that it was primarily up to the patients as whether the time was beneficial. |
| Anetta Hinchliffe & W. Kerry Mummery, 2008  Australia | Other: Health promotion, web-based support | N/A | N/A | Online | N/A | During the session the researcher recorded time to complete each task, taken by a stopwatch. Time was taken from the time the task was read aloud, until the specific task was completed. All timing was done by the principal researcher. Immediately following the completion of the tasks on the website, each user recorded their perception of their experience with the website on a series of five-point Likert scale questions. | Comparing the problem counts of UT1 and UT2 showed a significant decrease in the number of unique problems (t12=2.95, p=0.004) and the problems-per-user (t12=2.54, p=0.03). Table 1 provides an overview of the problems found in both usability testing sessions The mean time taken to complete the 14 tasks in UT1 was 21.59 minutes (SD±4.8) and in UT2 was 10.18 minutes (SD±6.2). Overall, the modifications to the website resulted in the mean time to complete the tasks decreasing by 52% (9.05 minutes). An independent sample t-test revealed this as a significant decrease in time (t10=3.56, p=0.005) Internal consistency (Cronbach’s alpha) for the usability factor (α=0.88) and credibility and content factor (α=1.00) were high, indicting acceptable reliability of the measure. Analysis showed a significant improvement in the usability score (t10=-2.636, p=0.025) with the means improving from 3.13 (SD±0.59) on the pre-test to 4.10 (SD±0.68) subsequent to the modifications. No significant change was observed in terms of credibility and content (t10=-0.542, p=0.599), with the pre-modification mean being 4.33 (SD±0.51) compared with a post-modification mean of 4.50 (SD±0.55) |
| Anjana Das et al., 2019  United States | Peer | 6 months | Not specified exclusively, but variable. | Online | Not specified | Not specified clearly, but based on this description: Eligible MSM were asked for information pertaining to sociodemographic profile and risk behaviors and offered pretest counseling. Those who gave written informed consent (as per norms followed by the HST ICTC) were tested for HIV and syphilis and asked to return the next day for posttest counseling. Individuals with positive test results for HIV, syphilis, or both were referred to tertiary hospitals. All participants received prevention education during pre- and posttest counseling, were offered assistance for registering with targeted interventions for ongoing services, and received prevention messages from the coupon manager at regular intervals via WhatsApp in which group members could not view others’ contact details. At the posttest visit, MSM willing to be enrolled as peer mobilizers were oriented to the project by the coupon manager and given tips on how to motivate other MSM to avail HTS. Coupons given to peer mobilizers had a validity period of 30 days. If none or only some of the coupons had been used within the time period, peer mobilizers were contacted and requested to encourage their peers to attend the clinic. | In the period January to July 2017, messages on social media were sent to 5,530 MSM and 1,030 MSM made online inquiries. Through social media and coupon referrals, a total of 274 individuals attended the clinic, of whom 27 were ineligible because they either had received targeted intervention services (n=23) or were less than 18 years old (n=4). Thus, 247 MSM were enrolled, which included 22 primary seeds (first-wave peer mobilizers), subsequent waves of peer mobilizers, and others unwilling to be peer mobilizers. The numbers of MSM recruited from each network generated from the 22 primary seeds (not shown) varied greatly. The mean size of the 5 largest networks was 39.8 (range 13–81), while the mean size of 11 networks was 3.8 (range 1–7); 6 primary seeds did not refer others. Two-thirds (69%) were less than 25 years old and their preferred social media platforms were Facebook, Grindr, IMO, Instagram, PlanetRomeo, Tinder, and WhatsApp. Nearly half (44%) reported inconsistent or no condom use during the last 10 acts of anal sex, and some reported other highrisk behaviors such as transactional sex, group sex, and substance use during sex. Among the 247 participants, 244 (99%) were first-time testers. The prevalence of HIV and high-titer syphilis was 3.2% and 8.9%, respectively. Half of those with HIV diagnosed were successfully linked to treatment, and all but one of those with a positive test result for syphilis attended the referral hospital for treatment. |
| Anne Looijmans et al., 2019  UK | Other: Lifestyle coaching supporting behavioural change | 12 months | ideally once every two weeks | Online | Despite the multidisciplinary guideline for SMI patients recommending to monitor and address patients’ lifestyle, most mental health care professionals have limited lifestyle-related knowledge and skills, and (lifestyle) treatment protocols are lacking.  Systematic reviews on lifestyle interventions in different populations indicate that, to be effective, a lifestyle intervention should contain at least three key components: exercise, diet and behavioural therapy. Behavioural therapy strategies that enhance individual behavioural change include improving self- management skills such as tailoring information to the individual, identifying (lifestyle) areas for improvement, goal setting, making action plans, giving personalized feedback to reinforce new behaviours and using social and environ- mental strategies to support change The tool provides knowledge and incorporates behavioural techniques to elicit behavioural change, such as creating awareness, goal setting, providing feedback and self-management. Unhealthy lifestyle behaviours contribute to alarming cardiometabolic risk in patients with serious mental illness (SMI). | Primary outcome is waist circumference and other cardiometabolic risk factors after six and twelve months intervention, which are measured as part of routine outcome monitoring using standard protocols. Secondary outcomes include depressive and negative symptoms, cost-effectiveness, and barriers and facilitators in intervention implementation. | General multilevel linear mixed models adjusted for antipsychotic medication showed that differences in WC change between intervention and control were − 0.15 cm (95%CI: − 2.49; 2.19) after six and − 1.03 cm (95%CI: − 3.42; 1.35) after twelve months intervention; however, the differences were not statistically significant. |
| Astrid Torbjørnsen et al., 2014  United States | Patient | 1 year | Variable | Other: App + Health counseling (through phone calls + text messaging) | Due to costly treatment, it may become necessary to differentiate between those in need of a low- or high-intensity intervention, thereby offering the patients the lowest level of effective management [11] and reducing the costs...Furthermore, the development of self-management support is recommended by international guidelines because it has also been shown to have an effect on glycemic control [14,15]...Computer-based solutions may support self-management in everyday life and research shows that mobile health tools in particular may improve glycemic control, although the findings are inconclusive [16-18]. Furthermore, few telemedicine studies have detected effects on cognitive, behavioral, or emotional outcomes [17], and few studies have measured self-management using appropriate questionnaires. Some interventions combine self-monitoring with professional support, which is based primarily on the monitoring of results by health care providers, with subsequent counseling and advice [18-22]. More research is needed in this area to determine the effects on both clinical outcomes and self-management, and to assess the benefit of providing health counseling to support patients in the implementation and maintenance of the necessary behaviors required to manage their diabetes [15]. | We used a broad evaluation based on a complex intervention framework [32] and MAST [23]. The Consolidated Standards of Reporting Trials (CONSORT) statement for reporting of RCTs [33], CONSORT for pragmatic trials [25], and the eHealth checklist [34] were used. The primary and secondary outcomes are described in Table 1, as well as the time points for the assessments. | Data were analyzed from 124 individuals (attrition rate was 18%). The groups were well balanced at baseline. There were no differences in HbA1c between groups after 4 months, but there was a decline in all groups. There were changes in self-management measured using the health service navigation item in the heiQ, with improvements in the FTA group compared to the control group (P=.01) and in the FTA with health counseling group compared with both other groups (P=.04). This may indicate an improvement in the ability of patients to communicate health needs to their health care providers. Furthermore, the FTA group reported higher scores for skill and technique acquisition at relieving symptoms compared to the control group (P=.02). There were no significant changes in any of the domains of the SF-36. Demographic info: There were no statistically significant differences between the groups in terms of the baseline variables, except for rheumatism and depressive symptoms (Table 2). Significantly more participants in the FTA group had rheumatism compared with both of the other groups (n=11, 4, and 3 in the FTA, FTA with health counseling, and control groups, respectively, P=.03). More individuals had depressive symptoms (a CES-D score ≥16) in the control group (n=17) compared with the FTA group (n=10) and the FTA with health counseling group (n=7, P=.045). Of the 151 participants, the mean age was 57 years (SD 12), 62 (41.1%) of participants were women, and 83 (55.0%) had less than 12 years of education. The mean HbA1c was 8.2% (SD 1.1) or 66 mmol/mol (SD 12), the mean BMI was 31.7 kg/m2 (SD 6.0), and 58.1% (75/129) were obese [43]. Only 9 of 131 participants (6.9%) did not receive glucose-lowering medication. In total, almost half of the participants (72/151, 48%) reported 2 or more comorbidities and 36 of 151 (23.8%) reported heart disease. Primary Outcomes and Estimations: In total, 118/151 (78.2%) participants provided HbA1c data at 4 months. There were no statistically significant differences in HbA1c level changes from baseline between the 3 groups (P=.65) after 4 months (Table 4). Adjustments for age, gender, and education did not affect the estimates. The mean HbA1c level declined in all groups: –0.41 (95% CI –0.71 to –0.11) in the FTA with health counseling group, –0.23 (95% CI –0.47 to 0.01) in the FTA group, and –0.39 (95% CI –0.75 to –0.03) in the control group. Characteristics in Responders Versus Nonresponders: When comparing distribution of variables at baseline and at 4 months in responders versus nonresponders, there were no significant differences between the groups. Hence, our analyses of dropouts vs nondropouts indicated that attrition did not change the distribution between the groups at baseline (Table 3). |
| Benjamin E. et al., 2021  United States | Other: Social worker and patient navigation | Not specified - Participants were followed up for a minimum of 22 months post-randomization | Not specified, variable | Other: Confirming appointments by email and text messaging | Appointment non adherence is common among people with glaucoma, making it difficult for eye care providers to monitor glaucoma progression. | The primary outcome measure was adherence to recommended follow-up eye care appointments after Visit 3. Adherence was assessed annually on the basis of the expected follow-up schedule defined at the index visit for that year. In the first year, the follow-up recommendation given at Visit 3 by the ophthalmologist was classified into 1 of 4 categories: return with-in 2 months, return in 3 to 4 months, return in 6 months, or return in 12 months. | Timely attendance at the first visit was higher for EI than UC (74.4% vs 39.0%; average relative risk [aRR] = 1.85; 95% CI,1.51–2.28; P < .001). Rates of adherence to recommended annual follow-up during year 1 were 18.6% in the EI group and 8.1% in the usual care group (aRR = 2.08; 95% CI, 1.14–3.76; P = .02).The aRR across years 2 and 3 was 3.92 (95% CI, 1.24–12.43; P =.02) |
| Bonnie Spring et al., 2017  United States | Other: Coach + combination of education from psychologist, physiologist - which is patient navigation + interaction from social/other members | 6 months intervention (follow-up at 6- and 12-months) | Variable | Other: In-person sessions, calls, texts, app, coach, etc. | Smartphones offer a promising intervention channel and self-regulation tool, particularly as ownership continues to rise: from 46% in 2012 to 67% in 2015.7 Smartphones hold potential to reduce treatment burden and increase reach by replacing some in-person contact with telephonic or digital communication.8,9 Most weight loss applications (apps) provide a control system10 whose feedback reinforces self-monitoring of diet, physical activity, and weight by visualizing progress toward goals.6,11,12 Patients perceive apps as an acceptable behavior change tool that becomes less predictable and more engaging through the use of passively transmitted worn sensor data.13 Transmitting the participant’s data to a coach extends the control system beyond the individual to a facilitator who tracks progress, conveys accountability, and tailors support provision without requiring a face-to-face meeting.11 | Primary outcomes were weight loss and behavioral adherence. Weight loss was measured both continuously and as the attainment of clinically meaningful ≥5% weight loss.26 The time that coaches spent administering the intervention was an exploratory outcome. Body weight was measured without shoes on a calibrated balance beam scale at baseline, 3-, 6-, and 12-months. Behavioral adherence, operationalized by self-monitoring of diet, physical activity, and weight, was examined during months 1–6. Diet self-monitoring adherence was measured as the percent of days reporting energy intake of ≥ 1000 calories in the paper diary (STND and SELF) or on the ENGAGED smartphone application (TECH). Physical activity monitoring adherence was assessed as the percent of days when any activity was reported in the paper diary (STND and SELF) or when any physical activity was detected on the accelerometer (TECH). Weight self-monitoring was assessed as the percent of days when a body weight was recorded in the paper diaries (STND and SELF) or on the ENGAGED smartphone application (TECH). | Weight loss was greater for TECH and STND than SELF at 6 months [−5.7kg (95% CI: −7.2, −4.1) vs. −2.7kg (95% CI: −5.1, −0.3), p<.05]), but not 12 months. TECH and STND did not differ except that more STND (59%) than TECH (34%) achieved ≥5% weight loss at 6 months (P < 0.05). Self-monitoring adherence was greater in TECH than STND (P <0.001), greater in both interventions than SELF (P <0.001), and covaried with weight loss (r(84) = 0.36 − 0.51, P<.001). Study Participants: At baseline, participants had a mean (SD) age of 39.3 (11.7) years and BMI of 34.6 (3.0) kg/m2. Eighty-four percent were female; 57.3% were white, 31.3% were black. Baseline participant characteristics appear in Table 1. The treatment groups showed no baseline differences on age, sex, race, ethnicity, marital status, education, or weight. Attrition at the final 12 month follow-up assessment was greater for SELF (25.0%) than either STND (12.5%) or TECH (3.1%) treatments (p = .02), and was not differential between the STND and TECH treatments (p=.20) (Figure 1). Treatment Fidelity: Across the study’s duration, 105 coaching calls were assessed for treatment fidelity from 46 participants. Overall, fidelity was 95.0%; three coach re-trainings were held. Weight Loss: At 12 months, participants showed a mean ± 95% CI weight change from baseline of −5.6(−8.5, −2.8) kg in STND, −3.1(−5.9, −0.3) kg in TECH, and −2.7(−5.7, 0.4) kg in SELF (Table 2). When TECH and STND were combined, weight change was significantly greater than SELF at 3 (P < 0.005) and 6 months (P < 0.05), but not 12 months (Table 2 and Figure 3). When measured as a continuous variable, there was no difference in weight change between TECH and STND at any time point. At 6 months, weight loss of at least 5% occurred more often in the experimental treatments (47%) than in SELF (13%) (P < 0.005) and more often in STND (59%) than TECH (34%) (P <0.05). At 12 months, weight loss of at least 5% was observed in 47% of STND, 28% of TECH and 25% of SELF participants; these differences were not significant. Self-Monitoring Adherence: Table 3 compares adherence to diet, physical activity, and weight self-monitoring across the three treatment conditions during the 6 months of the intervention. Diet, activity, and weight self-monitoring were greater in TECH and STND than SELF (P <0.001). Self-monitoring of all behavioral outcomes also was greater in TECH than STND: diet (P <0.05), activity (P<0.001), and weight (P <0.001). The amount of weight loss at 6 months covaried with the amount of self-monitoring of diet [r(84)=.509, p<.001], physical activity [r(84)=.460, p<.001], and weight [r(84)=.364, p<.001]; the correlations did not differ as a function of treatment condition. |
| Boris Hansel et al., 2017  Canada | Other: Fully automated program | 4 months | Patients were asked to log on at least once per week | Online | Fully automated Web-based interventions could more easily be proposed on a large scale compared to telehealth programs that require expensive human support. | After the selection visit, all participants were assessed at baseline(randomization visit) and 4 months later at a final visit - Diet was evaluated by a 3-day dietary recall (two weekdays andone weekend day). This assessment was performed before therandomization visit and during the two weeks preceding thefinal visit. The dates of the 3-day dietary recall were determined randomly in advance by the study team dietician - Body weight was measured to the nearest 0.1 kg, with participants fasting and clothed without shoes, using automated digital scales (TANITA T6360, Tanita Co., Tokyo, Japan). Bodymass index (BMI) was calculated as the ratio between weightand height squared with weight expressed in kilograms andheight expressed in meters.  Total cholesterol, high-density lipoprotein-cholesterol (HDL-C) and triglyceride concentrations were determined by automated enzymatic methods.  Aerobic fitness was determined by maximum oxygen consumption (VO2max), measured by an incremental cardiopulmonary exercise test on a cycle ergometer, with increments of 15-30 Watts/2 minutes | Using an intention-to-treat analysis, the DQI-I score (54.0, SD 5.7 in the ANODE arm; 52.8, SD 6.2 in the control arm; P=.28) increased significantly in the ANODE arm compared to the control arm (+4.55, SD 5.91 vs -1.68, SD 5.18; between arms P<.001). Body weight, waist circumference, and HbA1c changes improved significantly in the intervention. The DQI-I score was 54.0 (SD 5.7) in the e-coaching arm and 52.8 (SD 6.2) in the control arm (P=.28 between arms). The intention-to-treat analysis at 16 weeks showed that e-coaching resulted in a significant improvement in the DQI-I score: +4.55 (SD 5.91) in the intervention arm and-1.68 (SD 5.18) in the control arm (P<.01 between arms; Table2). Moreover, changes in dietary intake tended to differ between arms for lipids (P=.02), saturated fats (P<.01), sodium (P=.07),and empty calories (P=.06), always towards healthier foods in the intervention arm. Compared to usual care, the ANODE program was associated with reduced body weight, waist circumference, and HbA1c(Multimedia Appendix 2). A significantly higher proportion of e-coaching subjects achieved weight loss >3% (20/60, 33.3%of e-coaching subjects vs 4/60, 6.7% of control subjects on intention-to-treat analysis; P<.01) and weight loss >5% (12/60,20.0% vs 2/60, 3.3% on intention-to-treat analysis; P<.01;Figure 3). Only two subjects (2/60, 3.3%) in the ANODE arm and no subject in the control arm achieved >10% weight loss, respectively (P=.15 between arms).No significant differences in terms of change in blood pressure, plasma lipids, amino transferases, gamma glutamyl aminotransferase, uric acid, fasting glucose, VO2max or hs-CRP were observed between the two arms at 4 months |
| Carmen G. et al., 2013  Germany | Virtual navigation - mixed/hybrid - "readily accessible web-based information and support designed to serve as a complement to professional and peer navigation initiatives" | 8 weeks | Not specified, but variable - depends on patient. | Online | Increasingly, individuals are turning to the internet to meet their cancer-related information needs [6,7]. Several surveys have documented that patients prefer receiving information primarily from their health care providers; however, busy clinic visits frequently place limitations on time spent addressing concerns and clarifying issues [8,9]. The internet often becomes the next available solution, but concerns remain about the quality of information retrieved [10]. Checklists have been developed to guide patients in discerning website quality, and new evidence-based, interactive web applications are increasingly being developed [11,12]...Preliminary evidence documents positive health-related outcomes related to interactive web-based tools, also called virtual navigators, and more research is needed to further document their effects [13,14]. | Qualitative research: Thematic content analysis - themes and sub-themes. | Analysis of data revealed three main themes with respective sub-themes depicting participants’ impressions of the OIN™(Fig.2). These included viewing the OIN™ as: Theme 1 - a valuable comprehensive, readily accessible, and reliable source of cancer information and support Sub-themes for Theme 1 1 - Comprehensive 2 - A complement to information received from HCPs 3 - A means of cross-verifying cancer information 4 - Institutionally supported and reliable 5 - Accessible 24/7 Theme 2 - a useful means to control/pace exposure to cancer information Sub-themes for Theme 2 1 - Access to the right amount and type of cancer information 2 - Ability to partition cancer information into manageable chunks 3 - Preference for early introduction Theme 3 - a catalyst for cancer knowledge acquisition and understanding among patients and family members. Sub-themes for Theme 3 1 - Improving patients’ and family members’ knowledge and understanding of cancer 2 - Consequences of improved cancer knowledge Overall: The OIN™ was reported to be instrumental in fulfilling participants’ cancer information and supportive care needs, particularly early in the cancer trajectory. More specifically, the tool was seen as a “go to” resource to obtain more detailed information, validate information provided else-where, and pace exposure to cancer information. Content also was perceived to be of high quality, practical, and comprehensive. All participants underscored how the tool improved their cancer knowledge, facilitated communication, and pre-pared them for subsequent medical consultations. |
| Cassidi D. Kalejta et al., 2019  United States | Other: Automated test reporting | Not specified | Variable | Online | Not specified | Not specified | A total of 10,170 women registered online during the study period, and 8,965 completed the automated process (88%). Out of 8,965 women, 2,121 women responded to the survey (24%). Most (2,030 of 2,101) strongly agreed/agreed that they could easily navigate the patient portal (97%); 1,852 of 1,966 strongly agreed/agreed that disclosure was efficient and convenient (94%); 1,852 of 1,960 strongly agreed/agreed that they felt informed after watching a short educational video (94%); and 1,903 of 1,967 strongly agreed/agreed that they pre‐ ferred downloading results rather than waiting for their next doctor's appointment (97%). |
| Charlene C. et al., 2011  United States | Other: Combination of patient navigation and/through virtual case managers | 1 year | Variable | Other: online, in-person, telephone calls, etc. | In clinical trials, better self-care/lifestyle resulted in better diabetes outcomes (3–5). However, these clinical trials improved outcomes for circumscribed patient populations (6–9). Patients with diabetes are diverse, treatment may involve multiple specialists, and care by primary care providers (PCPs) is limited to 15-min visits. Only 55% of individuals with type 2 diabetes receive diabetes education (10); 16% report adhering to recommended self-management activities. | The primary outcome of the study was change in glycated hemoglobin (%) comparing UC and maximal treatment (CPDS) at baseline versus 12 months. Secondary outcomes: The Patient Health Questionnaire-9(PHQ) was administered at baseline and at follow-up interviews to assess depressive symptoms (15). We used the 9-itemversion of the Self-Completion Patient Outcome Instrument to assess patient-reported symptoms associated with diabetes(16,17) and the 17-item Diabetes Distress Scale (18,19). Clinical measurement related to diabetes complications (blood pressure, lipid levels) was obtained from provider medical office records. Hypoglycemic events, hospitalization, and emergency room visits were ascertained through quarterly telephone calls to patients. Vital status was ascertained through review of physician charts if we could not contact patients. | Although there were mean declines across all groups in lipid values and blood pressure readings, Diabetes Distress, Diabetes Symptoms, andPHQ-9 Depression, none of the 12-monthchanges comparing the UC to any of the active interventions were significantly different (P>0.05)....Hypoglycemic events, hospitalizations, and emergency-room visits were infrequent in all groups. One patient in group4 (CPDS) was hospitalized twice for rea-sons not reported to the study. The DSMB determined that there were no direct study-related adverse events found. No patients died during the 12 months of this study. |
| Charles R. Jonassaint et al., 2017  UK | Other: CCBT and online support groups | Not specified, variable | Not specified, variable | Other: Mix of telephone communication with case managers, email, computerized CBT, and internet support groups | Computerized cognitive–behavioural therapy (CCBT) helps improve mental health outcomes in White populations. However, no studies have examined whether CCBT is acceptable and beneficial for African Americans. | Differences in CCBT use and self-reported change in depression and anxiety symptoms. Engagement measures, mental health outcomes, care manager process measures, pharmacotherapy use. | Compared with White participants, African Americans were less likely to start the CCBT programme (P=0.01), and those who did completed fewer sessions and were less likely to complete the full programme (P=0.03). Despite lower engagement, however, African Americans who started the CCBT programme experienced a greater decrease in self-reported depressive symptoms (estimated 8-session change:−6.6v.−5.5;P=0.06) and similar decrease inanxiety symptoms (−5.3v.−5.6;P=0.80) compared with White participants.  Care manager process measures: The care managers made a median of 14 total contacts (inter-quartile range (IQR) 11–18) to participants. Although the total number of contacts did not differ between African American andWhite people, care managers telephoned African Americans more often than White participants (median (IQR)=5 (3–7) and 3 (2–5),respectively,P<0.001), but sent fewer email messages to African Americans than to White people (median (IQR)=9 (6–11) and 10(7–14), respectively,P<0.001). However, the number of telephone contacts was not associated with change in PHQ (r=0.01;P=0.91)or GAD scores (r=0.05;P=0.31).  Pharmacotherapy use: The three-way interaction between session, race and baseline pharmacotherapy use was non-significant (P=0.7013). Thus, the effect of race (African American v.White) on PHQ-9 decline was not dependent on baseline pharma use. Further, baseline pharmacotherapy use was not a predictor of decline in PHQ-9 (P=0.7877)or GAD-7 (P=0.6713) scores |
| Christine Sawicki et al., 2019  United States | Patient | 365 days | These messages are sent to patients at 3, 6, 9, and 12 months in accordance with recommended safety monitoring—we saw over 40% response rate for these messages...During our pilot study, an average 1.5 messages were sent per month. | Other: 2-way clinical messaging (supported through texts, follow-up calls, emails, additional resources, and health coaching) | Not specified. | Patient demographic and prescription drug utilization data were obtained from the CVS Specialty claims database. One-way texting and clinical messaging enrollment data were obtained from messaging program enrollment files and the pharmacy patient profile....Medication adherence was calculated as the medication possession ratio (MPR) during the 365 days of follow-up: total days supply÷365 days×100, truncated at 100%. Optimally adherent patients were defined as having had an MPR of >85%. This threshold is based on studies demonstrating differences in cytogenic response rates and mean molecular responses between patients with CML at and below 85% MPR.11 Adherence drivers recorded and analyzed included length of therapy (time between initiating therapy and the exhaust date of the last fill); first fill drop-off (discontinuation of therapy after 1 fill and no further fills during the follow-up period); and gap days between refills (among patients with at least 2 prescription fills, average gap days were summed between exhaust dates of previous fills and next fill dates, divided by the number of refills). Medication persistency was measured as a secondary out-come. A gap in medication persistency was defined as a period of >60-day gap between fills (switching among TKI therapies was allowed here, so that patients who switched were not included as non persistent). To examine persistency variances between first- and second-line therapies, we looked at persistence by drug. | Patients receiving clinical messaging had on average a 7.64% higher MPR score (MPR: 73.94% vs. 66.30%) compared with the control arm (P=0.0063). This translates to 22% more patients being optimally adherent while exposed to clinical messaging (P=0.022). Patients in the exposed group had a mean 32-day increase in average length of therapy compared with the control group (243 days vs. 275 days, P=0.0043), potentially driving an increase in adherence. Additional drivers included a 5.4 percentage point reduction in first fill drop-off rates (4.66% vs. 10.04%, P=0.0149). Persistency after 12 months was similar between the study arms (41%).  After 1:1 propensity score matching, 279 patients were assigned in each group, which included 81% of the population. Imatinib (n=238) and dasatinib (n=212) were the most frequently used agents, followed by nilotinib (n=70) and bosutinib (n=12). Baseline characteristics were balanced between the groups (Table 1). For example, gender (52.0% male, 54.8% male, P=0.50) and age at first fill (53.3 years, 54.4 years, P=0.35) were similar between the matched intervention and control groups...Overall, enrollment and participation in clinical messaging was associated with improved adherence to medication. MPR for participants in clinical messaging was 73.9%, which was 7.6 percentage points higher than the control group’s MPR of 66.3% (P=0.01; Table 2). At the drug level, the dasatinib group was the only one to achieve statistically significant differences between the compared arms with a difference of 10.5% (P=0.01). Of the clinical messaging patients, 53.4% were optimally adherent, compared with 43.7% in the control group, a difference of 9.7 percentage points (P=0.02). Clinical messaging patients were 22% more likely to be optimally adherent. The number needed to treat for an additional patient to be optimally adherent was 17 (data not shown)...Among all patients, the average length of therapy was 243 days for the control group and 275 days for the intervention group, an increase of 32 days (P=0.01; Table 2). The first fill drop-off rate was 10.0% in the control group versus 4.7% in the exposed group, a difference of 5.4 percentage points (P=0.02). Average gap days for the clinical messaging and control groups were similar. Persistency was similar between first- and second-line drugs, with yet only 41% of patients remaining on therapy after 12 months (Figure 1). Compared with the control group, intervention group patients were more likely to persist on first-line therapy (hazard ratio [HR]=1.2, 95% confidence interval [CI]=0.82-1.74; Figure 2). The effect was similar for second-line therapies, with intervention patients 1.8 (95% CI=0.75-4.35) times as likely to persist on therapy compared with controls (Figure 3). |
| Christophe JP Smeets et al., 2018  Canada | Other: Remote monitoring/coaching | Observed for 6 months | Monitored on a daily basis | Online | Not specified | Outcome measures included CardioCoach user experience, (therapeutic) adherence, call center statistics, algorithm performance, and the number of patients on guideline-recommended medication dose for β-blocker and ACE-I (Table 2) at both 3 and 6 months of follow-up. | Patients’ satisfaction and adherence for medication intake (10,018/10,825, 92.55%) and vital sign measurements (4504/4758, 94.66%) were excellent. However, the number of technical issues that arose was large, with 831 phone contacts (median 41, IQR 32-65) in total. The semiautomatic remote uptitration was safe, as there were no adverse events and no false positive uptitration proposals. Although no significant differences were found between both groups, a higher number of patients were on guideline-recommended medication dose in both groups compared with previous reports.  On the basis of gathered data, the CardioCoach algorithm generated 72 medication uptitration proposals in total. In 7% (5/72) of the cases, the algorithm generated a conclusive proposal, whereas in 93% (67/72) of cases, the decision was left up to the HF nurse. This was mainly due to aberrant (67%, 48/72) or incomplete (25%, 18/72) data.  Overall, therapeutic adherence as confirmed by the patient via the smartphone app (8315/10,825, 76.81%) or via the technical call center after contacting the patient (1703/10,825, 15.73%) for the 3 drug treatments was 92.55% (10,018/10,825), with, respectively, 97.12% (3239/3335) for β-blockers, 94.89% (3549/3740) for ACE-I, and 86.13% (3230/3750) for diuretics. |
| Chul Hyun et al., 2020  United States | Patient | 1 year | Variable | Online | Chronic hepatitis B virus (HBV) infection is a major cause of liver-related morbidity and mortality among Asian Americans in the United States. Despite the available resources, a majority of HBV-infected individuals are not able to access adequate health care owing to numerous barriers. | The frequency of text messaging to and from the patient navigators was recorded. The contents of the messages were then analyzed by classifying them under 1 of the 4 following thematic categories: medical access (finding physicians or health facilities), reminders and schedules, financial costs and insurance, and health information and education.  Outcome in linkage to care (LTC) was evaluated at the end of 6 months. In the nonimmune group, the participants who received at least two hepatitis B vaccinations were considered linked. In the CHB group, the participants who saw a physician at least once for further evaluation of their CHB status were also considered linked to care. | On average, patient navigators sent and received 14 and 8 messages per participant, respectively, during the 6-month period. The themes of the messages were similar to the following 4 categories: finding providers, scheduling appointments with providers, health education, and financial issues. Of the 82 participants, 78 were linked to care within 6 months (a 95% linkage rate). |
| David Ebert et al., 2013  Switzerland | Other: Hybrid/mixed - involving both peer and patient navigation components | 12 weeks | Variable | Hybrid | High risk of relapse...continuation of therapy is required, but not feasible for various reasons...The use of the Internet to provide guided self-help may represent a cost- effective, far-reaching method for implementing continuation phase treatments. This method has been well accepted by patients and shown to be efficient in the acute treatment of a variety of disorders [11–15] . The adaptation of Internet-based strategies for the continuation/maintenance phase has several advantages. These include: (a) greater potential for the integration of acquired skills in daily life due to an emphasis on the patient’s active role in self-help treatment, (b) elimination of waiting periods between acute and continuation treatment, (c) access to the programs on a 24/7 basis, and (d) lower costs compared to face-to-face treatments. Several studies have shown promising results with delivering continuation phase treatments over the Internet [16–21] . However, to our knowledge, all published studies evaluating the use of this approach to maintain inpatient treatment gains [16, 17, 20] used only nonrandomized designs. | The primary outcome was general psychopathological symptom severity (GPS). Secondary outcomes were psychological well-being, depressive symptoms, somatoform complaints, phobic anxiety, interpersonal difficulties, self-efficacy, positive and negative affect, and emotion regulation skills. All measures except positive affect, negative affect and emotion regulation skills were assessed using the corresponding subscales of the HEALTH-49 [35]....Positive and negative affect were assessed with the German version of the Positive and Negative Affect Schedule [39]...Emotion regulation skills were assessed with the Emotion Regulation Skills Questionnaire [40] | The TIMT + TAU group was superior to the TAU-only group with regard to differences in change of general psychopathological symptom severity from discharge to 3- and 12-month follow-up. Moreover, participants of the TIMT + TAU group showed less frequent symptom deteriorations and were more often in remission/recovery than controls. |
| Dayana M et al., 2019  Ireland | Patient | N/A (in construction and validation phase). | N/A (in construction and validation phase). | Online | These contents were approached using the Health Belief Model, which aims to develop knowledge in the target audience regarding the susceptibility to the occurrence of UI, its severity and the benefits of decision making, motivating them to adhere to the program proposed in the application (Table 1). | N/A | Most of health experts were physiotherapists (n = 07) and professionals with a PhD (n = 07). Regarding the CVI, 100% of the evaluated items obtained values of 0.86 or greater. Most of information technology/computing/communication experts were male (81.8%) and of the information technology area (63.6%). Except for “restart sessions”, “ways of presenting suggestions”, “user interaction” and “motivates questioning”, all the other aspects received between 81.8% and 100% positive responses. All items evaluated by the target audience obtained a minimum of 94.3% positive responses from the participants. In this step, points for adjustment were identified in relation to the application content and interface, which were promptly corrected...Conclusion: The application has been validated for use in clinical practice as an educational technology to promote adherence to pelvic floor muscle training and prevention of urinary incontinence in postpartum women. |
| Deanna Kerrigan et al., 2019  United States | Other: Included SMS reminders | 18 months | Variable | Online | Community empowerment approaches that seek to address sociostructural constraints associated with the higher risk of HIV infection experienced by FSW have been shown to be effective, and cost-effective, in this key population.Such approaches are set within a broader health and human rights framework and are tailored to the needs and priorities of a given community | Primary study outcomes assessed after 18 months were HIV acquisition among participants who were HIV-negative at baseline and viral suppression (,400 copies/mL) among those who were HIV-positive at baseline.  Secondary prevention outcomes included HIV risk behaviors such as inconsistent condom use with new and regular clients. Secondary care and treatment outcomes include engagement in HIV care, ART use, and adherence, which was assessed using the AIDS Clinical Trials Group measure for reported adherence in the last 4 days. | The analysis included 171 HIV-positive and 216 HIV- negative FSW who completed baseline and 18-month study visits. Participants in the intervention were significantly less likely to become infected with HIV at 18-month follow-up (RR 0.38; P = 0.047), with an HIV incidence of 5.0% in the intervention vs. 10.4% control. Decreases in inconsistent condom use over time were significantly greater in the intervention (72.0%–43.6%) vs. control (68.8%–54.0%; RR 0.81, P = 0.042). At follow-up, we observed significant differences in behavioral HIV care continuum outcomes, and positive, but nonsignificant, increases in viral suppression (40.0%–50.6%) in the intervention vs. control (35.9%–47.4%). There was a strong association of between higher intervention exposure and HIV outcomes including viral suppression. |
| Deborah Morrison et al., 2015  UK | Other: Online resource | Not specified, Variable | Not specified, Variable. Email reminders are sent every 2 months to visit the website/self-assess, or contact dr if necessary | Online | Supporting optimum self-management by providing relevant self-management education including how to use an asthma action plan (AAP), regular health professional review, and optimal use of medications has been shown to have positive effects on a range of asthma outcomes such as improved quality of life, lower rates of healthcare con- tacts, and fewer days off work and school. | N/A | The website asked users to aim to be symptom free. Key behaviours targeted to achieve this include: optimising medication use (including inhaler technique); attending primary care asthma reviews; using asthma action plans; increasing physical activity levels; and stopping smoking. The website had 11 sections, plus email reminders, which promoted these behaviours. Feedback on the contents of the resource was mainly positive with most changes focussing on clarification of language, order of pages and usability issues mainly relating to navigation difficulties. |
| Donna L Berry et al., 2014  UK | Other: Web-based/electronic coaching or patient education. | About 6 weeks | Not specified, but variable. | Online | Patient-clinician communication has been evaluated and found lacking with regard to clinician assessment of patient experiences, notably symptoms and quality-of-life is-sues (SxQOL) [1-3], and verbal patient reports of SxQOL[4]. Barriers to communication in the oncology setting have been identified and include 1) clinician-oriented verbal behaviors: use of close-ended (versus open-ended)queries and interruptions of patient symptom descriptions[5,6], changing the subject after a patient verbally reports an SxQOL; [7] 2) clinician beliefs that quality of life is-sues are other clinicians' responsibility [8], 3) patient-oriented issues: reluctance to verbalize problems [9], re-call of SxQOL experiences in between visits [10], and 4)time limitations during the visit [11]. When clinicians are unaware of SxQOL, particularly treatment-related toxicities, there is danger of higher morbidity and even mortality related to unintentional over-dosing [12,13]. Interventions to improve patient-clinician communication have been tested with modest, but positive, results [9,14-16]. | Audio-recordings of clinic visits made 6 weeks after treatment initiation were coded for discussions of 26 SxQOL issues, focusing on patients’/caregivers’ coached verbal reports of SxQOL severity, pattern, alleviating/aggravating factors and requests for help. Among issues identified as problematic, two measures were defined for each patient: the percent SxQOL reported that included a coached statement, and an index of verbalized coached statements per SxQOL. The Wilcoxon rank test was used to compare measures between groups. Clinician responses to problematic SxQOL were compared. A mediation analysis was conducted, exploring the effect of verbal reports on SD outcomes. | Patients with audio-recordings were younger in the intervention group than in the control group (p < .0001). Out of 517 patients, 27 (13 control and 14 intervention) did not discuss any problematic SxQOL issue during the clinic visits. There was no significant difference (p = 0.41) between study groups in number of problematic SxQOL issues discussed at all during clinic visits, with a median of 4 issues discussed by control group patients, and 3 by patients in the intervention condition. Patients initiated general discussion of an average 56% of problematic SxQOL issues in the control group and 55% in the intervention group (p =0.97). Family members initiated 4% of the problematic SxQOL issues in the control group and 5% in the intervention group (p = 0.35)...The percentage of problematic SxQOL issues which patients or caregivers reported using any specific coached statement during the clinic visit, was significantly higher (p = 0.002) in the intervention group than that in the control: a median of 85% of problematic SxQOL were reported as coached in the intervention group versus 75% in the control (Table 2). After adjusting for covariates, group remained significantly associated (p = 0.0009); intervention group patients had an approximate 9% higher rate of describing problems with a coached statement (Table 3). Discussion Section: The patients in the ESRA-C II randomized trial who received an educational coaching intervention to aid verbal report of problematic SxQOL applied the reporting framework as coached (severity, pattern, aggravating/alleviating factors and help request), reporting these specific details without prompting, significantly more often than control group patients. When examining individual SxQOL issues, we found that reports for the majority of individual SxQOL issues were more frequent in the intervention group. Even though our study was not powered to compare individual SxQOL issues, we found that fatigue, pain and physical function were reported significantly more often by the intervention group. Conclusion Section: Electronic education and coaching provided to patients with a variety of cancers of all stages resulted in significantly more specific verbal reports of SxQOL concerns made to treating clinicians in face to face visits. Results from Abstract (may be duplicate): 517 (256 intervention) clinic visits were audio-recorded. General discussion of problematic SxQOL was similar in both groups. Control group patients reported a median 75% of problematic SxQOL using any specific coached statement compared to a median 85% in the intervention group (p = .0009). The median report index of coached statements was 0.25 for the control group and 0.31 for the intervention group (p = 0.008). Fatigue, pain and physical function issues were reported significantly more often in the intervention group (all p < .05). Clinicians' verbalized responses did not differ between groups. Patients' verbal reports did not mediate final SD outcomes (p = .41). |
| Donna M. Zulman et al., 2015  United States | Other: No specific intervention was included - study assesses general eHealth technology - this could included hybrid/combination of peer and patient navigation. | N/A. No specific intervention was included - study assesses general eHealth technology. | N/A. No specific intervention was included - study assesses general eHealth technology. | Online | The presence of multiple chronic conditions (MCCs) is associated with poor clinical outcomes...These outcomes are in part a function of substantial self-management and coordination challenges that arise when patients have multiple health issues...As a result, there is a great need for effective tools to support the self-care activities associated with multiple health issues. One source of potential disease self-management and health care navigation support is eHealth technology...Despite the growing availability of eHealth technology, it is unclear whether existing tools are meeting the needs of patients with high levels of illness burden. | N/A, but focus group sessions were transcribed and analyzed using standard content analysis methods for coding textual data. | Focus group themes: Analysis of focus group transcripts yielded three primary themes encompassing many of the challenges that patients face when managing MCCs: (1) Managing a high volume of information, self-management tasks, and communication; (2) coordinating, synthesizing, and reconciling information from different providers and about different conditions; (3) needing to serve as their own expert and advocate because of their unique combination of health issues. Across themes, many patients expressed emotional distress (e.g., stress, frustration) with their current ability to self-manage and coordinate care. Participants identified a number of opportunities to advance eHealth technology to better meet their needs |
| Elizabeth L. Clemens et al., 2018  United States | Other: Smartphone technology for self-monitoring BP with patient navigation (telephone/in-person). | 9 month study period | At the study site, patients took the BP monitors home and were asked to take their BP (using a smartphone device) three to seven times per week, for the 9-month study period, at the same time each day (1 h after medications and/or 1 h after eating). | Hybrid | Home blood pressure monitoring (HBPM) has a well-established primary role in diagnosis, treatment adjustment, and follow-up for patients with hypertension (HTN), and current guidelines recommend its wide use in clinical practice. There are many types of HBPM, including those that use wireless technology that link wireless sensors through radio signals to smartphones and computers. Wireless technology is a promising and innovativemethod for managing healthcare in patients with chronicillness. Until 2010, no wireless connectivity technology met all the requirements needed for widespread adoption, that is, interoperability, low-power operation, customized software, compatibility, transmission, and sensors needed to communicate with services. Bluetooth low energy technology is an innovative technology that meets all these requirements. Today, wireless technology has become more affordable and acceptable, especially in low-resource settings, making it an opportune time to adopt smart technology to better engage patients in the self-management of chronic diseases.However, challenges to the adoption of any technology include the rapidly changing nature of technology development and the cost of new technology. | The proportion of patients with controlled BP was compared between groups at pre- and postintervention, ~9 months later. Participant's blood pressure levels. Cases and matched controls were compared on pre- and postintervention rates of BP control at the four clinics combined. Patient-level outcomes included measured improvements in the proportion of patients with BP considered ‘‘controlled,’’ defined as <140/90 for patients aged <60 or <150/90 for patients more than/equal to 60; patient satisfaction with the device, the smart-phone application, the data, and their relationship with their provider; and patient’s sense of well-being. Secondary: Chart reviews were conducted on all patients at the study site to determine providers’ actions based on information obtained from the wireless BP monitors, specifically elevated readings identified by the study nurse navigator. Secondary: Patient compliance for using application. Additional info: Patient-level outcomes included measured improvements in the proportion of patients with BP considered ‘‘controlled,’’defined as<140/90 for patients aged<60 or<150/90 for patients equal to or above 60; patient satisfaction with the device, the smart-phone application, the data, and their relationship with their provider; and patient’s sense of well-being. Chart reviews were conducted on all patients at the study site to determine providers’ actions based on information obtained from the wireless BP monitors, specifically elevated readings identified by the study nurse navigator. Thirty-seven patient charts were reviewed to determine if medication changes were made during the study period. | Use of the smartphone BP monitor was found to be an independent predictor of improved BP control when controlling for age, baseline diabetes diagnosis, and baseline BP control status  Thirty-seven patients with 17 unique providers, representing 3 clinic sites, participated at the study site; 94 additional patients were enrolled in parallel quality improvement projects at the other 3 clinics, followed similar protocols, and used the identical device. A total of 353 matched control patients were included for comparison. |
| Elyse R. Park et al., 2020  United States | Not specified | 8 sessions (8 weeks) | Weekly 1.5 hr sessions | Hybrid | The SMART- 3RP is guided by the diathesis-stress model, which posits that resilience is the outcome of an individual’s experiences and environment in combination with one’s inherent coping ability | Phase 1: Psychosocial needs of parents of children with learning disabilities Phase 2: Measures were collected on intervention constructs within the stress management and growth enhancement processes. | Fifty-three parents (mean age = 46.8; 90.6% female) participated nationally in the pilot trial. 62.5% of participants completed ≥ 6/8 sessions; 81.8% reported continued daily/weekly relaxation response exercise practice. T1–T2 comparisons found that IG versus WC participants showed sig- nificant improvements in distress [VAS], ∆M = − 1.95; d = .83 and resilience [CES], ∆M = 6.38; d = .83, as well as stress coping [MOCS-A] ∆M = 8.69; d = 1.39; depression and anxiety [PHQ-4], ∆M = − 1.79; d = .71; social support [MOS-SSS], ∆M = 5.47; d = .71; and empathy [IRI], ∆M = 3.17; d = .77; improvements were sustained at the 3 month post intervention follow-up.  Participants (n = 53) attended a median of 6 out of 8 ses- sions; 62.5% of participants completed ≥6/8 sessions, and participation rates were similar across both groups. Among enrolled participants, 40 (75.5%) completed the time 2 sur- vey and 35 (66.0%) completed the time 3 survey. The larg- est proportion of dropout (38.7%) occurred in the interven- tion group condition between time 1 and time 2, with one group conducted in the late spring which overlapped with the end of the school year. Study completers (n = 40) and non-completers (n = 13) did not differ by demographic factors assessed at enrollment. |
| Emily S Ross et al., 2021  Canada | Patient | 60 days | Participants in the Txt2Prevent group received 48 unique, automated, one-way messages over 60 days following randomization in addition to usual care. An additional 4 messages relating to study administration (eg, indicating the end of the study and requesting participants to inform us if they were readmitted; see SMS text messages sent on days 7, 26, 45, and 60 in Multimedia Appendix 1) were also sent during the study period. The SMS text messages were delivered at a time of day specified by the participant, began after the participant was randomized, and were sent daily for the first 36 days and then every other day until day 60. | Other: Text-messaging | Home-based programs, often nurse led, can improve quality of life and reduce readmissions [18,19], but these face-to-face interventions can be a challenge for strained health care systems. The widespread use of information and communication technology, such as mobile phones, may be an easier and more convenient way to reach patients. SMS text messages are an attractive technology, as over 90% of adults aged 65 years or older own a cell phone [20], and 80% of cell phone owners currently text [21]. SMS text messages also have the benefits of being able to store messages that can be reaccessed, have a wide geographic reach, are convenient due to the asynchronous nature of communication, and have low delivery costs. Previous SMS text messaging studies in patients with or at risk for cardiovascular disease (CVD) have reported improvements in self-management behaviors (eg, medication adherence [22] and increases in leisure physical activity and walking [23]) and cardiac risk factors (eg, lowering low-density lipoprotein cholesterol and systolic blood pressure [24,25]). These studies show the promise of using SMS text messaging to aid in the care of patients with CVD. However, they do not specifically target the multiple self-management behaviors required in the immediate period after discharge using only SMS text messages. | The primary outcome was measured with the Health Education Impact Questionnaire (heiQ). Other outcomes included the EQ-5D-5L, EQ-5D-5L Visual Analog Scale, a modified Sullivan Cardiac Self-Efficacy Scale, and Morisky Medication Adherence Scale scores, and self-reported health care resource use. Analyses of covariance were used to test the effect of group assignment on follow-up scores (controlling for baseline) and were considered exploratory in nature. Feasibility was assessed with descriptive characteristics of the study protocol. Acceptability was assessed with 2 survey questions and semistructured interviews. | For the secondary outcomes, there were no statistically significant differences in adjusted analyses except in 1 self-efficacy domain (Total plus), where the Txt2Prevent group had lower scores (mean difference –0.36, 95% CI –0.66 to –0.50, P=.03). The study protocol was feasible, but recruitment took longer than expected. Over 90% (29/31 [94%]) of participants reported they were satisfied with the program. |
| Estelle Everett et al., 2018  Canada | Not specified | Not specified, variable | Not specified, variable | Online | Sweetch’s core philosophy is that each individual has his or her own life habits, motivations, and pace of behavioral-change progress  Behavioural change theory | Feasibility was assessed by study retention. Acceptability of the mobile platform and DBWS were evaluated using validated questionnaires. Effectiveness measures included change in PA, weight, BMI, glycated hemoglobin (HbA1c), and fasting blood glucose from baseline to 3-month visit. The significance of changes in outcome measures was evaluated using paired t test or Wilcoxon matched pairs test. | The study retention rate was 47 out of 55 (86%) participants. There was a high degree of acceptability of the Sweetch app, with a median (interquartile range [IQR]) score of 78% (73%-80%) out of 100% on the validated System Usability Scale. Satisfaction regarding the DBWS was also high, with median (IQR) score of 93% (83%-100%). PA increased by 2.8 metabolic equivalent of task (MET)–hours per week (SD 6.8; P=.02), with mean weight loss of 1.6 kg (SD 2.5; P<.001) from baseline. The median change in A1c was −0.1% (IQR −0.2% to 0.1%; P=.04), with no significant change in fasting blood glucose (−1 mg/dL; P=.59). There were no adverse events reported.  After excluding 35 participants who had either normal glucose tolerance or diabetes mellitus, 57 participants were eligible for the study, of whom 55 (96%) enrolled. Of the 55 enrolled participants, 12 (22%) and 43 (78%) were in the calibration and intervention cohorts, respectively. Of the 12 participants in the calibration cohort, 9 (75%) completed the study according to protocol. One participant was dropped because he was logged out of the app for a period longer than 14 days, and 2 were dropped because of protocol violations (one participant failed to return for final visit, and another’s final visit was outside the study window). In the intervention cohort, 38 out of the 43 (88%) participants completed the study. Reasons for dropping out in this cohort included 3 participants that logged out or removed the app, one who voluntarily dropped out, and one protocol violation (torn knee meniscus that limited ambulation; notably, this participant did not attribute the knee injury to increased PA). Among the 55 enrolled participants, 14 (25%) received the Sweetch app alone, and 41 (75%) received the app + DBWS  With respect to feasibility measures, there was relatively high retention in the study, with 47 of 55 (86%) participants completing the study according to protocol. The median (IQR) time between the baseline and follow-up visits for study completers was 91 (90-98) days. The Sweetch mobile platform had a high degree of acceptability by the participants. The median (IQR) score for the Sweetch app SUS measure was 78% (73%-80%), with a score above 68% indicating above average acceptability. There was also high satisfaction with the DBWS with a median (IQR) usability score of 93% (83%-100%).  With respect to the effectiveness measures (Table 2), there was a significant increase in PA from baseline with a mean change of 2.8 MET-hours per week (SD 6.8; P=.02). Weight reduction of 1.6 kg (SD 2.5; P<.001) was observed, corresponding to weight change of approximately 2%. BMI declined by 0.6 kg/m2 (SD 0.8; P<.001), and waist circumference was reduced by 1.4 cm (SD 2.9; P<.01). There was no significant change in blood pressure with median (IQR) change of 1 mm Hg (−10 to −7; P=.56) and −4 mm Hg (−7 to 4; P=.21) in systolic and diastolic blood pressure, respectively. |
| Eun-Shim Nahm et al., 2019  United States | Other: Nurse navigator sending and monitoring e-messages | Variable | Six bi-weekly scripted messages, Variable deopending on response and support required | Online | As shown in previous studies, SCPs may yield better outcomes when delivered with necessary support.  Content of the WBC program was guided by a conceptual quality of life model for survivors | At the end of the intervention, participants were asked about their perceptions of the usefulness of SCPs. and WBC. The former was assessed using one item with a yes/no response option; the latter was assessed using a three-item usefulness sub scale of the Health Web Site Usability Questionnaire on a seven-point Likest-type scale from 1 (strongly disagree) to 7 (strongly agree). In addition, experiences with using the WBC program and SCPs were assessed using open-ended questions.  - Health related quality of life was assessed using the 12-item SF-12 that asks about a persons mental health - Symptom burden was assessed using the Memorial Symptom Assessment Scale - Impact of cancer on the lives of survivors was assessed by the 47-item impact of cancer scale - Fear of recurrence was assessed by the six-item Assessment of Survivor Concerns scale on a four-point Likest-type scale - Levels of physical activity were assessed using the International Physical Activity Questionnaire - Short form - Levels of dietary behaviours were assessed using the Combined Fat/Fruit-Vegetable screener - Patient-provider communication was assessed using the three areas of Components of Primary Care Index - Adherence to treatment was assessed using the five-item Medical Outcomes Study General Adherence Scale on a six-point Likest-Type scale E-health literacy was assessed by eHealth Literacy Scale | At three months, there was a significant improvement in quality of life, physical symptom burden, and total symptom burden. |
| Eva Haukeland Fredriksen et al., 2016  Canada | Other: peer-to-peer support groups | Engagement with discussion forms was variable | Engagement with discussion forms was variable | Online | Nutbeam and Renkert introduced the concept of “maternal health literacy" | Not specified | From abstract: In our study sample, The interaction in Web-based discussion forums influenced maternal health literacy in terms of increased health-related knowledge and competencies, increased awareness of health promotion and health protection, and increased system navigation. The women appraised and selectively applied information and advice that resonated with their own experiences. For many, the information provided online by other women in the same situation was valued more highly than advice from health professionals. Women reported that they used their knowledge and competency in encounters with health professionals but hesitated to disclose the origin of their knowledge. Those with a high level of education in medicine-related fields raised a concern about the Internet as a source of horror stories and erroneous information and were actively engaged in trying to minimize potential negative effects, by providing biomedical information. |
| Francisco Monteiro-Guerra et al., 2020  Canada | Not specified | Not specified | Not specified | Other: App | To guide the design process, we used applicable behavior change theory, with the aim of increasing the long-term effectiveness of the PA system. Appropriate theoretical frameworks (self-determination theory [SDT] and social cognitive theory [SCT] )and constructs were identified from related work and empirical evidence to highlight factors, barriers, and determinants that brought important insights into the design of the solution. Personalization theory was also explored in the tool design, which may help increase the intended effects of the app communication and, in that way, increase the effectiveness of the behavior change intervention | Not specified | The design process has led to the conceptualization of a personalized coaching app for walking activities that addresses the needs of breast cancer survivors. The main features of the tool include a training plan and schedule, adaptive goal setting, real-time feedback and motivation during walking sessions, activity status through the day, activity history, weekly summary reports, and activity challenges. The system was designed to measure users’ cadence during walking, use this measure to infer their training zone, and provide real-time coaching to control the intensity of the walking sessions. The outcomes from user testing and expert evaluation of the digital prototype were very positive, with scores from the system usability scale, mobile app rating scale, and app behavior change scale of 95 out of 100, 4.6 out of 5, and 15 out of 21, respectively. |
| Harm L. Ormel et al., 2018  Germany | Other: self-monitoring | Not specified | Not specified, variable | Other: App | Evidence accumulates that life- style influences cancer treatment outcome, and changes the recurrence rates of the disease. Several studies indicate that weight gain after cancer treatment increases the risk of cancer recurrence and cancer-related mortality | Primary aim of this study was to determine the feasibility of the RunKeeper app  The secondary aim was to explore the usability and patient  Changes in PA were determined with the Physical Activity Scale for the Elderly (PASE) at baseline (T0), 6 weeks (T1), and 12 weeks (T2). Usability and patients’ experiences were tested at T2 with the System Usability Scale (SUS) and a semi-structured interview. | In total, 78 patients were assessed for eligibility by screening the medical records of patients visiting the Medical Oncology department of the UMCG. Nine patients did not have a smartphone, one patient was participating in oncologic reha- bilitation and one was an active user of the RunKeeper app (see Fig. 1). Seven were ineligible due to medical reasons. Of the 60 eligible patients, 28 declined (too busy n = 4, already active n =5, no reply n =4 and not interested n =15) and 32 agreed to participate. Patients were randomized in Group A, intervention group (n = 16) or Group B (n = 16), control group. No adverse events related to the study intervention occurred.  4 qualita- tive semi-structured interviews were performed (see Supplementary Table S3). Two patients did not respond on our telephone calls and did not participate in this part. Most patients (n = 12) were enthusiastic about the RunKeeper app use. Eleven patients were still frequently using the RunKeeper app to self-monitor PA at T2. |
| Heewon Kim et al., 2020  UK | Other: Coaching | 16 weeks | Variable | Online | Not specified | Not specified | Our analysis revealed that coaches employed various patient- centered strategies to assist users in enacting sustainable behavior change, ranging from small-scale changes in daily routines to long-term goal establishment. Coaches’ primary PCC strategies included: (a) triggering reflections on users’ routinized habits, (b) jointly determining a measurable health goal, (c) facilitating self- evaluations on recent behavior change, and (d) tailoring programs to adapt to users’ lifestyle or health status. Notably, coaches’ communication patterns indicated that coaches had built and maintained a high level of situational understanding of users’ daily routines and behavior change by utilizing a variety of in- app features and maintaining constant contacts with users through in-app chat. In particular, coaches substantively benefited from referring to users’ electronic health records archived in the application while implementing the aforementioned PCC strate- gies. In this section, we will delineate the ways in which these four PCC strategies were enacted in mHealth-based coaching, specifi- cally paying attention to the nature and affordances of mHealth technologies that may shape the patterns of PCC. |
| Hong Xiao Sara R. et al., 2015  United States | Patient | Not specified clearly. | Not specified clearly. | Other: Email intervention and telephone (voice) message intervention | In 2012, 86 million Americans aged 20 years or older had prediabetes (1). Studies suggest that people with prediabetes have a high risk for developing type 2 diabetes in 5 years if they do not receive appropriate prevention interventions (2,3)....The Diabetes Prevention Program study reported that a 58% reduction in incidence of type 2 diabetes was observed among adults with prediabetes during the 3-year follow-up as a result of lifestyle interventions to improve diet, increase physical activity, and encourage weight loss(4). Effective lifestyle interventions help prevent type 2 diabetes from developing among people with prediabetes (4,5)...Health coaching, often delivered by non physician health care providers, enhances patients’ commitment to lifestyle change via evidence-based behavioral change techniques, such as motivation-al interviewing (6–8). Studies suggest that health coaching im-proves compliance and outcomes for patients with chronic dis-eases (7,9) and reduces medical costs and hospitalizations (10).These types of health and wellness coaching services may be even more effective when fully integrated into a person’s overall healthcare delivery system (11). | Our study’s main outcome measure was the uptake rate for WCC participation, which was measured by whether patients made an appointment with the WCC within 6 weeks after they were contacted via any one of study intervention methods (secure email message, IVR telephone message, letter). We also assessed whether those in the usual care arm (who received no study contact) made a WCC appointment.  Initial descriptive statistical analyses were conducted for the out-come (the uptake of WCC) and independent variables, which were age, sex, race/ethnicity, fasting plasma glucose level, body mass index (BMI) (kg/m2), and primary care visit counts at baseline....We designed the study to have80% power to evaluate the effectiveness of each arm, stratified by secure-message eligibility and with no multiple hypotheses testing, to detect a significant difference at the P < .05 level. | Each intervention arm had a higher uptake rate than the usual-care arm. Among secure-message-eligible members, the secured email message had higher uptake rate than the letter, which had a higher uptake rate than the IVR telephone message (P <.05 for all pair-wise comparisons). Among secure-message–ineligible members, the letter also had a higher uptake rate than the IVR telephone message. Patient age was associated with uptake of the WCC program. For each additional year of age, the estimated odds of making an appointment increased significantly (OR = 1.02; 95% CI, 1.01–1.04).Women were nearly twice likely to make an appointment than men (OR = 1.87; 95% CI, 1.40–2.51). There were no significant differences in uptake between non-Hispanic white patients and African American, Asian, Hispanic, or Native American patients |
| Ingrid C Cnossen et al., 2014  Canada | Patient | 6 weeks | Patients were invited to perform HM at home, at least once a day...for 15-minute long exercises. | Other: 1. HM comprises one face-to-face pretreatment counseling session, on the first day of (C)RT - patient education  2. HM is available in three different formats....Both the online format and booklet format offer general information about HNC and its treatment, with written descriptions of the exercises, and with photo and video examples of the exercises either offered online [40] or by means of a 15-minute instructional DVD. The third format consists of a 2-paged A4 leaflet that offers only a written description of the exercises.  3. Weekly coaching was provided by a speech and swallowing therapist - offered as 10-minute sessions by phone or email. | Acute side effects of radiation result in a significant symptom burden and interfere with normal physiologic functions and daily activities... Pretreatment counseling by a speech and swallowing therapist (ST) could be provided to inform the patient and family...Patients should, for example, be informed about the importance of continuing to swallow throughout their courses of (chemo)radiation ([C]RT), because inactivity of the swallowing muscles may lead to disuse atrophy...Research is, however, still in an early stage and much is unknown [35,36]. As a result, not all patients with HNC undergoing (C)RT are prescribed a standardized exercise program as a preventive measure [37]...urgent need for an easily accessible prophylactic education and exercise program, countering the radiation fibrosis, and safeguarding patients against additional consults with health care professionals during (C)RT. A multimodal self-help program is expected to enhance reach by overcoming logistical and financial barriers both on the part of health care providers and patients [38,39]. To our knowledge, there are no self-help programs offered with remote coaching, targeting prevention of deterioration of speech, swallowing, and shoulder function. | Demographic info: (ie, gender, age) and clinical (ie, tumor subsite and stage, treatment modality) information of participating patients was extracted from the hospital information system.  Uptake of HM: addressed how many patients were willing to start HM during (C)RT (uptake percentage).  Adherence: concerned the degree to which HNC patients followed HM at least once a day during 6 weeks of (C)RT and was assessed in two ways: (1) patient-completed diaries, and (2) percentage of patients who started and kept up exercising for 6 weeks.  Exercise performance level: using patient-completed diaries...low level of exercise performance consisted of an exercise performance of all exercise categories during 6 weeks at most once a day on average (range 0-168)...moderate level consisted of an exercise performance of all categories during 6 weeks between once and twice a day on average (range 169-336)...high level of exercise performance was defined as an exercise performance of all exercise categories during 6 weeks at least twice a day on average (range 337-504)...To gain insight into which exercises were performed most often, the diaries were analyzed in more detail regarding the frequency of exercising (1-3 times) and type of exercise (four categories). Exercise performance by exercise format was defined as how well the prescribed exercise regimen was followed by patients, following a specific format (online exercising or exercising by leaflet or booklet).  Feasibility - HM is defined to be feasible in case of an uptake percentage >50%, adherence rate >50%, and when >50% of the patients perform at least the minimum number of exercises (168) during 6 weeks (moderate or high performance level). Barriers and Facilitators to Exercise - Reports of the coaching sessions were used to identify patients’ perceived barriers and facilitators to perform HM during (C)RT. | Adherence and exercise performance level: Of the 33 patients who were interested in performing exercises, 21 patients started and kept up exercising for 6 weeks (64% adherence rate). Of the 33 patients, 14 patients (42%) were performing the exercises at a low level (exercise frequency range of 4-167 during 6 weeks), 10 patients (30%) were exercising at a moderate level (exercise frequency range of 196-332), and 9 patients (27%) were exercising at a high level (exercise frequency range of 372-495). Feasibility: HM appears to be feasible in general, with an uptake percentage >50% (in the present study 83%), with an adherence rate >50% (in the present study 64%), and with a moderate to high performance level >50% of the patients performing exercises in all categories at least once a day on average (in the present study, 58% of the participants). |
| Ingrid V. Bassett, et al., 2016  United States | Patient | Primarily intervention was carried over 4 months - but outcomes were assessed to up to 9 months. Primary outcome completion of at least 3 months of ART or 6 months of TB treatment for coinfected (HIV and TB) participants. | Variable | Other: Included face-to-face, calls, and SMS. | We and others have documented high rates of loss to follow-up and mortality after HIV or TB diagnosis but before treatment initiation. Improving linkage to HIV and TB care before ART initiation could lead to substantial clinical and population benefits. Psychosocial factors (eg, stigma, discrimination, preference for traditional medicines), structural actors (eg, poverty, distance to clinic), and clinic system characteristics (eg, rigid policies, long wait times) are among the reasons given for not initiating treatment in sub-Saharan Africa. However, few studies have evaluated interventions to improve initiation of care after diagnosis. | The primary outcome was linkage to and initial retention in care 9 months after enrollment for living participants eligible for ART and/or TB treatment. For HIV-infected participants with negative TB testing at enrollment, the primary outcome was 3 months on ART—documented by initiation date and subsequent ART dispensing dates at study sites. For HIV/TB coinfected participants, linkage and retention also included 6-month TB treatment completion—documented by TB treatment outcome from study site TB registry or TB Control Programme database. Coinfected participants who were ART-eligible were considered to have reached the primary outcome if they reached either the HIV or TB outcome. Coinfected participants not ART-eligible were considered to have reached the primary outcome if they reached the TB outcome.  Mortality at 9 months was a secondary outcome. The primary analysis was intention to treat, with participant outcomes compared according to assigned study arm. Comparisons were performed using x2; relative risks were calculated by log-binomial regression, with participants having at least 3 months on ART or TB treatment completion considered to have successfully reached study outcome. We assumed that participants without available data from site registries related to ART initiation or TB treatment completion did not reach study outcome. We also assessed number of call attempts by the navigator per participant and number of calls that successfully reached the participant, as a measure of fidelity to the intervention. Participants not reached at all were considered as having zero navigator phone contacts, though they received SMS per protocol. | From August 11, 2010 to January 16, 2013, were screened 6536 people. Of those, 4954 (76%) were eligible and 4903 (99%) enrolled (Fig. 1). The most common reasons for ineligibility included: previous HIV diagnosis (988, 62%),18 years old (277, 18%), and unwilling to share HIV/TB test results (208, 13%). A total of 1899 (39%) enrolled subjects were newly diagnosed with HIV at enrollment of whom 967 (51%) were randomized to the intervention and 932 (49%) to usual care. Overall, 49% were female and mean age was 35 years (SD 10). Baseline demographic characteristics were balanced (Table 1). HIV prevalence ranged from 31% to 52% among enrollment sites. A CD4 count was available for 1659 (87%) HIV-infected participants [772/932 (83%) in usual care and 887/967 (92%) in intervention]. Median CD4 was 192/mL (IQR72–346/uL) and similar across arms: usual care participants had median CD4 200/mL (IQR 72–363/uL) and navigator arm participants had median CD4 186/uL (IQR 72–332/uL). One thousand one hundred forty-six (60%) participants were ART-eligible based on South African guidelines at enrollment, with a median CD4 112/mL (IQR 47–203/uL). Of the 1899 HIV-infected participants, 1685 (89%) had available TB culture data. Three hundred sixty-nine (22%) were TB positive by acid-fast bacillus smear and/or culture. An additional 154 participants were diagnosed with TB outside the study by testing performed on the day of enrollment (included as eligible for primary outcome): 83 chest X-ray, 36 acid-fastbacillus smear, 1 biopsy, 15 ultrasound, 1 clinical indication, 9culture, 1 GeneXpert, and 8 unknown. Two hundred ninety-three (30%) of intervention arm participants and 230 (25%) of usual care participants were coinfected with TB. Eighty-two ofthe HIV/TB coinfected participants were not ART-eligible, representing 4% of the HIV-infected. Forty of these were in theintervention arm and 42 in usual care. The proportion of subjects with successfuloutcomes was virtually identical at 0–2 (22%) and 3–4(23%) calls; however, when call frequency reached$5, theproportion reaching study outcome increased to 30% |
| Jacqueline N. Casillas et al., 2019  United States | Other: Peer navigation arm: Peer navigator. Text messaging arm: Not clearly specified. | Variable between intervention arms. Text messaging intervention - 8 weeks | Variable between intervention arms. | Other: Calls and text messages. | Text messaging arm: One appropriate way to reach out to AYAs in order to disseminate health information is through mobile messaging technology. A Pew report found that the use of mobile phones cuts across sex, race/ethnicity, and household income: over 70% of AYAs over 14 years old have their own cell phone. Almost all of these phones have Short Message Service (SMS), or text-messaging, capabilities. [20, 21] Previous studies have shown mobile phone technology utilizing SMS is a cost-effective way to deliver important healthcare information and education. [22] It is also effective in sending health-related reminders and improving treatment plan compliance rates. [23–27] One previous study showed that an SMS-based tool can assist AYA survivors in coordinating late effect screening appointments, facilitating a partnership with their survivorship care team, and connecting them with relevant community resources. [28] Though utilizing mobile technology is seen as an emerging avenue for self-management of disease [29–32] and is a growing area within cancer survivorship research [33, 34], there have not been studies comparing the use of a text-messaging program to the traditional model of internet-based education for childhood cancer survivors.  Peer-navigation arm: Given their success in other high-risk cancer populations, the use of a peer navigation model may be another innovative approach to address AYA-specific barriers and improve their knowledge and intent to seek survivorship care. Currently, there are no studies that have examined the efficacy of peer navigator programs compared to other models to educate AYA cancer survivors.  Institute of Medicine (IOM) recommended that childhood cancer survivors receive life-long, risk-based survivorship care for surveillance, prevention, and treatment of late effects. Yet less than 50% of the childhood cancer survivor population currently receives the recommended survivorship care. In particular, AYA survivors face several healthcare barriers. These include patient-related barriers, such as survivors’ lack of education on their need for longitudinal survivorship care and their risk for late effects. These obstacles are unique when compared to survivors of adult malignancies, as AYA survivors face transition barriers moving into adult-centered healthcare. Therefore, age-appropriate educational interventions to improve the receipt of survivorship care in AYA survivors is warranted. | All participants completed both pre- and post-intervention paper questionnaires to assess the primary outcome variables of (1) knowledge regarding risk of late effects, (2) knowledge, attitudes, and self-efficacy for survivorship care planning, and (3) knowledge, attitudes, and self-efficacy for health insurance planning. These were mailed to survivors and their families both pre- and post-intervention...There was an eight-week period between administration of the pre- and post-intervention questionnaires.  1. Survivorship care knowledge was first assessed by asking participants whether they understood the term “late effects”. Survivorship care knowledge was further assessed using three items that asked participants to rate reasons for receiving survivorship care on a five-point Likert scale. A knowledge scale was formed as the mean of these items.  2. Survivorship care attitude was assessed using four items rated as to their importance in a cancer survivor’s care, which included domains of receipt of survivorship care plan, access to medical care, health promotion, and health insurance coverage. A five-point Likert scale was used. A summary scale averaging these items had Cronbach alpha of 0.75/0.78 at baseline/follow-up (B/F).  3. Survivors’ self-efficacy assessments had three domains: late effects knowledge, survivorship care planning, and health insurance planning. Late effects self-efficacy was assessed using three items; the summary scale averaging these items had Cronbach alpha of 0.87/0.88 at B/F. Survivorship care planning self-efficacy was assessed using three items; the summary scale had Cronbach alpha of 0.93/0.93 at B/F. Self-efficacy for health insurance planning was assessed using five items; the summary scale had Cronbach alpha of 0.94/0.95 at B/F. All self-efficacy items and scales were assessed on a five-point Likert scale. | Table 1 shows demographics, health statuses, and baseline survey items regarding survivorship identity for the 78 study participants (completed the baseline survey and were randomized to groups). The percentage of non-completers did not differ significantly among the three arms (p=0.21; Fisher exact test). The sample was ethnically diverse. Eighteen percent reported having no current health insurance, and survivors who did not complete the follow-up survey were more likely to lack health insurance than survivors who completed the full study (14% {10/71} for completers versus 57% {4/7} for non-completers, p<0.05). Otherwise, there were no statistically significant differences between study completers (n=71) versus non-completers (n=7) nor between the three arms. Most survivors described their current overall and emotional health status as very good or excellent. Survivorship identity responses were largely consistent with a positive survivorship identity. - Table 2 summarizes late effects and survivorship care knowledge outcomes for study completers. Knowledge of the term “late effects” was high at baseline. The text-messaging group had a significant increase in late effects knowledge as well as survivorship care knowledge scale scores from pre- to posttest. The text-messaging group also had a significantly greater increase in the overall survivorship care knowledge scale score and two of three subscale items when compared to the control group. The peer navigation group showed no significant differences in knowledge items compared to the control group. - Table 3 summarizes survivorship care attitude and self-efficacy outcomes for study completers. Both intervention groups showed increases on the survivorship care attitude scale compared to the control group, with medium effect sizes of 0.40 for the peer navigation arm and 0.33 for the text-messaging arm.[50] The peer navigation group also had a significant increase in attitude scale scores from pre- to posttest. The peer navigation group had significant increases with medium to large effect sizes for the late effects, survivorship care planning, and health insurance self-efficacy scales in addition to most subscale items; this group also showed significant increases in late effects and health insurance self-efficacy scale scores from pre- to posttest. The text-messaging group showed no significant differences in self-efficacy items compared to the control group. |
| Jai N Darvall et al., 2020  Canada | Other: Combination of patient navigation and health coaching. | Not specified clearly, but during gestation period. | Variable | Other: App, face-to-face, telephone, etc. | Not specified. | The primary aim of this feasibility trial was to refine and test the trial protocol for a follow-on large, multicenter trial. Specific feasibility outcomes were recruitment feasibility, engagement and recruitment rate, maintenance of blinding of the control group to pedometer step count (concealment of pedometer display with tamperproof tape), participant compliance with wearing pedometers (days with missing pedometer data) and syncing data regularly, participant retention to study conclusion, and data integrity and completeness of uploaded step counts to investigators. Further secondary aims to guide a definitive multicenter trial were to examine efficacy in increasing step count to a target of 10,000 steps daily in pregnant women with obesity via feedback from the pedometer, evaluate the added benefit of investigator feedback compared with participant self-monitoring alone on the reduction in excessive GWG of participants, and assess the magnitude of any effect to further inform sample size calculation for a definitive trial. | Feasibility Outcomes: Recruitment and retention rates were feasible, with all 30 participants recruited within a 10-week period, and a dropout rate of 10% (2 withdrawals and 1 stillbirth). Target population recruitment feasibility was also adequate, with an annual caseload of >1000 pregnant women with obesity seen at Sunshine Hospital. Control group blinding was adequate, with concealment of pedometer display maintained at each check. Patient compliance with wearing pedometers was problematic, with a percentage of days with missing data mean (SD) of 23.4% (20.6%), 39.5% (32.4%), and 21.2% (16.0%) in control, app, and app-coach groups, respectively. Over the study duration, 4 pedometers were lost, requiring replacement. Overall, regular data syncing via automatic mobile phone connection was feasible in app and app-coach group patients, although required troubleshooting in 5 women (1 manually and 4 remotely via telephone).  Activity Data: There was no evidence of a nonlinear association between the baseline and outcome variables. Therefore, the variables were entered into the statistical models without transformation. Results of the linear mixed model investigating activity level are presented in Table 2. The estimated mean baseline daily active minutes for a 30-year-old nulliparous control patient with a BMI of 35 kg/m2 and between 61 and 90 gestational days was 14.5 min. Compared with control patients, there was no difference in active minutes for patients in the app or app-coach groups. There were also no significant differences for any group in activity level trends across the gestational period (Figure 2). A 1-year increase in age was associated with an estimated increase in the daily activity of 0.6 min (95% CI 0.1 to 1.2 min, P=.03, and a 1 kg/m2 increase in BMI was associated with an estimated reduction in the daily activity of 0.9 min (95% CI 0.3 to 1.5 min, P=.005). The estimated effect for parity was each previous live birth being associated with a decrease in the daily activity of 4.5 min (95% CI 0.7 to 8.2 minutes, P=.02).  Step Counts: The estimated mean baseline daily step count for a 30-year-old nulliparous control patient, with a BMI of 35 kg/m2 and between 61 and 90 gestational days was 5455 steps (Table 3). Gestation day was the only variable with a statistically significant effect on step count (decrease of 7.80 steps/day for each additional day of pregnancy; 95% CI 2.91 to 12.69, P=.002), with no difference in daily step counts between groups. From the 12th to the 29th gestational week, daily step counts did not vary between groups. However, a divergence in daily step trajectories was subsequently observed, with the average daily step count decreasing for the app group compared with participants in either the control or app-coach group, although these differences were nonsignificant (Figure 3). Overall, mean daily step counts in all groups remained in the sedentary (5000 steps/day) or low activity (5000-7499 steps/day) categories for the entire study duration. A step count of over 10,000 daily steps was recorded on 62 days over the study duration, 15 days by 4 control patients, 9 days by 3 app group patients, and 38 days by 6 app-coach patients.  Gestational Weight Gain: Mean (SD) GWG was 13.22 (5.91), 7.91 (4.17), and 13.21 (5.73) kg in control, app group, and app-coach group patients, respectively. When allowing for the increased weight at baseline of app group patients, there was no significant difference between groups in GWG, although accounting for the small sample size and resultant significant uncertainty around this estimate, the direction of effect was toward a reduction in weight gained. The results of the multiple linear regression model are shown in Table 4. An increase in weight at recruitment of 1 kg was associated with a further increase in GWG of 0.89 kg (95% CI 0.72 to 1.06 kg, P<.001). |
| James Balmford et al., 2013  UK | Other: Web-based and/or text-messaging interventions for smoking cessation in users | Variable Surveys at baseline, 1 month, and 7 months | Variable | Other: Mixture of online/web-based and/or text-messaging | Modern communication technologies (e.g. Internet and mobile phones) are increasingly being used to provide information and support to smokers wanting to quit. Two major strengths of these approaches are ease of access, and through computing power, they can automate tailoring of advice to the needs of the individual user, doing so at a fraction of the cost of personalized help delivered by a trained counsellor. Automated tailored advice programs delivered on the Internet have evolved from the pioneering work of Prochaskaet al.[1], and text messaging programs delivered by mobile phone from the TXT to Quit program developed in New Zealand[2]. Both forms of intervention are effective [3–6],including when both elements are combined [7].  Cognitive behavioural model | The baseline survey included questions on sociodemographics (age, gender, level of education and employment status) and smoking and smoking cessation related information, quit attempts, including prior use of internet and mobile tech for cessation assistance.  At a follow-up assessment 1-month post-recruitment, participants who reported any quit activity(i.e. no quit attempt, reduction in cigarette consumption or serious thoughts about quitting), were questioned on use of forms of cessation assistance since joining the study.  onQ - Potentially effective use of onQ was defined as receiving at least 4 days of messages, based on preliminary analyses that showed those who stopped them within 3 days were markedly more likely to rate the program as ‘not at all helpful’. This resulted in three categories of use: no interest; tried only(registered, but did not use or discontinued within3 days) and used.  QuitCoach - As a result, we categorized QuitCoach use as: no interest (no evidence of use of either the public or study version); tried only (self-reported use of the public version or completed an assessment on the study version but did not generate the tailored advice) and used (used study version and generated the tailored advice).  Both - For both the combined conditions, criterion use of either was sufficient to define use. | Participants were 3530 smokers or recent quitters recruited from two sources; those seeking smoking cessation information, mostly recruited over the phone, and a cold-contacted group recruited from an Internet panel. More participants (60.1%) initially accepted the intervention they had been offered than used it (42.5%). Uptake of each intervention differed substantially by both recruitment source and modality (phone or web). onQ was a little more popular overall, especially in the information seeker sample. Highest overall intervention uptake occurred in the choice condition. A web-based intervention is most attractive if the offer to use is made by web, whereas a phone-based intervention is more likely to be used if the offer is made over the phone. Providing automated interventions on multiple platforms allows for maximal choice and greatest overall use of some form of help...Smoking cessation outcomes from the trial are not a focus of this article and are reported elsewhere[13]; in brief, small effects were found for both interventions, but there was no evidence of a beneficial effect of offering or using both |
| Jamilia R. Sly et al., 2014  Germany | Patient | Not specified. | Navigation (may be variable) + 1 initial scheduling call + 2 SMS/text reminders | Other: Patient navigation + scheduling call + SMS/text reminders | Mobile health (mHealth) technology, one form of information and communication technology, is changing the way health care access and delivery is approached. Short message service (SMS) or text messages allow users to send and receive text messages on mobile devices (e.g., cellular phones). Each message is up to 160 characters in length. Text messages can be sent quickly and are relatively low cost. Mobile devices are an exception to the digital divide. Due to technological advances, many people are accessing the inter-net and communicating through cellular phones. Recent evidence suggests that the divide is narrowing in this respect, especially when mobile devices are considered. Minorities, particularly African-Americans and Hispanics, are more likely to use SMS features on their mobile phones than their white counterparts [14]. For example, African-Americans are more likely to receive health information via text messages [15]...Furthermore, while mHealth technologies have the potential to improve population health outcomes, the use of SMS still requires a certain level of literacy. Researchers should also consider how the elderly or individuals without advanced technical skills evaluate SMS reminders or participate in the mHealth interventions...The use of automated SMS reminders may be a more cost-effective approach to PN, but it is unknown whether using this technology in conjunction with PN is efficacious in African-American and Hispanic, low-income, older adult populations. | Sociodemographic characteristics (age, gender, race/ethnicity) were measured via the patient’s medical record. The main outcome measure was SC completion, which was retrieved from the patient’s medical record following their scheduled appointment date.  Although 30 participants were recruited, consented, and enrolled, only 24 were scheduled for an appointment and thus included in analysis. Because we were most interested in the clinical application of SMS reminders for SC appointment reminders, we conducted an intent-to-treat analysis of the sample. | Only 46.2 % (n=6) of participants in the non-SMS group completed a SC compared to 72.7 % (n=8) of participants in the SMS group (see Table2). There was not a statistically significant difference in the completion rates between the two groups (p=0.19). Based on these results, power calculations indicated that a larger sample size (N=179) would have resulted in statistical significance.  Overall, of the 14 participants who completed a colonoscopy, 23.1 % (n=3) had fair or poor prep quality and 76.9 %(n=10) had good or excellent prep quality (prep quality rating was missing for one participant). Prep quality between the study arms did not differ statistically (p=.49). Among participants randomized to the non-SMS group, only one participant(20 %) had a poor prep, while four participants had good or excellent prep quality (80 %). In the SMS group, two participants had poor or fair prep quality (25 %), while the other six participants had good prep quality (75 %). |
| Jason Roberge Andrew et al., 2020  United States | Patient | 45 days | Not specified, variable | Online | Not specified | The primary outcome was the conversion of an ED encounter to hospital admission. Secondary outcomes included 45-day follow-up encounters with a self-harm diagnosis and post discharge acute care use. | The percentage of patient encounters with follow-up encounters having a self-harm diagnosis was significantly lower in the intervention group compared with the usual care group |
| Jason Roberge et al., 2020  United States | Patient | 45 days | Variable - Initial video introduction + Patients received a follow-up telephone call by a navigator within 24 to 72 hours from ED discharge + then at least weekly for up to 45 days | Other: Video, telephone, etc. | Not specified. | The primary outcome was conversion from ED to hospital admission. Admission data were obtained through the health care system’s existing medical record abstraction process, which populates a quality improvement registry. The registry enables centralized, multistate bed management for behavioral health inpatient units. The abstraction personnel were unaware of the study. Leveraging this registry allowed for the capture of data on admissions, both within and external to Atrium Health.  Secondary outcomes assessed acute care use consisting of inpatient, observation, and ED encounters to any Atrium Health facility. We examined single-site and multisite 30-day admission rates (both inpatient only and inpatient plus observation) and 45-day use rates (inpatient, observation, and ED). The 45-day rate of return to an ED with a telepsychiatric consultation was also examined. Death records and International Statistical Classification of Diseases and Related Health Problems, Tenth Revision billing codes for self-harm (R45.851, R45.850, T50.902A, X78.8XXA,T14.91, T42.4X2A, X83.8XXA, T43.502D, T43.592A, T43.202A, T65.92XA, T43.502A, X78.8XXD,X78.9XXA, T43.202D, T43.212A, and T43.222D) were collected. The 45-day rate of follow-up encounters (inpatient or ambulatory) with a self-harm diagnosis was assessed. Consistent with the trial’s pragmatic design, and to allow for nondifferential outcomes assessment, all outcomes data were collected and available as part of routine care. | The 45-day all-cause, nonelective health care use (inpatient, observation, and ED) encounters comprised 34.7% (112 of 323) of the intervention group vs 28.3% (89 of 314) of the usual care group(odds ratio, 1.35; 95% CI, 0.96-1.90;P= .08) (Table 2). The 45-day post discharge inpatient and ED admission rates were numerically but not statistically significantly higher in the intervention group(inpatient admission, 5.3% [17 of 323] vs 4.1% [13 of 314];P= .50; ED admission, 29.7% [96 of 323] vs25.2% [79 of 314];P= .20), and the observation admission rates were similar (5.9% [19 of 323] vs6.1% [19 of 314];P= .93). The percentage of patients who had a 45-day post discharge ED encounter with a telepsychiatric consultation was 16.4% (53 of 323) in the intervention group and 15.6% (49 of314) in the usual care group (odds ratio, 1.07; 95% CI, 0.70-1.64;P= .76). Significantly fewer patients in the intervention group had a follow-up encounter involving a self-harm diagnosis within 45 days compared with patients in the usual care group (36.8% [119 of 323] vs 45.5% [143 of 314];P= .03). |
| John C et al., 2020  United States | Peer | 8 weeks | Variable | Online | It is accepted that the best treatment strategy for end-stage renal disease (ESRD) is a LDKT. Though Black patients account for only 12.5% of all LKDTs, White patients account for 65.9% in the United States.4 Since LDKT is a voluntary act on behalf of the donor, this stresses the need to develop culturally-tailored, theory-guided programs to increase LDKTs in Black waitlist candidates. Prior studies have suggested several causes why Black patients do not approach others about living donation. These include access to care, lack of education about LDKT, common misconceptions with potential donors (PDs), fear about sharing personal medical history, poor understanding about surgical procedures and risks, religious concerns and a general lack of skills on how to effectively strategize to advocate for a PD.7–13 When addressing these causes, various programs have shown positive changes in LDKT attitudes, behaviors, and in some cases, improvements in LDKT rates.14–16...To overcome transportation and logistical barriers associated with in-person programs, new strategies are needed. Specifically, technology-delivered interventions to aid patients’ abilities to develop strategies to identify PDs may be plausible. Augmenting education programs with behavioral tactics, such as peer mentorship, may further provide a stronger effect. Peer mentors are people who can personally relate to patients through shared experiences and provide knowledge and guidance. Implementing behavioral strategies through patient navigator and peer-mentorship have shown high acceptability in various in-person settings including solid organ transplantation.17–19 To our knowledge, no program has used both tactics using online education in combination with distance-based peer-mentoring to increase LDKTs among Black kidney transplant eligible patients. | Demographics were taken at baseline and included, age, sex, marital status, education, employment status, months on dialysis, and self reported health status (i.e., Likert scale using 1–5 with higher scores representing better health).  Primary feasibility outcomes included tolerability (i.e., retention rates), fidelity (i.e., video module completion and chat session completion), and attitudinal and knowledge questionnaire outcomes on LDKT knowledge, willingness to speak to others about LDKT, PD concerns, and LDKT self-efficacy using validated questionnaires by Rodrigue et al.16,23 and Waterman et al.24 These included: (1) Knowledge about Living Donation Questionnaire (i.e., 15 T/F statements: correct answers summed [range 0–15]; reliability using Kuder-Richardson 20 formula (KR-20) = .72, our sample’s KR-20 = .54 ),23 (2) Concerns about Living Donation Questionnaire (i.e., 23 T/F statements: two items discarded, with True statements summed [range 0–21 with higher scores depicting more concern]; Cronbach’s alpha = .79, our sample’s Kuder-Richardson 20 = .68),23 (3) Willingness to Discuss Living Donation Questionnaire (i.e., Three questions with each being a single-item 7-point Likert scale choice [Not at all-extremely, range 1—7]: “How willing are you to talk to [1.family, 2. friends, 3. strangers] about donating a kidney to you for transplantation”),23 and (4) Confidence (i.e., self-efficacy) about LDKT (i.e., 13, 5-point Likert scale items [not at all confident-extremely confident; scores averaged, range 1–5 with higher scores depicting higher self-efficacy], our sample’s Cronbach’s alpha = .90).  Secondary (i.e., at 6 months) and exploratory (i.e., at 3 and 12 months) outcome measures included the number of PDs screened through calls to the center, PDs receiving in-person transplant evaluations, and PDs completing LDKTs with study participants. | Primary feasibility outcomes consisted of program tolerability, defined by program retention rates, fidelity, defined by adherence to program elements and attitudinal and LDKT knowledge. Secondary outcomes included the number of transplant center living donor screenings, PDs completing transplant evaluations and LDKTs at 6 and 12 months. Implications of this study will aid in defining if LOVED is a feasible program prior to a large, multi-center trial. |
| Judith Ann Adams et al., 2019  United States | Other: -1 "patient-peer navigator" (employee who was HIV positive with 12 years of experience at the clinic - trained in motivational interviewing and peer support) -Other clinical staff involved in intervention (e.g., registration staff, case manager, physician assistant, administrative director, medical assistant) | Not specified or clear, but interventions (previsit interventions) carried before patients' next health appointment. Except for patients who missed their appointments, they were also given a 24-hour follow up call. | Not specified or clear, but variable. | Hybrid | Not exclusively specified, but study's literature review findings can provide rationale for the interventions:  A systematic analysis of 5 randomized controlled trials determined that the timing of the appointment reminders within 48 hours before the scheduled appointment was a more critical determinant of whether patients attended scheduled appointments than the mode of delivery. Another systematic review of 8 studies concluded that text message reminders were as effective as phone call reminders; however, text messages had the added benefit of being more cost-effective. In addition, text messaging was the preferred mode of appointment reminders for younger patients.  Once a risk factor is identified, efforts to reduce barriers to care may improve appointment attendance. | Risk assessment: The team designed protocols for interventions based on Woodward’s Risk Prediction Tool (RPT). Patients who scored between 2 and 3 on Woodward’s RPT were considered at medium risk for missing their next appointment, and those scoring between 4 and 8 were considered to be at high risk.  The outcome measure for this project was the percentage of no-show appointments. No-show appointments were reviewed daily and reported as monthly rates by calculating the ratio of no-show appointments to the total number of scheduled appointments. The no-show rate during the project period was compared with that in the same 5 months in the previous year.  Process measures for this project included the frequency of text message and phone appointment reminders, the number of completed follow-up calls, PVP episodes, home visits, and same-day appointments made by the patient-peer navigator. | A preliminary review of data in Month 1 of implementation revealed that Woodward’s RPT did not adequately identify patients who missed their appointments as being at risk.....A re-stratification process was conducted to determine additional patients most at risk to miss the next scheduled appointment by targeting patients who missed greater than 3 appointments in the previous 6 months....Re-stratification identified an additional 82 patients for PVP intervention and 1 patient for the home visit intervention. |
| Katherine E. Miller et al., 2019  United States | Other: Coaching | Not specified, variable | Variable | Other: App | For new technologies to be integrated into health care settings, clinicians need to perceive their value and be willing to adopt them as part of a treatment plan. Indeed, per the diffusion of innovation theory, the adoption rate and spread of an innovation (e.g., mobile app technology) rely heavily on the perceptions of the innovation by potential users. | Provider perceptions A 35-item study-specific measure of the five diffusion of innovation theory constructs was created to assess clinicians’ perceptions of CBT-I Coach (see Table 2 for items). Clinicians were also asked whether they had recommended CBT-I Coach to colleagues (endorsement). | Fifty percent of clinicians reported using CBT-I Coach, with 98% intending to continue use. The app was perceived to increase sleep diary completion and homework compliance. Clinicians viewed the app as providing accessibility to helpful tools and improving patient engagement. Of those not using the app, 83% endorsed intention to use it. Reasons for nonuse were lack of patient access to smart phones, not being aware of the app, not having time to learn it, and inability to directly access app data. Those who reported using CBT-I Coach had more favorable perceptions across all constructs (p < .01 – p < .001), except relative advantage, com- pared to nonusers. Users perceived it as less complex and more compatible with their practice than nonusers. Conclusions: Continued efforts are needed to increase adoption and enhance use of CBT-I Coach, as well as study if reported benefits can be evidenced more directly. |
| Kathryn H et al., 2021  United States | Patient | 3 months | Variable | Other: Software program + patient navigator through telephone calls/in-person | Metastatic breast cancer (MBC) survival is improving, yet symptoms associated with the disease process and treatments remain a significant burden [1]. As returning for additional visits may be challenging for MBC patients, supportive, technology-based care interventions that do not require additional on-site appointments could be helpful. | Our primary outcomes were acceptability (i.e., proportion of women who agreed to participate) and feasibility (i.e., proportion of women who consented and participated for at least one month of the program). Our a priori rates were 50% for acceptability and 33% for feasibility. We also evaluated patient satisfaction by asking whether the interventions were perceived by participants as useful. Each day, on the tablet, patients were asked “So that I may learn from your feedback, did you find the service I suggested yesterday to be helpful?”. Possible answers were yes and no. In addition, we evaluated the cost of the intervention by estimating the non-healthcare provider patient navigator time, equipment cost, and cellular data cost. At baseline and 3 months, participants completed self-report assessments of four symptoms. Sleep quality and sleep problems were assessed with the Pittsburgh Sleep Quality Index (PSQI) [3]. Global fatigue was measured with the Brief Fatigue Inventory [4]. Pain severity and pain interference were measured with the Brief Pain Inventory (Short Form) [5]. Distress was assessed using the tool used by the Penn State Cancer Institute to evaluate distress in the clinic. | Sixty-eight percent of patients approached accepted the tablet-based intervention. Patients interacted with the tablet 48%of possible days. Patient satisfaction ranged from 83 for walking to 49% for the psychological interventions. The cost of delivering Nurse AMIE for 3 months was $570.23. Small nonsignificant improvements were found for fatigue (d=0.24).Nonsignificant, but potentially clinically meaningful, moderate reductions were found for sleep (d=0.65) and distress (d=0.74). |
| Kathy J. Helzlsouer et al., 2016  United States | Other: Nurse and social worker navigators | 1 year | Variable, contacted every 2 weeks by navigator | Hybrid | Socioeconomic disparities negatively impact completion of adjuvant breast cancer treat­ment. Navigation programs may improve treatment completion but may not be accessible to all patients, especially in low-resource communities. | Questionnaires, administered at baseline and at 12 months, included information on demographics, planned treatment, Functional Assessment of Cancer Therapy-Breast (FACT-B), health history, Impact of Events Scale-Revised (IES-R), and visual analog scales (VASs) for pain, mood, distress, and fatigue. Program usability was assessed at 3 months. An open-ended comment section was available on each questionnaire. Medical records were abstracted to determine treatment completion, hormone initiation, unscheduled outpatient visits, ED visits, and hospitalizations. | The majority of participants were unemployed or on disability (68%) and were nonwhite (67%). Those randomized to the intervention had lower education levels and were slightly older than those on the comparison arm (P= .04). Two patients on the intervention arm refused part or all recommended treatments and 6 patients on the comparison arm refused some or all recommended treatments (Padj = .08 for number of treatment refusals)  86% of participants on the navigator arm and 76% on the comparison arm completed the 12-month questionnaire; baseline characteristics did not differ between responder and non-responders of the final questionnaire.  At the 12-month follow-up, no statistically significant differences were observed in self-rated pain, distress, fa­tigue, mood, and quality-of-life scores. There were no statistically significant differ­ences between the 2 study groups in missed visits (1 or more missed visits: 6.1% for the intervention and 8.2% for the comparison group [P = .85]). Although a higher proportion of patients on the intervention arm had at least 1 ED visit (39% vs 29%) and hospitalization (43% vs 33%), these differences were not statistically signifi­cant (P = .19 and P =.39, respectively; data not shown).  Patients on the intervention arm were contacted an average of 29 times during the 12-month period. The median contact duration was 30 minutes (range, 2-140 minutes). |
| Kelli D et al., 2021  UK | Patient | 8 weeks | PainTRAINER involves 8 modules; participants were instructed to complete one module weekly, along with practice activities for each cognitive or behavioral coping skill. | Online | Not specified | Feasibility and acceptability measures Feasibility metrics included proportions of eligible and enrolled patients, proportion of completed follow-up assessments, reasons for ineligibility and dropout, and number of participants accessing the painTRAINER program. We also asked for participants’ feedback about the intervention following completion. This feedback included an overall rating of the helpfulness of painTRAINER with respect to managing or coping with SLE symptoms (0 = Not at all helpful to 10 = Very helpful), ratings of the helpfulness of each coping skill addressed (0 = Not at all helpful to 10 = Very helpful), and an open-ended question asking participants for recommendations for improving painTRAINER. We obtained data from the painTRAINER program regarding modules completed by each participant. | PROMIS pain interference instrument (form 6a) This 6-item, validated measure assesses self-reported consequences of pain across aspects of life including social, cognitive, emotional, physical and recreational activities [19]. Items are scored on a Likert scale of 1 (not at all) to 5 (very much), with higher scores indicating greater pain interference.  PROMIS-29 This 29-item scale covers 7 domains of self-reported health: Depression, Anxiety, Physical Function, Pain Interference, Fatigue, Sleep Disturbance, and Ability to Participate in Social Roles and Activities [21]. All items are scored using a 5-point Likert format, with higher scores indicating more of that domain; therefore, higher scores indicate a more positive outcome for Physical Function and Ability to Participate and a more negative outcome for the other domains.  Coping strategies questionnaire (secondary outcome) This 48-item scale covers 7 domains: Catastrophizing, Diverting Attention, Ignoring Sensations, Coping Self-Statements, Reinterpreting Pain Sensations, Praying-Hoping, Increasing Behavioral Activities [22, 23]. Consistent with other studies [24, 25], we calculated a Coping Attempts Score, which summed all domains other than Catastrophizing.  LupusPRO (secondary outcome) LupusPRO (v1.8) is a 43-item reliable, validated self-report measure including domains of Lupus Symptoms, Lupus Medication, Physical Health, Emotional Health, Pain, Sleep, Procreation, Cognition, Body Image, Desires-Goals, Coping, Social Support and Satisfaction with Care [26]. We assessed separate domains, as well as health-related quality of life and non-health-related quality of life subscales that combined domains.  Demographic and clinical characteristics We assessed self-reported age, sex, race / ethnicity, household financial status (living comfortably or just meeting basic expenses with a little left over for extras vs. just meeting basic expenses or don’t have enough to meet basic expenses), education level (no college vs at least some college education), marital status (married or living with a partner as married vs. other), work status (working part or full time vs. other), children (and their ages), body mass index, duration of SLE symptoms and time since diagnosis, self-rated health, and comorbid illnesses. |
| Kerstin Denecke et al., 2018  United States | Patient | Not specified, variable | Not specified, variable | Other: App | Not specified | Not specified | Not specified |
| Lara Weinstein et al., 2019  United States | Patient | 6 months | Not specified clearly. | Other: Online software application (used in person with patient and navigator - decision counselor - present) + in-person navigation | Theoretical Model: To our knowledge, there have been no reports in the literature on the use of theory-based intervention methods and SDM to increase mammography screening in women with SMI. The PHM, a well-accepted self-regulatory framework forms the basis for the intervention in this study, as shown in Figure 1. The PHM considers the “self-system” as critical in explaining the use of preventive health modalities, such as screening, that are intended to diagnose, risk-stratify, and prevent chronic disease. According to the model, the self-system includes both sociocultural health background; as well as cognitive, affective, and social representations about disease, risk, and available preventive health behavior alternatives.17-19 The PHM, which has been useful in predicting outcomes such as cancer screening intention and utilization,20,21 has also been used to develop the decision counseling intervention used in this study. | We administered in-person surveys at baseline, 1 month post baseline, and 6 months post baseline. To potentially identify any relationship between the participants’ mental health and study participation and experience, we included measures of psychiatric symptoms and mental health recovery.24-26 We included a survey based on the PHM, which is used to predict outcomes in cancer screening intention and utilization.27,28 Two additional surveys were included to identify changes in breast cancer knowledge and decision conflict.29,30 Further details of the measurement instruments are listed in Table 1.  The semistructured interview after session 1 was designed to explore the participants’ experience of the experiences and attitudes toward the education and decision support session, with specific questions exploring certain constructs of the PHM, including the relationship between the educational module and the intrapersonal support system and the relationship between the decision support session and the inter-personal decision-making support system. The purpose of the 6-month semistructured interview was to elicit the participants’ experiences and attitudes toward the navigation experience. Specific questions considered additional con-structs of the PHM, including any changes in the intrapersonal support system and the interpersonal decision-making support system. Additional interview questions explored the PHM construct of empowerment and values clarification as related to participants’ experience of control over their health. | Baseline (N = 10) interviews revealed the participants were generally enthusiastic about the education and decision counseling element of the intervention. In terms of the interpersonal self-system, participants appreciated learning about anticipated outcomes. For example, one participant felt it was helpful to learn about the process for having a mammogram...Participants commented on both helpful and unhelpful elements of the education module in terms of overcoming cognitive and informational barriers. For example, one participant commented on the usefulness of the visual information...However, participants found the varying age recommendation confusing...Participants also suggested the educational module include more information on anticipated outcome, for example what to do if the mammogram is abnormal....In the 6-month follow-up (N = 11) interviews, multiple participants commented that the decision-counseling pro-gram “made me think,” and spurred some participants from intention to action...However, some women found they did not need that level of support. |
| Lena Sanci Sylvia et al., 2019  Canada | Other: Combination of peer, patient navigation, and coaching - self-directed service using Link. | 3 months | Variable - up to the participant. | Online | In response to the deficiencies identified in the literature, we developed Link, a website designed to assist young people to find accessible Web-based and computer-based mental health services appropriate for their mental health needs. Link was developed in accordance with the Medical Research Council guidelines for complex interventions [19,20]. We undertook a comparative review of relevant behavior change and help-seeking theories and selected the Theory of Planned Behavior on which to base our program logic and ultimately the functional elements of the technology design; a description of this process has been previously published [21]. In the development phase, participatory design [22] with young people was used to understand the features important to include in Link that would facilitate youth engagement [23]. The program logic of Link thus proposes that by improving attitudes, beliefs, and perceived control of help-seeking, and reducing barriers toward help-seeking, positive affect (PA), and intentions to seek help will increase, which in turn will increase actual help-seeking behaviors [24]. | All outcome measures were collected from both arms at baseline, 1 month, and 3 months postintervention. PA and NA and satisfaction were also measured 2 weeks (immediately) after randomization in both arms to capture effects after first using their respective allocated intervention.  Primary measures: Positive Affect (PA) was measured using the PA scale of the positive and negative affect scale (PANAS) [27]. A PA score was calculated by adding the 10 PA items. The PA score can range between 10 and 50, with higher scores representing higher level of PA. The 10-item PA scale has high internal consistency, is valid and reliable over a 2-month period, and is sensitive to mood fluctuations if used with short-term instructions (eg, now) or to stable traits if used with longer-term instructions (eg, past year)  Secondary measures: Secondary outcomes included PA at all other follow-up points, and the other measures described below.  Negative Affect: NA was measured using the 10-item NA scale of the PANAS [27]. NA reflects an individual’s degree of subjective distress arising from mood states, such as anger, guilt, fear, and nervousness. Low NA is characterized by a state of calmness and serenity. NA is related to self-reported stress, poor coping, and frequency of negative events. Developed alongside the PA scale, the NA scale is also highly internally consistent, largely uncorrelated, and stable at appropriate levels over a 2-month time period (Cronbach alpha reliabilities for intercorrelations and internal consistency reliabilities range from .86 to .90 for PA and from .84 to .87 for NA, with reliabilities unaffected by the time instructions used) [27]. The NA scale was used to indicate if there was an immediate benefit of using Link and if any harms arose from either arm. The 10 NA items were added to calculate a total NA score ranging between 10 and 50, with lower scores representing lower levels of NA.  Psychological Distress: Psychological distress was measured using the Kessler psychological distress scale (K10) [31]. The K10 has good precision in the 90th to 99th percentile range of the population distribution (standard errors of standardized scores in the range 0.20 to 0.25) and maintains consistent psychometric properties across major sociodemographic subsamples [31]. The K10 strongly discriminates between community cases and noncases of structured clinical interview for diagnostic and statistical manual of mental disorders (IV) [31]. The K10 comprises 10 questions asking about the frequency of depressive and anxiety symptoms in the past 4 weeks. Each item is rated on a 5-point scale (1=none of the time and 5=all of the time) and scores are summed to a possible range of 10 to 50, with higher scores indicating higher distress. For the random allocation, participants with a K10 score less than 20 at baseline were classified as likely to be well, whereas participants scoring 20 or more were classified as likely to have a mental disorder. The K10 is a reliable measure with all items of relevance to young people [32]. | There was evidence to support a reduction in the mean NA for the intervention arm compared with the control arm at the immediate and 1-month follow-up time points (Table 3). However, the intervention effect diminished at 3-month follow-up. Sensitivity analyses for PA and NA scores showed that the findings are unlikely to change when the departures from missing at random assumption are assumed to occur in the same way in both study arms. Study conclusions could change if departures from the missing at random assumption differed in the 2 study arms, but we considered this an unlikely scenario as the participants with missing data were similar in the 2 study arms (see Multimedia Appendix 3 for details).  Mean scores on the K10 and the BASH remained relatively stable for the duration of the study for both arms and there was no evidence to support between-arm differences at any time point. There was, however, weak evidence to support that there was greater intention to seek general help at 3-month in the intervention arm compared with the control arm (Difference −0.22, 95% CI −0.44 to −0.009; Table 4). There was no difference between Link and the young people in the control group in how likely they were to not seek help from anyone; however, at 1 and 3 months, they were, on average, less likely to not seek help from anybody compared with the baseline responses.  Link Use and Satisfaction: Of the 205 people randomized to the intervention arm, 160 (160/205, 78%) visited the Link website and 159 (159/160, 99%) moved beyond the first page. At all 3 follow-up time points, a greater proportion of intervention participants reported the information they found with their respective search strategies helpful and felt surer of themselves compared with the control arm (Table 5). At 1 and 3 months, a greater proportion of participants in the intervention arm reported they had found treatment for their problems compared with the control arm participants. Young people in the intervention arm at the immediate and 3-month time points were more likely to feel that they had been guided to an appropriate service, although this was not evident at 1-month.  Help-Seeking Strategy After Randomization: Help-seeking results were difficult to interpret because of missing responses to these questions (percentage with missing responses: 15% at immediate time point, 34% at 1-month, and 33% at 3-month follow-up; Multimedia Appendix 5). Of those who responded, the proportion of young people who reported they did not need help across time points was less than 3%. The majority of the participants reported using at least 1 search strategy, with a greater percentage in the intervention arm compared with the control arm at each follow-up time (Multimedia Appendix 5; Table 1). Of those who did seek help, at the immediate time point (up to 2 weeks postrandomization), more young people in the intervention arm used one or more websites or Web-based services to seek help, compared with the control arm (33.5% vs 15.1%), and fewer of the intervention arm used formal (19.8% vs 35.5%) or informal (18.0% vs 27.1%) sources of support (Multimedia Appendix 5; Table 2). Numbers of young people seeking help via phone lines were small in both the arms across all time points. Help-seeking appeared less frequent at both 1- and 3-month follow-up points than immediately after randomization for young people in both the study arms, with young people from the intervention arm more likely to use Web-based sources of help (website/Web-based service and/or other Web-based method) and young people from the control arm more likely to seek help from formal and informal sources of support. |
| Lorna Cook et al., 2019  Canada | Other: CBT | 1-hour modules, each in turn split into 3 or 4 sessions consisting of a single Web page, with 1 to 2 weeks recommended per module for practice of the techniques. | 1-hour modules, each in turn split into 3 or 4 sessions consisting of a single Web page, with 1 to 2 weeks recommended per module for practice of the techniques. | Online | Not specified - Prevention of depression is a priority to reduce its global disease burden. Targeting specific risk factors, such as rumination, may improve prevention. Rumination-focused Cognitive Behavioral Therapy (RFCBT) was developed to specifically target depressive rumination. | The primary objective of this study was to test whether guided Web-based RFCBT (i-RFCBT) would prevent the incidence of major depression relative to usual care in UK university students. The secondary objective was to test the feasibility and estimated effect sizes of unguided i-RFCBT.  The primary outcome was the onset of a major depressive episode over 15 months, assessed with structured diagnostic interviews at 3 (postintervention), 6, and 15 months post randomization, conducted by telephone, blind to the condition. Secondary outcomes of symptoms of depression and anxiety and levels of worry and rumination were self-assessed through questionnaires at baseline and the same follow-up intervals. | Significant improvements in rumination, worry, and depressive symptoms were found in the short-to-medium term. |
| Lübomira Spassova et al., 2016  UK | Other: Patient navigation through a virtual lifestyle coach (CAPSYS phone line) | Six month trial period | Participants were advised to regularly call the phone-based prevention system CAPSYS (preferably twice a week). | Other: Automated phone line with a virtual lifestyle coach | Early findings in behavioural research support the assumption that the mere task of self-monitoring increases habit awareness, induces reflection on habits, and thus can provoke a positive change of the monitored behaviour [4]. Furthermore, experience has shown that sustained contact is necessary in order to support people in establishing and maintaininglifestyle changes [5]. | The impact of the intervention was assessed based on the changes of cerebro-cardiovascular risk parameters during the six-month trial period, whereby changes in the IC group were compared to those in the control group(SC). Changes in systolic blood pressure, HDL, LDL, HbA1c, glycaemia, triglycerides and BMI were considered as primary dependent measures.  Changes in self-reported weekly consumption of fruits and vegetables, whole grain food and sweets as well as changes in self-reported weekly duration of physical activity and quality of life were analysed as secondary measures.  Due to the low number of smokers among the study participants (SC: 2, IC: 6), smoking habits were not evaluated.  Statistical analyses were carried out on an intention-to-treat basis using RStudio (Version 0.98.978) with a significance level of 0.05 | Detected a statistically significant increase in self-reported fruit and vegetable consumption (pseudo-median of the differences=5.4 servings/week; p=0.04; 95 % CI=[0.5, 10.5]) and a decrease in sweets consumption (pseudo-median of the differences=–2 servings/week; p=0.04; 95 % CI=[–4, –0.00001]) in the intervention group. |
| Lynn F. Reinke et al., 2011  United States | Patient | 60-minute long webinar | Not specified. | Online | Not specified. | Participants were asked to complete a pre webinar Web survey that consisted of 7 questions to assess their knowledge and attitudes regarding end-of-life issues. The survey used multiple-choice questions and Likert scales to measure the participants' current state of end-of-life planning. A post webinar Web survey that consisted of 23 questions was administered immediately after the webinar. The questions were designed to assess knowledge and attitudes about end-of-life issues discussed during the webinar and obtain feedback on the use of Web-conferencing technology. Question formats included short answer, multiple-choice, and Likert scales. Both surveys were developed by the investigators (HQN, RGG, LFR) and reviewed by a content expert (DDC) to assess for face validity. All participants were contacted by phone 3 months after the Webinar to assess whether any changes had occurred in their end-of-life care planning. We asked the participants whether any changes had been made on advanced care planning forms, whether they had shared copies with anyone or discussed their end-of life wishes with anyone, or experienced any adverse emotional responses as a result of the Webinar. | Demographic info: Of the 29 participants who were invited to participate in the study, 7 (24%) agreed to participate, 11 (38%) declined, and no response was received from 11 (38%). As seen in Table 1, the age of participants ranged from 64 to 72, 3 were female. All study participants were Caucasian, with the exception of 1 African American. More than half (57%) were educated at the college level or beyond. The average forced expiratory volume in 1 second(FEV1) percentage predicted was 33%, indicating severe to very severe pulmonary disease. Four participants were on supplemental oxygen therapy. All participants reported being comfortable with Internet browsing and had used the Internet for at least 1 year.  Prewebinar results: The majority of study participants (n = 6) reported having a living will and 5 reported having an advance directive. Fewer used the Medical Durable Power of Attorney (MDPA; n= 4) and Physicians Orders for Life-Sustaining Treatment (POLST; n = 3) forms. One of the participants was not familiar with either of these 2 documents. Of the participants with completed forms, 5 had shared them with their family and significant friends and 4 had shared them with a designated spokesperson. Only 3 participants had shared the forms with their clinicians (Table 2).  End-of-Life Discussions: All participants reported having had end-of-life discussions with their family/significant friends and 6 participants had these discussions with their clinicians. The majority of participants (n = 6) said they were very confident discussing end-of-life issues with family/significant friends and their clinicians and felt their end-of-life treatment would be consistent with their wishes.  Other Information Related to End-of-Life Care: Four participants reported having all the information they needed about end-of-life documents to effectively discuss their end-of-life preferences. Other types of information participants expressed the desire to know more about to facilitate end-of-life care discussions included what dying may be like; end-of-life treatment options such as medications, intubation, oxygen, tube feeding, IV fluids; end-of-life care options such as palliative care, hospice, hospital care; and information about end-of-life financial issues such as insurance and out-of-pocket expenses.  Postwebinar Results—Immediate: Few changes were noted between the pre- and post-webinar survey results (Table 2).Confidence about discussing end-of-life issues with designated spokespersons increased for2 participants. Two respondents noted having more fear and anxiety regarding end-of-life issues after the webinar. These 2 participants did not offer additional information on the rationale for their increase in fear and anxiety nor did they report adverse outcomes. A majority of patients (n = 4) reported that they would have liked to have more discussion in 3areas: symptoms they may experience at the end of life, treatment options, and care options.  Webinar/Technology Feedback: Overall, 5 participants felt the technology was very easy to use. All participants felt it was easy to log onto the webinar and felt it was moderate to very easy to get their microphone working on the day of the webinar. Despite the extensive training and technical troubleshooting prior to the webinar, audio feedback was the most problematic technical issue, especially for participants who used a laptop computer. All participants felt that having a live video of the discussion facilitator and the ability of see other participants would have made it easier to them to follow the discussion. Six participants felt the webinar format was a very acceptable alternative if they were unable to participate in person.  Postwebinar Results—3-Month Follow-Up: Participants reported making no changes on advanced care planning documents related to their end-of-life wishes. However, all participants had taken some form of action since the webinar, including communicating with either their clinician or family members about end-of-life wishes, sharing copies of the documents, learning more about the death and dying process, and symptom management options. None of the participants reported any adverse outcomes as a result of the end-of-life content or discussions. |
| Margarita Elkjaer et al., 2010  UK | Patient | 12 months | Variable - depends on patient: After ET the web-patients were asked to log on to http://www.constant-care.dk and follow the web-program’s recommendation. In case of relapse, patients were requested to log on daily and complete the disease activity score (SCCAI)18untilthey entered the green zone. Patients should then log on once a week for a total of 4 weeks after the initiation of relapse. The short form of disease specific QoL (s-IBDQ)19should be filled in at the beginning and the end of each relapse. Once remission was achieved patients had to use the program once a month until the next relapse occurred. | Online | The natural history of ulcerative colitis is characterised by relapses and remissions.1The majority of patients with ulcerative colitis (91%) have a mild-to-moderate disease course eligible for 5-amino-salicylate acid (5-ASA) treatment.2To prevent relapses, colectomy and colonic cancer there is a need for continuous optimisation of medical treatment via frequent visits in the outpatient clinic.13Lack of easy access to specialised IBD clinics, patients’ education, and lack of under-standing of the importance of early relapse treatment leads to poor compliance and self-adherence.34These limitations were attempted to be overcome by personalised self-management training of patients with ulcerative colitis, which successfully improved disease self-management in 96% of patients without increasing morbidity.5Furthermore, disease monitoring by home automated telemanagement seems promising.6 | At each visit, patients were asked to fill in the following: SCCAI - The SCCAI is a validated symptom-based index (range 0e19)based on clinical symptoms. A score of>5 is used to define a relapse.  s-IBDQ - Short-IBDQ - 10 questions derived from the 32questions IBDQ concerning QoL. It covers four items: bowel symptoms, systemic symptoms, emotional, and social functions. The total score ranges from 10 (worst health) to 70 (best health).  Crohn Colitis Knowledge Score (CCKNOW) - 0 multiple choice questionnaire divided into four items: general IBD understanding (16 questions),medication (six questions), diet (two questions), and complications of IBD (six questions), with a scoring system of one point for each correct answer.  Health Survey SF-36/SF-12 - a generic multi-purpose health survey with 36questions. It yields an eight-scale profile. Scoring on a scale of0 to 100 with mean health of 50. In Dublin SF-12 was used.  Hospital Anxiety and Depression Scale (HADS) - The HADS incudes 14 questions: seven on anxiety (HADS-A),and seven on depression (HADS-D). The scoring scale is divided into three groups: 0-7 normal, 8-10 borderline, >/=11 anxious and/or depressed.  In case of a relapse, patients had to do a Faecal calprotectin (FC) - Results for faecal calprotectin (FC) were blinded for both investigators and patients until the end of the trial where the FC results were compared with the SCCAI to identify eventually over treatment. A cut-off level of#50 mg/kg considered normal.  At the 12 months visits, patients filled the Satisfaction Questionnaire (SQ).  The data were analysed as intention to treat...A p value of < 0.05 was considered statistically significant. | 88% of the web patients preferred using the new approach. Adherence to 4 weeks of acute treatment was increased by 31% in Denmark and 44% in Ireland compared to the control groups. In Denmark IBD knowledge and QoL were significantly improved in web patients. Median relapse duration was 18 days (95% CI10 to 21) in the web versus 77 days (95% CI 46 to 108)in the control group. The number of acute and routine visits to the outpatient clinic was lower in the web than in the control group, resulting in a saving of 189 euro/patient/year. No difference in the relapse frequency, hospitalisation, surgery or adverse events was observed. The historical control group was comparable with the control group. |
| Maricianah Atieno Onono et al., 2019  UK | Not specified | Not specified, | Not specified, variable | Other: SMS, über-like transport system (mobile phone) | The ‘three delays’ model developed by Thaddeus and Maine9 provides a suitable framework for understanding the causes of maternal deaths. These are1 delay in recognising danger signs/decision to seek care,2 delays in reaching a medical facility and3 delay in receiving appropriate care once a facility is reached. We adopted the ‘three delays’ model as a framework to identify the barriers to obstetric care and assess whether a mobile phone-enhanced community-based maternal newborn health intervention that addressed delays 1 and 2 can help women to overcome these barriers. | The main outcome variables were cost of transport to place of delivery (measured in Kenyan Shillings), time taken to reach facility for delivery (measured in minutes and hours from the time the woman or birth partner makes a call for transport), number of ANC visits, number of PNC visits, pregnancy outcomes (defined as either miscarriage, stillbirth and live) and status of mother after birth (alive, dead and unknown). Potential confounders considered included age and education level of women. | Cases (women who received the intervention) had five times higher odds of having four or more ANC visits (aOR=4.7, 95% CI 3.20 to 7.09), three times higher odds of taking between 30 and 60 min to reach a health facility for delivery (aOR=3.14, 95% CI 2.37 to 4.15) and four times higher odds of undergoing at least four PNC visits (aOR=4.10, 95% CI 3.11 to 5.36). |
| Marie Ferrua et al., 2021  Germany | Patient | Data were collected from March 23 to June 5, 2020. | Variable | Hybrid | Telehealth can help maintain continuity of care while limiting the exposure of patients and healthcare workers to the disease. For patients with COVID-19, digital solutions can provide remote patient monitoring to report a worsening clinical status and to offer global support to patients [7]. For an effective implementation of a telehealth solution, it is important to acknowledge the use of internet technologies in con-junction with human practices in a specific context [8]. Moreover, the data feedback loop is a critical component to ensure that telehealth interventions have a high impact on patient care [9]. Nurses are usually at the frontline for monitoring and providing feedback related to the remotely captured data from patients. During the COVID-19 pandemic, nurse navigators (NNs) played a major role in providing optimum healthcare to cancer patients [10–12]. | Tracking indicators: Indicators were defined to follow the evolution of symptoms in patients with COVID-19. The primary indicator was the proportion of patients who were admitted to the hospital. Other indicators included the number of emergency visits, hospitalizations in the intensive care unit, and deaths related to COVID-19.  Secondary outcome - Patient experience: The experience of patients was measured by a survey administered via secured e-mail or by post for those without a valid email address in June 2020. The survey included 25 items on 4 themes: expectations of CAPRI-COVID, benefits of CAPRI-COVID, behavior of NNs, and the CAPRI App. | All 130 (could be an error, actually 129) patients (median age: 59 years; 59.2% female) were monitored during the study period. There were no deaths or admissions to the intensive care unit attributable to COVID-19; 7.8% of patients were hospitalized (excluding scheduled hospitalization), and 17.1% were admitted to the emergency department at least once during the monitoring period. NNs carried out 1412 regular monitoring calls (average of 10.9 calls per patient), while 55% of the patients downloaded the CAPRI App. |
| Martin Angelo et al., 2021  United States | Patient | Not specified. | Not specified. | Other: Telephone calls, SMS, educational videos, website, mail, etc. | Not specified. | Demographic data and FIT screening rates were obtained using an electronic chart review and compiled into a spreadsheet. For the first aim, a retrospective chart review was conducted to identify the overall CRC screening rate for Hispanic adults aged 50–75 years from January 2017 through December 2018 at WCMFC. A simple random sample method was used to obtain the CRC screening rates of 36 participants who met inclusion criteria. The aggregate data were considered as the comparator to the planned project. To measure project outcomes, a query of the electronic health record was conducted by the first author at the end of each month to determine which participants successfully completed the FIT during project implementation. For the second aim, a post-FIT screening questionnaire was used to measure the influence of the intervention on participants’ decisions to complete screening. | Findings showed a 35% increase in the clinic’s FIT rates. Findings also showed that having a PN’s support influenced the decision to complete FIT as compared to the use of secure SMS. |
| Martina Nitsch et al., 2016  Canada | Other: Mobile self-help | 8 weeks | 40 sessions lasting approximately 10 minutes eac | Online | Not specified | Engagement and corresponding usability issues | The analysis of the qualitative data revealed five central themes: layout, navigation, content, support, and engagement conditions. The first three themes highlight usability aspects of the program, while the latter two highlight engagement issues. An easy-to-use format, clear wording, the nature of guidance, and opportunity for interactivity were important issues related to usability. The coach support, time investment, and severity of users’ symptoms, the program’s features and effectiveness, trust, anonymity, and affordability were relevant to engagement. |
| Marybeth Allen et al., 2008  United States | Other: Patient navigation through nurse electronic coaches (e-coaches) - 2 experienced clinic nurses conducted the intervention, jointly monitoring the e-coach inbox on the Internet portal. | The e-coach intervention was designed to occur within 4 weeks, precluding the development of an ongoing relationship between the nurse e-coach and the patient and limiting the amount of coaching that could be conducted. | Variable - Apart from the initial email contact, links to personalized worksheets and resources, and final contact, the frequency of contact is dependent on the ongoing relationship between the nurse e-coaches and each participant/patient, especially for the portal inbox/messaging. | Online | Bandura’s Social Cognitive Theory | Not specified, but unofficially - number of website visits, frequency of contact with e-coaches, frequency of access to resources, etc. | An earlier study of PatientSite users showed that, in general, they are younger (though 7% are at least age 65 years or older) and healthier than nonusers and are more likely to be White (Weingart, Rind, Tofias, & Sands, 2006). The 121 intervention participants ranged in age from 22 to 82 years, with 60% aged 50 years and older. More than half were women (59%), most were White (91%), and 69% had attended 4 or more years of college.  Most popular intervention components - automated and prepared online elements/resources of the patient portal - 50% of participants viewed the website before their health visit. Among those who viewed the Website, 42 individuals (71% of Web site users) opened the worksheets 107 times in the days prior to their doctor visits. Of the 121 participants, 42 (35%) sent 62 e-mails to the e-coach prior to their primary care appointments. Most patients who sent an e-mail to the e-coach were interested in further coaching in preparation for their primary care physician visit (88%). |
| Meelim Kim et al., 2020  Canada | Other: psychologist coaching program | 8 weeks, follow up at 24-weeks | Variable | Online | Developing effective, widely useful, weight management programs is a priority in health care because obesity is a major health problem. | The primary outcome was change in body weight. Other measures, such as change in BMI and body fat mass, were secondary outcomes.  The main outcomes of this study were measured objectively at baseline, 8 weeks, and 24 weeks and included weight (kg) as well as other body compositions. Differences between groups were evaluated using independent t tests and a per-protocol framework. | Mean weight loss at 8 weeks in the digital CBT group was significantly higher than in the control group (– 3.1%, SD 4.5, vs –0.7%, SD 3.4, P=.04). Additionally, the proportion of subjects who attained conventional 5% weight loss from baseline in the digital CBT group was significantly higher than in the control group at 8 weeks (32% [12/38] vs 4% [1/21], P=.02) but not at 24 weeks. |
| Meg Simione Laura et al., 2021  UK | Patient | From the first to third trimester for women participating in the First 1000Days Program. | Variable | Other: In-person, educational materials, text-messaging, videos, health coaching, etc. | Obesity remains highly prevalent and is a major contributor to chronic disease and other adverse con-sequences [1, 2]. Socioeconomic and racial/ ethnic disparities continue to persist despite national prevention efforts [3, 4]. Some of the origins of maternal and childhood obesity are linked to the first 1000days, a period from conception through the first 2 years of life and have life course impacts [5–7]. During pregnancy, behaviors, such as maternal diet and physical activity, maternal anxiety, and connection to resources affect excessive weight gain and postpartum weight retention [8, 9]. As highlighted by the World Health Organization [10], this period represents a critical time for health-promotion interventions to prevent maternal and child-hood obesity. Intervention efforts focused on the first 1000days have shown improvement in behaviors, psychosocial outcomes, and utilization of the Special Supplemental Nutrition Program for Women, Infants and Children (WIC) resulting in improved outcomes for women and their children [11]. These studies have predominately targeted individual-level of change [12–14], while few interventions have focused on a broader context of change using a systems-level approach. | We collected information through self‑administered questionnaires during the first and third trimester of gestation and from electronic health records relating to obesity risk factors. Measures collected included behavior (i.e., diet, physical activity and screen time) and psychosocial (i.e., anxiety) outcomes, as well as enrollment in Women, Infant, and Children (WIC) program. We examined the extent to which participation in the program was associated with changes in behaviors and psychosocial outcomes among women during pregnancy. | Of the 286 women who completed a survey at their initial and third trimester prenatal visit, 264 were included in final analyses. Baseline demographic characteristics of participants are summarized in Table 1. Women were a mean (SD) age of 30.8 (5.51) years and initiated their first prenatal care visit at a mean (SD) gestational age of 10.4 (4.65) weeks. Women had a mean (SD) pre-pregnancy BMI of 27.7 (6.43) kg/m2 and 64% started pregnancy with a BMI≥25kg/m2. At the first trimester visit, 33% of women were enrolled in WIC. 220 (83%) women received patient navigation and/or health coach phone-calls and booklets, 41(16%) received booklets only, and 3 (1%) did not receive a phone-call or booklet. |
| Michelle J. Naughton et al., 2021  Netherlands | Other: Symptom monitoring | 12 months | One monthly survey | Other: SMS messaging or email | Cancer patients often experience symptoms related to their treatment regimens and/or the disease itself, as well as psychosocial concerns. Past research indicates that health care providers systematically underestimate their patients’ moderate or severe symptoms compared to what patients report themselves. Under-estimation, which tends to be more common than the over-estimation of patients’ symptoms, leads to poorer health outcomes and the under-treatment of patients. | Patient reported symptom outcome measures  Patient Health Questionnaire (PHQ-9), and 4 single items assessing fatigue, sleep quality, pain, and global quality of life during the past 7 days rated on a 0 (low) –10 (high) scale | Patients utilizing this voluntary program had an overall mean age of 60.5 (range 26–87), and 85% were non-Hispanic white. iPhones were provided to 42 patients, and navigation services were used by 69 patients. Average adherence with monthly surveys ranged between 75–77%, with breast patients having lower adherence after 5 months. The most commonly reported symptoms across cancer types were moderate levels (scores of 4–7) of fatigue and sleep disturbance. At 6 months, 71–77% of all patients believed the surveys were useful to them and their health care team. |
| Min-Kyung Lee et al., 2021  Canada | Other: Mobile coaching | Not specified | Not specified - variable | Other: Mobile phone | Not specified | The primary outcome was changes in HbA1c over the 12-month study period. The secondary outcomes were diabetes-related health outcomes and diabetes self-management. | There was a significant between-group difference in glycated hemoglobin test (HbA1c) levels for the 12-months study period (P=.011). The HbA1c decrement at 12 months in the UUS:5-8 group was greater than that of the UUS:0-4 group [–0.92 (SD 1.24%) vs –0.33 (SD 0.80%); P=.049]. After adjusting for confounding factors, UUS was significantly associated with changes in HbA1c at 3, 6, and 12 months; the regression coefficients were –0.113 (SD 0.040; P=.006), –0.143 (SD 0.045; P=.002), and – 0.136 (SD 0.052; P=.011), respectively. |
| Monika Jurkeviciute et al., 2020  Canada | Patient | 6 months | Variable | Online | Not specified | Value in the assessments of eHealth | The value of an eHealth intervention applied to similar types of populations but differed in different contexts. In Sweden, patients improved cognitive performance (MMSE mean 0.85, SD 1.62, P<.001), reduced anxiety (EQ-5D-5L mean 0.16, SD 0.54, P=.046), perceived their health better (EQ-5D-5L VAS scale mean 2.6, SD 9.7, P=.035), and both patients and health care professionals were satisfied with the care. However, the Swedish service model demonstrated an increased cost, higher workload for health care professionals, and the intervention was not cost-efficient. In Italy, the patients were satisfied with the care received, and the health care professionals felt empowered and had an acceptable workload. Moreover, the intervention was cost-effective. However, clinical efficacy and quality of life improvements have not been observed. |
| Nadim Mahmud et al., 2019  United States | Patient | Week prior to the procedure | Nine instructional and reminder messages in one week | Other: Text messaging | Because patients have already demonstrated intention to screen by scheduling the procedure, the theoretical focus of intervention is to help move from intention to behavior. | Outcomes included colonoscopy appointment adherence, bowel preparation quality, and colonoscopy completion. | The arms had similar demographics and comorbidities. Intervention patients had higher colonoscopy appointment adherence (90% vs. 62%, p = 0.049). There were no significant differences in preparation quality or procedure completeness. Poststudy surveys indicated high patient satisfaction and perceived usefulness of the program. |
| Natalie Liling et al., 2021  UK | Patient | Not specified (but during COVID isolation stay in hospital) | Variable | Online | During an infectious disease outbreak, contact isolation is a key strategy in mitigating further disease spread. However, patients are often mentally unprepared for the disruptive effects of being placed in an isolation ward. Stress and negative psychological effects are increased in patients affected by a novel infectious disease, as exemplified by the 2003 severe acute respiratory syndrome (SARS) outbreak.1 Emotional support and dissemination of reliable information can contribute to better coping with the psychological impact of a pandemic. | Multisource feedback was adopted to evaluate our QI initiative. These included: 1. Patients’ digital feedback on their overall hospitalisation experience and on the support materials in the iPad gathered through a semi-structured short survey form (box 1) incorporated in MyCare app. 2. Patients’ verbal feedback gathered and documented by the OPE staff. 3. Doctors’ serial feedback on patients’ changing FAQs over time. | Six hundred and thirty patients were isolated for suspicions of COVID-19 in SGH from 24 January to 19 March, of whom 24 had confirmed COVID-19 infection. Three hundred and thirty-nine (53.8%) of them were female. The median age of all patients was 40 (12–93), with males a decade older (37 (15–93) years vs 47 (12–93) years). Chinese accounted for 413 (65.5%) of them, Malays were 59 (9.3%) and other ethnicities made up 158 (25%). Confirmed cases had a median length of stay of 15 days in isolation while suspect cases had a median length of stay of 2 days.  App Usage: ....However, we were able to estimate usage of the device via ‘traffic log’ of the MyCare app and satisfaction level via an elec-tronic survey form in the MyCare app. The ‘traffic log’ was accessed remotely via a web analytic software. Out of a total of 224 patients admitted after the initial launch of the project on 28 February, 83 used MyCare app as of 19 March. Seven patients used the app to send messages to the healthcare staff. The messages consisted of medical queries, diet orders, requests for assistance to contact a relative warded in another isolation room and notes of appreciation to the healthcare team. |
| Natalie Stein & Kevin Brooks, 2017  Canada | Other: Health counseling or coaching | 16 weeks | Variable | Online | Not specified. | Primary outcome: Weight Loss: Each user’s weight loss was calculated as the difference between the final recorded weight and the baseline weight. The primary outcome in this study was percent weight change.  Other outcomes: Meal Quality: The HCAI classified individual foods and beverages as “healthy” if they promote weight control based on literature...Meals were recorded as “healthy” if they contained at least one healthy food and no unhealthy foods, and “unhealthy” if they contained at least one unhealthy and no healthy foods...Percent healthy and unhealthy meals at baseline were calculated by dividing the total number of healthy and unhealthy, respectively, meals logged by the total number of meals logged(including healthy, unhealthy, and neither) during the first week of logging. Final percent healthy and unhealthy meals were calculated based on the final week that users logged meals.  User Engagement: Duration of AI use was measured by the time, in weeks, between a user’s first and final use of the app. The number of conversations each user had with the app was also recorded.  Artificial Intelligence Acceptability and User Satisfaction: User satisfaction was assessed by an in-app user trust survey with four questions measuring (1) overall satisfaction, (2) net promoter score (NPS), (3) disappointment if HCAI were not offered, and (4) self-reported health improvement (Table 1).The satisfaction score (SS; Question 1) was the percentage that rated satisfaction as 6-10. Question 2 was used to calculate NPS by subtracting the percentage of detractors (score 0-6) from the percentage of promoters (score 9-10), as described by Krol et al [41]. The disappointment score (DS; Question 3) was the percentage that rated disappointment if the HCAI were not offered as 6-10. Health outcome score (HOS) was percentage of users responding that their health was “Much better than before” or “Somewhat better than before.” The SS, DS, and HOS were developed directly with the provider network | Data were analyzed for participants (N=70) who met engagement standards set forth by the Centers for Disease Control and Prevention criteria for Diabetes Prevention Program, a clinically proven weight loss program focused on preventing diabetes. Weight loss (standard error of the mean) was 2.38% (0.69%) of baseline weight. The average duration of app use was 15 (SD1.0) weeks, and users averaged 103 sessions each. Predictors of weight loss included duration of AI use, number of counseling sessions, and number of meals logged. Percentage of healthy meals increased by 31%. The in-app user trust survey had a 100%response rate and positive results, with a satisfaction score of 87 out of 100 and net promoter score of 47. |
| Natasha M et al., 2019  United States | Other: Mixed/hybrid - components of patient and peer navigation, also coaching | 5 months | Variable | Hybrid | Sequelae of trauma, including physical, social, and psychological elements, often persist long after patients have been discharged from the hospital. This can pertain to both substantial physical disabilities, as well as psychiatric illnesses that exacerbate these injuries in up to 45% of trauma patients.[1–7]Mental illness may slow recovery and potentially hinder satisfactory outcomes. Psychiatric illnesses have been associated with poor adherence to treatment recommendations, higher rates of complications, and greater risk for subsequent intentional and unintentional injury recidivism.[2,4,6–10] Patients with mental illness are also at risk for poor engagement, another factor possibly contributing to suboptimal recovery following orthopaedic trauma.[11,12]....The TSN was founded to address these concerns by improving engagement, increasing support, and creating a community of survivors. | Not specified clearly, but some information:  The aim of the present study is to evaluate patient satisfaction with TSN services and the impact of these services on patient perceptions about recovery. It is hypothesized that patients interacting with TSN will have greater satisfaction, optimism, and self-efficacy, indicating a constructive impact of this program and benefits of future widespread application. | On a Likert scale from 0 to 5, patients were highly satisfied (mean 4.24), with no differences based on TSN exposure. Patients exposed to TSN programming reported greater perceived likelihood of recovery: mean 3.73 vs 3.41 vs 3.38, Group 1 vs Group 2 vs Group 3 (P=.05) and regarding return to daily activities: 3.69 vs 3.49 vs 3.10,P=.003. Fifty-three percent of Group 1 patients exposed to TSN programming utilized peer relationships and 42% read the educational materials provided. Support groups were also popular, with 26% of patients attending at least 1 session. Patients who recalled utilization of TSN services were overall highly satisfied with these services, mean 4.42....Conclusion: Patients were overall highly satisfied with their hospital stay, with those exposed to TSN services reporting greater perceived likelihood of recovery and return to daily activities. Development of nontraditional services, including peer visitation and support groups, appears to enhance expectations about recovery.  3.1. Response rate: Four hundred eighty-five surveys were sent to patients, with 160responses for an overall response rate of 32.9%. Group 1 had the highest response rate, 35.5%, with 75 of 211 sent surveys being returned. Group 2 had the second highest, 34.1%, with 46 of 135patients returning surveys. Group 3 had the lowest response rate,28.1%, with only 39 of 139 patients responding to mailed surveys. Response rates were no different between groups(P=.57).  3.2. Demographics: The mean age of patients who received surveys was 43.0 years(SD=18.1), and 65.8% were male. The most common mechanism of injury was a motor-vehicle collision (MVC), (n=155,32%), followed by falls (n=154, 31.8%). The most prevalent injuries were to the tibia or ankle (n=148, 30.5%) and to the femur (n=129, 26.6%). Overall, patients were well matched. Survey groups were only dissimilar in terms of motorcycle collisions (MCCs), pedestrian collisions, and upper extremity injuries. Group 1, the TSN-exposed cohort, had substantially more operative upper extremity fractures (19%,P=.0011). All other demographic and injury variables did not reach statistical significance (Table 1). |
| Nichole Kang et al.,  2020  United States | Other: Case management navigators | Intended duration is 3-9 months | Minimum of one in person meeting per month. Meeting with navigator depends on engagement determined by clients’ needs and is established through an intake assessment that measures gaps in the ser- vices clients are receiving. | Hybrid | Past research on young adult populations vulnerable to health risks suggests that integrating information and com- munication technology (ICT) into case management may be a promising method to engage clients. | Not specified | SELPHI used ICT to support program implementation. Navigators contacted clients via phone, text, email, video call, and social media messaging and recorded their contact attempts in the database. SELPHI enrolled 59 clients; Navi- gators made a total of 799 contact attempts using ICT and an additional 150 in-person contacts. Most SELPHI clients engaged in at least one in-person meeting with their Navi- gator, with the average client meeting face to face between three and four times.  Within the 6-month implementation period, stakeholders learned several lessons about the role that technology can and cannot play in case management with expectant and parenting youth and found both successes and challenges with using ICT for these services.  The use of ICT gave Navigators more flexibility; all Naviga- tors reported that ICT either slightly or significantly reduced transportation and time barriers for clients. Additionally, Navigators stated that these modes of contact could be leveraged to provide more effective services.  Having multiple contact methods for each client avoids gaps in case management services. In the Navigator Feed- back Survey, three of the four Navigators identified lapsed phone service as a communication barrier for their clients and reported that this barrier was mitigated by access to multiple communication methods, |
| Olga Solonowicz et al.,  2022  United States | Patient | The reminder messages were sent starting from 14 days and continued up until 6 hours before the scheduled colonoscopy (see Fig. 1 for program content and timing). | Variable (see Fig. 1 for program content and timing). | Other: Text messaging program | Patient navigation programs have been shown to increase show rates and improve bowel preparation,5,6 however, hiring a full-time navigator carries a cost and can be labor intensive. More recently, digital navigation through short message service (SMS) has been shown to be an effective adjunct tool to improve bowel preparation quality and reduce no-show rates for patients undergoing screening colonoscopy.7–9...Further randomized data demonstrating the benefit of digital navigation are needed before widespread uptake of this technology | Our primary outcomes were endoscopy unit no-shows. This was defined by a patient not showing up for their scheduled colonoscopy without advanced notice or warning of cancellation.  Secondary outcomes included a composite of no-show and same-day cancellations, no-shows and cancellations within 7 days of the scheduled procedure, total completed procedures, bowel preparation quality, and patient satisfaction. The analysis of no-shows and cancellations was on the basis of all potential scheduled colonoscopies during the study time frame, whereas the analysis on bowel preparation quality was limited to patients who completed a colonoscopy during the study time frame. We defined an adequate bowel preparation on the basis of the Modified Aronchick Scale (adequate/excellent/good) and/or Boston Bowel Preparation Scale (BBPS)≥6 (with no individual segment<2). We used a composite assessment to define adequate bowel preparation, as the BBPS was not routinely used by all providers in our endoscopy unit until 2019, whereas the Modified Aronchick Scale was historically used by all providers. We then did a secondary analysis of bowel preparation outcomes limiting the analysis to patients who were scheduled for a colonoscopy for the indication of CRC screening or surveillance. | Completed Procedures and Bowel Preparation Quality: In total, 1146 of 1625 (71%) patients completed their scheduled colonoscopy in the study period. The intervention group was more likely to have a completed colonoscopy than the control group (73% vs. 68%;P=0.03). Of the completed colonoscopies, there were no significant differences for diagnostic or screening indications between the 2groups (P=0.94). There also was no significance in adequate bowel preparation rates for all completed colonoscopies (diagnostic and screening/surveillance) (89% vs. 86%;P=0.465). However, when limited to screening and surveillance colonoscopy only, the rates of adequate bowel preparation were significantly higher in the intervention group (93%) compared with the control group (88%;P=0.04).  Patient Satisfaction: Satisfaction surveys of 1724 patients were analyzed(intervention group, n=899; control group, n=825). The questions involved understanding of the bowel preparation, feeling prepared for the colonoscopy, and feeling pleased with the overall care. The mean patient satisfaction score was similarly high in both the intervention group and the control group (4.56/5 vs. 4.59/5). In addition, a higher percentage of the patients in the intervention group compared with control group “strongly agreed” that they understood the instructions, felt prepared for their colonoscopy, and were pleased with their overall care (77.8% vs. 74.4%;P=0.0023). |
| Owen Katalinic et al.,  2013  UK | Other: Provides various types of interventions through the iPad and Intel health guide. See description of intervention | The telehealth devices were loaned to a total of 102 patients for different lengths of time, depending on clinical needs, but typically for about 3 months | Variable | Online | Not specified | The home telehealth project set out to answer the following questions:  1. Does the use of home telehealth improve access to services and clinical outcomes, particularly for people living in regional and rural areas?  2. Are videoconferencing and remote patient monitoring acceptable modalities that integrate well with established clinical services?  3. What are the relative advantages and disadvantages of the technologies used in the trial?  4. What does it take to implement and support the technology? | The main findings were:  1. Telehealth can play a useful role in improving accessto services, particularly for those who live in ruralareas.  2. Detailed planning and high-level support for telehealth is essential in establishing a framework for telehealth.  3. Rapid resolution of problems is essential in maintaining clinician engagement.  4. Both patients and clinicians readily accept and learn how to use new technologies, particularly where using them saves significant amounts of time.  5. Usability and ease of use are critical in ensuring theacceptance of the technologies.  6. The lack of broadband Internet in some regional and rural areas rules out the use of telehealth in those areas.  7. Internet bandwidth and latency plays a critical role in the quality and experience of the video conference.  8. Latency and quality of video conferencing over mobile broadband networks is highly variable, depending on the local signal strength.  9. Technical problems that affect the Wide Area Network can have serious effects on the delivery of home-based telehealth services.  10. Initial concerns that equipment would be lost or stolen from patient’s homes have proved unfounded. To date, there have been no losses of home telehealth equipment.  11. Age does not appear to be a barrier to using home telehealth technologies and video conferencing technology.  12. The use of iPads for clinical therapeutic purposes hassignificant potential and is well accepted by cliniciansand patients. This will continue to be explored. |
| P. W. Colson et al.,  2020  United States | Peer | 12 months | Variable | Other: Online support groups via social media and SMS messaging | Despite notable successes in HIV prevention over the past three decades, the annual number of new HIV infections in the United States (US) has remained relatively stable since 2013 at close to 39,000 per year. Black men who have sex with men (MSM) are disproportionately impacted, comprising 25% of new diagnoses in 2018 and experiencing an estimated lifetime risk of HIV diagnosis of one in two, compared to an estimated lifetime risk of one in 11 among White MSM and one in 524 among heterosexual men in the US. | The primary outcome, self-reported adherence, was measured at follow-up visits using the 3-item Wilson adherence scale.Participants were asked how many days in the last 30 days they missed taking PrEP, how well they did in the last 3 months taking PrEP the way they were supposed to (1–6 scale of “Very Poor” to “Excellent”), and how often they took their PrEP in the way they were supposed to in the last 3 months (1–6 scale of “Never” to “Always”). These three items were linearly transformed to create a scale of 0–100, and good adherence was defined as ≥ 57 out of 100.  The study protocol called for the collection of dried blood spots (DBS) for all participants at clinical visits associated with the 6- and 12-month time points. DBS specimens were extracted from routine blood draws collected at laboratory visits and analyzed for tenofovir diphosphate (TFV-DP)levels. Optimal adherence was defined as TFV-DP ≥ 700 fmol/punch. | A total of 204 participants were enrolled and randomized; 35% were lost to follow-up. PrEP adherence was 30% at 12-months; no intervention effect was observed (p = 0.69). Multivariable regression analysis found that lower adherence was associated with low education and depressive symptoms. We found that an enhanced adherence intervention did not improve PrEP adherence. Findings point to the need for innovative methods to improve PrEP adherence among Black MSM and TGW.  Because DBS were only able to be collected among 46 of 128 participants (35.9%) interviewed at the 6-month time point and 47 of 132 participants interviewed (35.6%) at the 12-month time point, DBS were not utilized to estimate the primary adherence outcome measure. The sensitivity and specificity of self-reported compared to DBS adher- ence at 6-months were 60.9% and 73.9%, respectively. The sensitivity and specificity of self-reported compared to DBS. adherence at 12-months were 45.5% and 88.0%, respec- tively. Notably, there were 6 false negatives comparing self- reported to DBS adherence at 6-months (i.e. participants self-reporting non-adherence but DBS results indicating adherence) and 3 false negatives at 12-months. The median number of medical visits attended for the 93 participants who attended subsequent medical visits was 1 visit (IQR = 1 visit) and the median number of medication bottles picked up for the 125 participants who picked up medication was 3 bottles (IQR = 5 bottles); there was no difference by study arm (p = 0.61 and p = 0.32, respectively). Examining self-reported adherence among all partici- pants over the 12-month study period, 52.9% were adher- ent at 3 months, 42.2% at 6 months, 35.8% at 9 months, and 32.4% at 12 months. Approximately 30% of participants in both arms were adherent, and 60% were non-adherent as per 57% threshold. In both arms, non- adherent participants comprised two roughly equally sized groups of participants who either reported little or no use of PrEP or were lost to follow-up and therefore assumed to be non-adherent.  Sixty of the 101 enPrEP participants (59.4%) met at least once with a peer |
| Paula Anne Newman-Casey et al., 2018  United States | Peer | No specific intervention in the study | No specific intervention in the study | Online | No specific intervention in the study | Access to and experiences with e-health technologies. We also aimed to evaluate whether technology use differs by medication adherence status and whether technology use differs between older and younger glaucoma patients. | Eighty percent had good technology access. Seventy-three percent of subjects with greater technology access wanted online glaucoma information and yet only 14% of patients had been directed to online resources by physicians. There was no relationship between technological connectivity and adherence (p=0.51). Nonadherent patients were younger (mean age 58 years vs. 66 years for adherent patients, p = 0.002). Non-adherence was associated with negative feelings about online searches (68% vs. 42%, p = 0.06). |
| Paula Anne Newman-Casey et al., 2020  United States | Other: Coaching | 7-month program | Participants then met with the glaucoma coach for 3 in-person and 5 over-the-phone coaching and education sessions over 6 months | Hybrid | The SEE program, in particular the MI component, is grounded in self-determination theory | Change in electronically monitored medication adherence.  Adherence was calculated as the percentage of doses taken on time of those prescribed. | A total of 48 participants were enrolled. The participants were 54% male, 46% white, and on average 64 years of age (standard deviation [SD], 10.8 years), with an average worse-eye mean deviation (MD) of e7.9 dB (SD, 8.8 dB). Those completing the SEE program (n 1⁄4 39) did not differ significantly from those who dropped out (n 1⁄4 9) on gender, race, age, MD, or baseline adherence. Medication adherence improved from 59.9% at baseline to 81.3% (P < 0.0001) after completing the SEE program. Ninety-five percent of participants showed an improvement in adherence (mean relative improvement, 21.4%; SD, 16.5%; range, e3.2% to 74.4%; median, 20.1%). Fifty-nine percent of participants showed adherence of >80% on completing the SEE program. |
| Ponrathi Athilingam et al.,  2016  United States | Other: Mobile Platform/App for Patient with Heart Failure - this mHealth platform acts like a health coach for patients. | For beta testing, participants were asked to use the app for 2 hours before giving feedback and filling surveys/questionnaires. | Not specified, varies based on patient usage | Online | Patient-centered approach.  The theories that were used during the design of the mHealth app include: Mayer’s Cognitive Theory of Multimedia Learning, Sweller’s Cognitive Load Theory, Instructional Design Approach utilizing a Pedagogical Agent, and problem-based learning. | The validated Self-efficacy for Exercise Scale (Cronbach’sα= .92) was modified and used to measure self-efficacy.  Through a survey -- The assessment during beta testing focused on whether the learner had acquired adequate information from the tutorial, familiarity with the content, ease of use of the app in general, and whether the design was simple enough to learn and navigate. Participants were asked to indicate refinement needs to improve usability of the mobile app. The participants answered survey questions asking whether the individual learned any new information from the content provided in the app and how likely they were to recommend the app to other patients with HF. he survey also included questions on mobile phone ownership, type of mobile phone owned, use of mobile phone for texting and obtaining health information, how well they use the mobile phone, and demo-graphic and clinical information. Four clinicians provided feedback on the content of educational materials. | The 10 participants who completed the beta testing had a mean age of 63 years and ranged from 43 to 81 years of age, of whom 60% were 65 years or older. As shown in Table 2, all10 of the participants were employed full time or part time, 60% were men, 70% were white, and 40% lived alone....All 100% (N = 10) of the participants owned a mobile phone (50% were smartphones), 50% used text messaging, and 60% reported using a mobile phone very well and 40% fairly well.  All participants (100%) reported moderate to extreme confidence in using the app, 95% were very likely to use the app, 100% re-ported the design was easy to navigate, and content on heart failure was appropriate. Almost 70% of participants reported learning new in-formation from the app on HF education and that the information provided in the app was found to be a good re-fresher and of value to patients with newly diagnosed HF for whom they will recommend the app. Having the information accessible on a mobile phone was reported as a health coach or companion promoting persistent engagement to improve health outcomes by all patients. All participants owned a mobile phone, 50% owned smartphones. Those who did not own smartphones (50%) were also able to navigate the app easily.  Clinicians (n = 4) rated the content's congruence with current medical practice and clinical guidelines. Nurses (n = 4) re-ported that having HF education accessible on a smartphone would help to stop or reduce the revolving-door phenomena of frequent readmission. Clinicians and nurses reported that the design was engaging and easy to navigate.  Taking all of the test results into consideration, no additional changes were made to the app. The mHealth app is ready to be tested in a pilot clinical trial. |
| Qiwei L. Wua et al.,  2020  Ireland | Patient | N/A. This study is not about a specific intervention, but about e-communication of patients with clinicians via secure messaging, emailing, and data sharing. | N/A. This study is not about a specific intervention, but about e-communication of patients with clinicians via secure messaging, emailing, and data sharing. | Online | In order to identify factors affecting patients’ willingness to use e-communication with cancer care providers, this investigation embraces a key premise of social cognitive theory (SCT) [12] that posits that an individual’s behavior is a function of personal factors and environmental influences. Given past research, we expect personal characteristics (e.g., higher education, health status, younger age) may influence one’s e-communication with providers [13,14]. Cancer patients’ interactions with their environments would include their relationships with health care providers as well as their relationships with technology. Some research indicates each may independently influence e-communication with clinicians [15,16]. | Study measures:  1. Age  2. Gender  3. Education  4. Physical  5. Mental Health  6. Cancer History  7. Health-related information-seeking behaviour  8. Track  9. Patient-centred communication  The outcome variable, e-communication with clinicians, was the sum of whether in the past year respondents had texted, emailed, and/or electronically shared information with a health care provider (scale range 0–3). To answer the research question, predictor variables were grouped into 3 categories based on the premises of SCT—(a) personal and health-related (age, education, race/ethnicity, gender, time since cancer diagnoses, physical and mental health status), (b) past experiences with health care providers (how many visits with providers over past 12 months, quality of past communication experiences), and (c) use of technology for health-related purposes (using the Internet to seek health information, using technology to track health data). | In this pre COVID-19 sample, 42 % respondents (N = 252) did not engage in any type of electronic communication (e.g., emailing, texting, data sharing) with providers. In multivariate analyses, predictors of more electronic communication with clinicians included frequency of seeking health-related information online (ß = .267, p<.001) and better communication experiences with clinicians (ß = .028, p = .034), while no demographic variable showed significance. The technology use variables (online health information seeking, health tracking) were significantly higher predictors of electronic communication with clinicians  (triangle sign - DR2 = .142, p < .001) than was past experiences with clinicians (DR2 = .029, p = .016).  Participant characteristics: Most participants were females (N = 344, 58 %) and reported good to excellent physical (N = 443, 74.7 %) and mental health (N = 525, 88.5 %) (Table 2). Education levels varied, and time since cancer first diagnosed cancer ranged from 0 to 87 years (M = 13.83, SD = 13.39). Approximately 42 % respondents (N = 252) had not engaged in any e-communication with providers.  Statistical analyses: In bivariate analyses, e-communication was higher among the younger (r = -.198, p < .001) and more educated respondents  (r = .224, p < .001), as well as those who more often sought health  information online (r = .480, p < .001), tracked health data (r = .430,  p < .001), and who visited providers more often over the last 12  months (r = .173, p < .001)...In multivariate analysis, demographic and health factors (Model 1) explained 7.4 % of the variance in patients’ e-communication, with education being the only significant predictor (ß = .171, p = .001). Variables related to past experiences with providers (Model 2) explained an additional 2.9 % of the variance in patients’ e-communication. While education remained a significant predictor (ß = .179, p < .001), the influence of past patient-centered communication experiences (ß = .026, p = .069) and the frequency of seeing doctors (ß = .071, p = .061) did not reach statistical significance. Technology-use factors (Model 3) explained an additional 14.2 % of the variance in the outcome variable. In this model, patients’ online information-seeking  behavior (ß = .273, p < .001) and past patient-centered communication experiences (ß = .028, p = .031) predicted their e-communication, with marginal effects associated with the frequency of seeing doctors (ß = .057, p = .104) and using electronic methods to track health data (ß = .186, p = .051) (see Table 3 for full regression results). |
| Rishi J. Khusial et al.,  2020  United States | Other: Self-managment | Participants were included over a period of 4 months, whereas we used a fixed end date for all participants, resulting in a varied follow-up duration (3-6 months) | Variable | Online | Self-management programs have beneficial effects on asthma control, but their implementation in clinical practice is poor. Mobile health (mHealth) could play an important role in enhancing self-management.  Patient adherence to self-management programs is low, with only 20% of people reporting the use of an action plan. Self-management tasks are often regarded as burdensome and time consuming, whereas patients indicated that they would prefer different data to be added to their asthma action plan | The primary outcome was asthma control; secondary outcomes were exacerbations, quality of life, and technology acceptance. | In study 1, asthma control improved in the intervention group compared with controls (Asthma Control Questionnaire difference, 0.70; P = .006). In study 2, asthma control improved by 0.86 compared with baseline (P = .007) and quality of life by 0.16 (P = .64). |
| Robert D Reid et al.,  2011  UK | Other: Self-managment | 6 and 12 months following hospitalization | Variable | Online | Not specified | Physical activity levels were objectively measured via pedometer in all participants 6 and 12 months following randomization. Self-reported leisure-time physical activity and heart disease health-related quality of life were assessed in all participants at the baseline and 6- and 12-month visits.  The primary outcome was the average number of steps per day measured by pedometer (Yamax DIGI-WALKER, Yamasa Tokei Keiki, Tokyo, Japan) over a 7-day period.  A modified version of the Godin Leisure-Time Exercise Questionnaire9 was used to gather self-reported data concerning leisure-time physical activity.  Heart disease health-related quality of life was measured using the 27-item MacNew instrument,10,15 which measures three quality of life domains (emotional, physical, and social) as well as global quality of life. | The CardioFit internet-based physical activity expert system significantly increased objectively measured (p = 0.023) and self-reported physical activity (p = 0.047) compared to usual care. Emotional (p = 0.038) and physical (p = 0.031) dimensions of heart disease health-related quality of life were also higher with CardioFit compared to usual care.  Preliminary analyses showed that age, physical activity level prior to hospitalization, first hospitalization for CHD, and being employed were significantly related to pedometer outcomes at follow up; these variables were used as covariates for subsequent analyses of pedometer data. The repeated measures analysis of covariance for pedometer-measured steps per day showed a main effect only for group. The number of steps per day was higher in the CardioFit group compared to usual care.  Preliminary analyses showed that physical activity level prior to hospitalization, and first hospitalization for CHD were significantly related to self-reported physical activity at follow up; these variables were used as covariates for subsequent analyses related to self-reported activity.  Preliminary analyses showed that the number of comorbidities was significantly related to heart disease health-related quality of life at follow up;  For the emotional and physical subscales of the MacNew survey instrument, a significant group effect emerged; participants in CardioFit had higher quality of life scores than usual care across time. For the social subscale of the MacNew survey instrument, group, time, and group×time interaction effects were not significant. |
| Russell E. Glasgow et al.,  2003  United States | Other: Coaching, online self management/tracking, & peer support | Not specified. | Variable | Online | The intervention components were grounded in two theoretical frameworks. Self-efficacy theory provided the theoretical framework for Tailored Self-Management. Social support theory was the theoretical framework for the Peer Support intervention. | Effectiveness was evaluated by improvement from base-line to the final assessment 10 months later using multiple measures within each of three different domains: behavioural, biological, and psychosocial outcomes. Self-report measures were completed primarily by mail or in-person and collected by research staff. Baseline measures were collected immediately prior to randomization  Dietary outcomes were assessed by improvements on theKristal Fat and Fiber Behavior (FFB) scale and the Block/NCI Fat Screener.  Other behavioral outcomes were assessed by a measure of the percentage of diabetes care guidelines that patients met and a measure of physical activity.  Biological outcomes were evaluated by changes in HbA1C and lipid ratios.  Psychosocial outcomes were assessed by the Diabetes Support Scale and the Center for Epidemiologic Studies–Depression scale | Website use measured by log ons varied highly across time and conditions, despite our efforts to keep all participants involved with the website throughout the intervention. As seen in Fig. 1a and b, there was consistently greater website use over the first 3-month period for all conditions. Usage dropped off gradually across all conditions during the second 3 months, with the lowest usage rates occurring during the 7- to 10-month period. In addition, across all time periods of the study both the PS and the TSM conditions resulted in more frequent log ons than those not receiving these interventions, with the PS conditions resulting in the most frequent website usage at all time points.  Ten-month assessment results revealed significant improvements from baseline across conditions on the majority of outcomes (Table 2), and the magnitude of these improvements was approximately the same as that seen at earlier follow-ups [22]. Improvements were largest for the targeted dietary outcomes of the Kristal FFB and the Block Fat Screener, next largest for the psychosocial outcomes (perceived barriers and support; depression scores), and more modest for other behavioral outcomes (e.g., improvements in medical care) and biological outcomes (e.g., 12 mg/dl reduction from baseline in cholesterol).As can be seen in Table 3, the pattern of changes generally favored the TSM and PS conditions compared to conditions not receiving these components. However, multivariate analyses to evaluate potential differences between treatment conditions failed to reveal significant incremental effects of either TSM or PS at the 10-month assessment, with the exception of the psychosocial outcomes. Follow-up univariate analyses revealed that this overall effect was due to the PS conditions producing significantly greater improvement on the Diabetes Support scale than among those not in these conditions. Because of the relatively good baseline levels on some measures such as HbA1c, we also conducted analyses of “differential outcome by baseline level” [45,46] in which we included interaction terms between treatment and baseline level on the relevant dependent variable (using median splits). These analyses failed to reveal any differential effects. |
| Ryan P. et al., 2017  UK | Peer | 9-month pilot intervention | Meet with peer navigator in person at least twice in the first month after enrolment, then variable phone call or text messages on an as needed basis  smartphone application prompted participants to complete brief surveys via the smartphone inter- face twice daily | Hybrid | The mPeer2Peer intervention featured two components, mHealth and peer navigation, which were based on the situated Information, Motivation and Behavioral Skills (sIMB) model of care initiation and maintenance. | Assessed the acceptability of the intervention components using qualitative analysis of in-depth interviews conducted with study participants and peer navigators  Acceptability of the intervention was evaluated with one-on-one, in-depth interviews with the first 12 study participants and the 3 peer navigators. | Of 19 patients enrolled in the study, 17 participated for at least 2 months and 15 completed the entire 9-month study protocol. The acceptability of the peer navigation intervention was rated favorably by all participants interviewed, who felt that peer support was instrumental in helping them re-engage in HIV care. Participants also responded favorably to the smartphone application, but described its usefulness mostly as providing reminders to take medications and attend appointments, rather than as a facilitator of patient navigation. |
| Ryan P. et al.,  2017  UK | Peer | 9 month study protocol | Variable | Other: In-person, app (with education), text messages, calls, etc. | In large cohort studies, people who inject drugs have been demonstrated to have inferior virologic outcomes and higher mortality than other patients receiving ART [9]. To ensure maximal benefit for these populations, social support and/or care coordination strategies are needed to address the specific barriers encountered by people who use drugs when they receive HIV care....Electronic health (eHealth) and mobile health (mHealth) tools hold promise to move these efforts forward.  The mPeer2Peer intervention featured two components, mHealth and peer navigation, which were based on the situated Information, Motivation and Behavioral Skills (sIMB) model of care initiation and maintenance [28]. In this theoretical framework, relevant information, motivation, and behavioral skills interact to determine engagement in care and HIV-related behaviors and outcomes. In the context of this study, the IMB model is influenced by moderating patient and peer factors, as well as structural/health systems and clinical domains, as illustrated in Fig. 1. | Qualitative evaluation: Acceptability of the intervention was evaluated with one-on-one, in-depth interviews with the first 12 study participants and the 3 peer navigators...Semi-structured interview guides were designed to elicit perceptions about the usefulness of each intervention component, specific needs met by the intervention, and the ease of use of the smartphone application. | Between September 2013 and November 2014, 19 individuals were enrolled and randomly assigned to receive the mPeer2Peer intervention. The baseline characteristics of the intervention recipients are shown in Table 1. The study sample was reflective of the population living with HIV in Baltimore, i.e., predominantly Black, male, and low-income, with a median age of 49. 3 years. Most participants reported they were taking antiretroviral therapy at the time of enrollment, yet all but one had an HIV viral load greater than 1000 copies/mL.  Study retention and losses to follow‑up: Of the 19 patients randomized into the intervention group, 15 (78.9%) were followed for the entire 9-month study period. Two participants were immediately lost to follow-up and had no contact with the study team after the enrollment visit; two others were lost to follow-up after month 2 and 6, respectively. Collectively, these 19 participants contributed 143 person-months of follow-up after enrollment. |
| S. Wilson Beckham et al., 2021  UK | Other: Peer navigation (through peer educators) combined with other navigation components (outreach nurse | 18 months | Not specified, but variable | Other: Combination of in-person (face-to-face) and text messages | Community empowerment models for HIV prevention are effective at reducing the odds of HIV infection and improving condom use among FSW [22]. Such models often take a multi-pronged approach to HIV prevention, including elements of community-led mobilization to address social and structural barriers (such as stigma) to HIV prevention, treatment and care, as well as to pro-vide peer education and service navigation, condom distribution, and HIV/STI screening. As sexual and RH knowledge and access to care and contraception all in-fluence FP uptake, community empowerment approaches could theoretically improve FP use. | Family planning use: The primary outcome was self-reported current family planning use measured at the 18-month follow-up survey. Participants were asked if they currently used various modern contraceptive methods...“Current family planning use ”was defined as use of at least one of the afore-mentioned modern methods.  Program exposure: Program exposure was measured at the 18-month follow-up survey. General program exposure was dichotomized any/none and was defined as at least one of the following: 1) any attendance of the monthly seminars participant-organized weekly meetings, or walk-in visits held at the Shikamana DIC; 2) obtaining condoms at the DIC, or 3) getting tested for HIV at the DIC. Family planning program exposure was defined as attendance of a seminar, workshop, or meeting specifically about FP.  Reproductive and family planning history: Additionally, the baseline survey captured historical FP use...The 18-month follow-up also asked participants about their reproductive histories... and use of ANC services....Since formative qualitative work previously indicated that some FSW were not accessing ANC services because they were not accompanied by husbands for couples HIV testing [10], the survey also asked if participants were accompanied by a male partner, told to bring a partner, or did not or could not access services due to inability to bring a partner. | Among the 339 participants with follow-up data on family planning, 60% reported current family planning use; 6% reported dual use of modern contraception and condoms; over 90% had living children; and 85% sought antenatal care at their most recent pregnancy. Among the 185 participants in the intervention arm, the adjusted relative risk (aRR) of family planning use among female sex workers who reported ever attending the Shikamana drop-in-center and among female sex workers who reported attending a family planning-related workshop was respectively 26% (aRR 1.26 [95% Confidence Interval (CI): 1.02–1.56]) and 36% (aRR 1.36 [95%CI: 1.13–1.64) higher than among those who had not attended.  Participant characteristics: Just over half of participants were living with HIV. Nearly 30% of participants had ever attended the Shikamana center and only around 17% of participants had ever attended a specific family planning session.  Prevalence of family planning use: Nearly 84% of participants reported ever using modern FP (Table3). Of all study participants, 61% reported current modern family planning use, though 69% of participants who reported that they were not currently trying to become pregnant at the follow-up survey were currently using contraception. As such, over 31% of participants not currently trying to become pregnant demonstrate unmet need for family planning.  Bivariate analysis of modern family planning use: Community, number of clients a week, and history of FP use were significantly associated with current FP use(Table4). Of those currently using FP, nearly 60% of participants were from the intervention community, whereas more than half of those not currently using family planning were from the comparison community. About 45% of participants had two or more clients a week. A larger proportion of those currently using family planning (52%) had two or more clients a week com-pared to those not currently using family planning(35%). Almost all (n= 192) of the participants who re-ported current FP use also reported ever use at baseline. Sixteen of the 56 participants (29%) who stated they had never used family planning at baseline reported current use at the 18-month follow-up survey. Almost half of participants reported inconsistent condom use over the past 30 days. |
| Saeed Moradian et al., 2018  Canada | Other: Self monitoring and management | Not specified | Not specified, variable | Online | Not specified | Participants’ experiences with the system evaluated through qualitative interviews and questionnaires  aimed to elicit feedback and identify design, functionality, and usability issues. In addition, participant experiences, thoughts, feelings, and satisfaction with the ASyMS were assessed by an audiotaped, semistructured, face-to-face qualitative interview with participants and through completing a short questionnaire (modified Telehealth Acceptance Measure, TAM), immediately after usability testing sessions | Results from the posttest questionnaire indicated that 80% (8/10) of participants had great motivation to use the ASyMS, 70% (7/10) had positive perceptions of the successful use of the ASyMS, and all (10/10, 100%) had a positive attitude toward using the ASyMS in the future. |
| Sean Arayasirkul et al., 2020  United States | Other: Digital (HIV) care navigator | 6 months | Not specified, but variable based on interaction. | Other: mHealth (mobile health) using SMS/text messaging. | Not clearly specified. | Data for this analysis were collected using computer-assisted self-interviewing (CASI) surveys. Instruments collected self-reported sociobehavioral information pre- and postintervention at baseline and 6 months.  HIV Care Continuum Outcomes Self-reported HIV care outcome data were collected using CASI surveys. In accordance with HIV care goals designated in 90-90-90 objectives, we dichotomized outcomes regarding whether participants received primary HIV care within the last 6 months (yes/no, 1/0), whether participants were taking ART (yes/no, 1/0), and whether participants were virally suppressed (eg, had a viral load of 200 copies/mL or less) (yes/no, 1/0). | Table 1 presents baseline sociodemographics and HIV care continuum outcomes for the Health eNav sample overall (n=120) and according to the intervention completion status. Except for incarceration and recent receipt of primary HIV care, characteristics according to the intervention completion status were not significantly different from those of the overall sample. The mean age of the participants was 27.75 years (SD 4.07). Most participants (103/120, 85.8%) identified as men. The sample was racially/ethnically diverse, with most participants identifying as Hispanic/Latinx, followed by white, multiple races, and black/African American, and few identifying as Asian or American Indian/Alaska Native. About half (68/120, 56.7%) of all participants completed some college education, yet most lived in unstable housing and had a monthly income of US $1300 or less. Recent incarceration was less likely in participants who completed the intervention than those who did not complete the intervention (11.67% vs. 26.67%, χ21=4.36, P=.04).  In terms of baseline HIV care continuum outcomes, majority (99/120, 82.5%) of the participants had recently received primary HIV care, yet this was more likely in those who completed the intervention than in those who did not complete the intervention (54/60, 90% vs 45/60, 75%; χ21=4.68, P=.03) (Table 1). The 6-month follow-up surveys were completed by 73.3% (88/120) of participants (Table 2), and these participants were not characteristically different from the overall sample at baseline. Table 2 presents the longitudinal results from the GEE models. After analyzing HIV care continuum outcomes over the 6-month study period, we observed that participants had increased odds of viral suppression at 6 months compared with baseline. We observed no statistically significant additive or multiplicative interactions on comparing outcome effects over time according to intervention completion. However, on testing for stratum-specific effects, we found that viral suppression increased over time among those who completed the intervention (83.89% probability of viral suppression at 6 months vs 69.60% probability of viral suppression at baseline; probability difference 14.29%, 95% CI 2.66%-26.41%). No corresponding stratum-specific difference in viral suppression was observed among those who did not complete the intervention.  Our findings suggest that digital HIV care navigation is effective at promoting viral suppression at post-test compared with pretest. |
| Sean Arayasirkul et al., 2020  United States | Other: Digital Care Navigator (not clearly specified). | 6 months | - Variable, participants were able to communicate with their digital HIV care navigator via SMS text message on an open schedule | Other: mHealth (mobile health) using SMS/text messaging. | Motivational interviewing has been widely applied to in-person delivered health behavior change interventions; however, mobile health (mHealth) interventions are beginning to adopt and expand the reach of MI in health promotion practice with the use of mobile phones and digital platforms (Zunza et al., 2017). | Not clearly defined, but related:  Study outcome variable: "Change talk was our primary dependent variable or outcome in this analysis and refers to participant statements that indicate an inclination or reason for change and can vary in the strength of commitment toward enacting that change...."  "In order to quantify text message data that were qualitatively coded, the number of times each MI skill was used across all text messages for each participant was counted and summed.....participants in the 0 to 32rd per-centile of a particular MI skill were considered to have a low level of that skill, those in the 33rd to 65th were considered to have a moderate level, and those in the 66th percentile and above had a high level. Finally, we created a composite MI variable, in which we classified participants according to whether they received 0 to one type of MI skills, two types of skills, three types of skills, or all four types of skills....These models accounted for overall texting engagement (i.e., number of texts sent and received) as a predictor of change talk counts. All statistical analyses were conducted in Stata 14 (Stata Press, 2015). Comparisons producing p values less than .05 were considered statistically significant." | Across sociodemographic, open-ended questions and affirmations were the most frequently utilized MI skills; reflective listening and summarizing were less common. While 50% or less of Black, Hispanic, or White participants received reflective listening, more than 60% of participants with multiple or “other” race/ethnicity received this skill.  Results show that as the MI skill levels increase, the median instances of change talk tends to increase.  Based on adjustment of total number of texts sent/received, "high levels of OARS are associated with an increased count of change talk compared to low levels (p< .01 for all comparisons)."  This study found that high levels of all four MI skills—and moderate levels of open-ended questions only—were associated with more change talk compared to low levels. Using three or more MI skills was associated with change talk as well. These data suggest that delivery of MI through text messaging is not only efficacious in promoting change talk but also feasible for both the interventionist and the individual participant. |
| Shantanu Nundy et al., 2013  Canada | Patient | 30 days | Variable | Other: Automated text messages | Not specified. | The pilot was designed as a single-arm prospective study. The primary endpoint was change in the Self-Care of Heart Failure Index (SCHFI), a well-described measure of self-management in heart failure [18], which was administered at enrollment and at the end of the 30-day intervention. In addition, a mobile phone usage survey was administered on enrollment [19], and demographic and clinical data were obtained through chart review. At the completion of the intervention, a telephone-based patient experience survey, including Likert-scale and open-ended questions, was administered [19].  Additional info: Per the most recent scoring procedure [18], raw scores from the SCHFI were tabulated into standardized 100-point scales: maintenance, management, and confidence. Preintervention and postintervention scores for each scale were compared using paired t tests. Individual items were compared using Wilcoxon rank sum tests. Stata version 11 was used for the analysis (StataCorp LP, College Station, TX, USA). | Of 51 patients approached for recruitment, 27 agreed to participate and 15 were enrolled (14 African-American, 1 White). Barriers to enrollment included not owning a personal cell phone (n=12), failing the Mini-Mental exam (n=3), needing a proxy (n=2), hard of hearing (n=1), and refusal (n=3). Another 3 participants left the study for health reasons and 3 others had technology issues. A total of 6 patients (5 African-American, 1 White) completed the postintervention surveys. The mean age was 50 years (range 23-69) and over half had Medicaid or were uninsured (60%, 9/15). |
| Sharon M. Bigelo et al., 2021  United States | Patient | Not specified, variable | Not specified, variable | Online | Not specified - The COVID-19 pandemic is disproportionately affecting low- income, minorities, and a broad range of other vulnerable populations, including cancer patients. Cancer patients are experiencing deferred treatments, delayed evaluation, interrupted ancillary services, and increases in barriers to care. Navigation programs have been implemented to address inequitable access to cancer care. | Common risk factors, Statistical differences between Spanish-speaking and non-Spanish-speaking patients, follow-up visits requitred | The program engaged with 586 adult cancer patients over 1459 encounters. The most common risk factors included distance (59.7%), complex care (48.8%), and new treatment start (43.5%). The most common interventions were core education (69.4%), emotional support (61.2%), and education (35.7%). Statistical differences were found between Spanish-speaking (n = 118) and non-Spanish-speaking patients (n = 468). While Spanish-speaking patients had fewer risk factors (1.95 vs. 2.80, p ≤ .0001), they had nearly double the number of visits (4.27 vs. 2.04, p ≤ .0001) and 69% more interventions (8.26 vs. 4.90, p ≤ .0001). Many patients (42.7%) required follow-up visits. |
| Silvia Cacho-Elizondo et al., 2013  United States | Other: Mobile coaching service (SMS/MMS-text/video coaching) | N/A - No intervention actively tested - study about intention to adopt mobile coaching intervention for smoking cessation. | N/A - No intervention actively tested - study about intention to adopt mobile coaching intervention for smoking cessation. | Hybrid | In France, almost 60,000 deaths each year are directly attributable to smoking, which is the primary cause of avoidable premature death, and the problem of nicotine addiction continues to grow despite efforts to curb it. Across the whole French population aged 15-75, the proportion of daily smokers rose from 26.9% to 28.7% between 2005 and 2010, and cigarette sales saw a slight upturn between 2008 and 2009 (from 53.6 billion to 55 billion packets) after dropping significantly between 2001 and 2004 (from 82.5 billion to 54.9 billion, due to substantial increases in the price of tobacco products).  When an information technology-based service is in the early stages of diffusion, as is the case for mobile coaching services to help people stop smoking, the intention to adopt appears a more appropriate object of study than adoptive behavior (Hong & Tam, 2006). This is why we seek to explain the intention to adopt rather than adoptive behavior....The Technology Acceptance Model (TAM) constructed by Davis (1989) is an adaptation of the theory of Reasoned Action (Fishbein & Ajzen, 1975) designed to model the intention to adopt information systems.  Discussion: The intention to adopt the text message-based mobile coaching service to help stop smoking was tested with a mostly young target. This is precisely the group with the greatest risk of developing tobacco-related illnesses in the long term, since the lower the age at which people start smoking, the higher their risks of serious tobacco-related illnesses. Yet the study observed that these young smokers do not always intend to stop smoking in the short term. It is important to find innovative ways to motivate them to try and start the process of giving up smoking. With this in mind, mobile services can play an important role because young people are generally heavy users of cell phones and generally find new technologies attractive (Syed & Nurullah, 2011). | Below is the analysis of the data collected through the face-to-face survey with smokers....Six 5-point Likert scales from “totally disagree” to “totally agree” were used to measure the variables in the model...Six constructs were measured:  Intention to Adopt the Mobile Coaching Service (BINTENT): This scale consists of three items: (1) I intend to use this mobile coaching service to stop smoking in the future. (2) I would be prepared to use this mobile coaching service to stop smoking in the future. (3) To stop smoking, I will use this service.  Vicarious Innovativeness (DSI):...a scale to measure consumers’ tendency to be among the first to try out new products in a specific field....final modified scale consists of 6 items measuring involvement, intention to adopt, perceived knowledge, ease of use, need for change and need for cognition. In addition to these items, we include a price item (I will adopt a mobile coaching service if the price is right).  Social Influence (SOCIAL):...three items were used: (1) People who are important to me would want me to use this mobile coaching service. (2) People who influence my behavior would think I should use this mobile coaching service. (3) People whose opinions I value would prefer me to use this mobile coaching service.  Perceived Monetary Value (MONEY):...three items were used: (1) I expect this mobile service would have a reasonable price. (2) This mobile service would offer good value for money. (3) I believe that at the right price, this mobile service would be good value.  Perceived Enjoyment (ENJOY):...(1) I expect that using this mobile service would be enjoy-able. (2) I expect that using this mobile service would be pleasing. (3) I expect that using this mobile service would be entertaining.  Perceived Irritation (IRRITA): Three items were used: (1) I feel that this mobile service is irritating. (2) I feel that mobile coaching services are everywhere and, there is no need for another. (3) This mobile service looks annoying | The sample shows a homogeneous distribution between men (52%) and women (48%). 76% of participants are aged between 14 and 21 (N=86). More than 80% of participants (N=92) state that they smoke every day or nearly every day. Smokers can be grouped into three categories according to the number of cigarettes smoked per day: light smokers (N=32, 28%), medium smokers (N=40, 36%) and heavy smokers(N=41, 36%) (see Tables 2 and 3). Of the 113 participants in the study, only 37 stated that they intended to stop smoking in the next 12 months; 76 did not share that intention. However, the average intention to adopt the service does not differ significantly between the two groups, i.e. between people who say they want to stop smoking and the rest (F=2.62 (0)).  Among the people who intend to stop smoking in the next 12 months (N=37), most are Heavy Smokers (more than 10 cigarettes a day) and Light Smokers (fewer than 5 cigarettes a day). Medium Smokers (5 to 9 cigarettes a day) have proportionally less intention of giving up smoking; probably because, according to the arguments collected in the exploratory study they are less worried about their budget and their health, or are under less pressure from their entourage.  One of the barriers to adopting the service....is the lack of human contact in the coaching service. This is observed more with individuals over 18 than individuals under 18. Analysis of the variance shows a significant difference between the averages for the two groups of individuals. Of the 43 individuals who would not use this service because of the lack of human contact, 22 think it could help someone else to stop smoking.  Discussion: The intention to adopt the text message-based mobile coaching service to help stop smoking was tested with a mostly young target. This is precisely the group with the greatest risk of developing tobacco-related illnesses in the long term, since the lower the age at which people start smoking, the higher their risks of serious tobacco-related illnesses. Yet the study observed that these young smokers do not always intend to stop smoking in the short term. It is important to find innovative ways to motivate them to try and start the process of giving up smoking. With this in mind, mobile services can play an important role because young people are generally heavy users of cell phones and generally find new technologies attractive (Syed & Nurullah, 2011).  Conclusion: We obtained a fairly robust model in which vicarious innovativeness, social influence and the perceived enjoyment exert a positive influence on the intention to adopt the mobile coaching service. The perceived enjoyment has more influence on the intention to adopt the mobile coaching service in smokers who have never tried to stop smoking before than in smokers who have already tried to stop smoking. |
| Stephen Miller et al., 2020  Canada | Other: Self-report/self-assessment | Not specified, variable | Not specified, variable | Online | Not specified | Perceptions on the app and its utility were collected through a self- completed study questionnaire following completion of the Ada self-assessment.  In response to the Healthwatch Enfield report finding a significant factor of age in driving acceptability of symptom checkers, we explored this issue as a secondary question of interest.  Participants were asked to complete a paper questionnaire including their full name, date of birth, sex, and Likert-scale multiple choice questions on how likely they would be to recommend Ada, their ease of use, whether Ada provided helpful advice, whether they would use it again, and whether using Ada changed a decision about what to do. | Over a 3-month period, 523 patients participated. Most were female (n=325, 62.1%), mean age 39.79 years (SD 17.7 years), with a larger proportion (413/506, 81.6%) of working-age individuals (aged 15-64) than the general population (66.0%). Participants rated Ada’s ease of use highly, with most (511/522, 97.8%) reporting it was very or quite easy. Most would use Ada again (443/503, 88.1%) and agreed they would recommend it to a friend or relative (444/520, 85.3%). We identified a number of age-related trends among respondents, with a directional trend for more young respondents to report Ada had provided helpful advice (50/54, 93%, 18-24-year olds reported helpful) than older respondents (19/32, 59%, adults aged 70+ reported helpful). We found no sex differences on any of the usability questions fielded. While most respondents reported that using the symptom checker would not have made a difference in their care- seeking behavior (425/494, 86.0%), a sizable minority (63/494, 12.8%) reported they would have used lower-intensity care such as self-care, pharmacy, or delaying their appointment. The proportion was higher for patients aged 18-24 (11/50, 22%) than aged 70+ (0/28, 0%). |
| Steven C. Martino et al., 2019  United States | Other: Comments, navigation | Not specified | Not specified | Online | Not specified - Proponents of quality reporting frequently presume that more abundant information yields more informed patient choices and improved clinician–patient matching (Shi, Scanlon, Bhandari, & Christianson, 2017). But research suggests otherwise, even when people want the additional information.  Each year, one in five Americans seeks out a new physician, half for primary care and half for specialty treatment (Center for Studying Health System Change, 2012). How this choice plays out has important consequences. | After participants viewed the website and selected a clinician, they were transferred to a postchoice survey, which inquired about their experience on the website, their decision-making process, and their satisfaction with the choices available to them. | Introducing patient comments enhanced engagement with the quality information but led to a decline in decision quality, particularly the consistency of choices with consumers’ stated preferences. Labeling comments helped erase the decline in decision quality, although the highest percentage of preference-congruent choices was seen in the navigator arm. Engagement with the quality information and satisfaction with choices available were likewise highest in the navigator arm. Findings held for high- and low-skilled decision makers. Thus, navigator assistance may be a promising strategy for equitably promoting higher quality choices in information-rich contexts  Overall, patients tended to be satisfied with the website (75% said they would recom-mend the site to others who were choosing a doctor) and report that the metrics on the site were easy to understand (70%). However, less than half the sample (46%) said that it was easy to make trade-offs among the metrics included on the site and more than two thirds selected clinicians who were inconsistent with their stated preferences.  The design and content of the website had little evident impact on the scope of outcomes considered, ease of making trade-offs, and consumers’ ability to understand particular quality metrics (all p values for the omnibus test of cross-arm differences ≥0.13; see Table 3).  As we found in our previous research, however, introducing patient comments to the website—in any format—substantially enhanced patient engagement with the quality information in terms of time spent deliberating and the number of actions taken on the website. Tagging comments did not enhance engage-ment beyond what was observed in the conventional comments condition. Engagement was highest among participants in the navigator condition, who not only spent the most time deliberating and exploring the website but also expressed the highest satis-faction with the website.  tagging comments led to a decline in participants’ ability to find relevant information on the website (vs. having no comments or comments without labels), but having a navigator present more than compensated for this decrement. |
| StutiDang et al., 2017  Canada | Other: | 3 month study | 10 daily questions regarding their weight and HF symptoms for 3 months | Other: Mobile phone | Not specified | Usability, user experiences perspectives and recommendations | Forty-two participants aged 53.0 – 9.4 years (mean – standard deviation) were randomized to the mobile-monitoring intervention group. They included the following: 67% males, 76% White Hispanics, 21% African Americans, and 52% with high school education or less. Over the 3-month inter- vention period, 26 (62%) participants used the system over 50% of the time. Overall, on a 1.0–7.0 scale for both, program satisfaction scores were excellent (mean 6.84 – 0.46), and the usability ratings were all above 6.0. Comparing 1- to 3-month responses, there was a substantial increase in the percentage of participants who felt the system was easy to use after they had gotten used to it(84%vs.94%) and that navigating the system was not complicated (78% vs. 84%). Almost all participants said that the program made them feel more secure about their health and that they would stay enrolled in a program like this. None of them had used a similar system before. |
| Susan D. Newman et al., 2019  UK | Peer | Not specified - variable | Not specified - variable | Online | Social cognitive theory | Usability and acceptability of educational content and technology. | Participants were receptive and satisfied with the iPad and iTunes U platform and the video chat experience. Statements by our participants demonstrated a clear preference for interactive and multimedia platforms to promote engagement with educational materials. The use of FaceTime to facilitate contact between the participant and PN demonstrated satisfactory usability and acceptability. The hands-on evaluation process highlighted the need for consideration of connectivity for rural participants and assistive technology needs. |
| Susan E. Appling et al., 2016  United States | Patient | Not specified | Variable, contacted by navigator every 2 weeks or more if needed | Online | Poor patients are more likely than wealthier patients to have treatment delays or incomplete treatment, thus contributing to poorer survival | Not specified | Of the participants, 67% were minorities, mostly African American. Slightly more of the group randomized to the navigator team reported that the application was easy to use (77.6% vs 71.4%) and with higher confidence (71.4% vs 67.3%), but these differences were not statistically significant. |
| Tanya Millard et al., 2016  United States | Other: Combination of discussion boards, peer live chats/supports, information/resources | 7 weeks | Variable - encouraged to log onto the program for 90 min per week | Online | The online Positive Outlook Program was based on self-efficacy theory | Primary outcomes were evaluated at three time-points (baseline, post-intervention and 12-week’s post-intervention follow-up) and included HIV-related quality of life (PROQOL-HIV), outcomes of health education (HeiQ) and HIV specific self-efficacy (Positive Outlook Self-Efficacy Scale). | A total of 132 gay men with HIV in Australia were randomly allocated to the intervention (n = 68) or usual care control (n = 64) groups. Maximum likelihood marginal-linear modelling indicated significant improvement in the intervention group on the PROQOL-HIV subscales of body change (p = 0.036), social relationships (p = 0.035) and emotional distress (p = 0.031); the HeiQ subscales of health-directed activity (p = 0.048); constructive attitudes and approaches (p = 0.015); skill and technique acquisition (p = 0.046) and health service nav- igation (p = 0.008); and the Positive Outlook Self-Efficacy Scale on the subscales of relationships (p = 0.019); social participation (p = 0.006); and emotions (p = 0.041). Online delivery of self-management programs is feasible and has the potential to improve quality of life, self-management skills and domain specific self-efficacy for gay men with HIV. |
| Tuula Karhula et al., 2015  Canada | Other: Health coaching | Not specified | Contacted every 4-6 weeks by health coaches, required to self-monitor once per week | Hybrid | There is a strong will and need to find alternative models of health care delivery driven by the ever-increasing burden of chronic diseases | Primary outcome was health related quality of life (HRQL) measured by the Short Form (36) Health Survey (SF-36) and glycosylated hemoglobin (HbA1c) among diabetic patients. The clinical measures assessed were blood pressure, weight, waist circumference, and lipid levels. | A total of 267 heart patients and 250 diabetes patients started in the trial, of which 246 and 225 patients concluded the end-point assessments, respectively. Withdrawal from the study was associated with the patients’ unfamiliarity with mobile phones—of the 41 dropouts, 85% (11/13) of the heart disease patients and 88% (14/16) of the diabetes patients were familiar with mobile phones, whereas the corresponding percentages were 97.1% (231/238) and 98.6% (208/211), respectively, among the rest of the patients (P=.02 and P=.004). Withdrawal was also associated with heart disease patients’ comorbidities—40% (8/20) of the dropouts had at least one comorbidity, whereas the corresponding percentage was 18.9% (47/249) among the rest of the patients (P=.02). The intervention showed no statistically significant benefits over the current practice with regard to health-related quality of life—heart disease patients: beta=0.730 (P=.36) for the physical component score and beta=-0.608 (P=.62) for the mental component score; diabetes patients: beta=0.875 (P=.85) for the physical component score and beta=-0.770 (P=.52) for the mental component score. There was a significant difference in waist circumference in the type 2 diabetes group (beta=-1.711, P=.01). There were no differences in any other outcome variables. |
| V.C. Sánchez-Ortiz et al., 2011  Italy | Patient | 8 interactive sessions | Variable - "The support time varied depending on the extent to which the participant engaged with the support, ie, replied to the support emails." | Online | Cognitive behavioural therapy (CBT)delivered via CD-ROM or the internet has shown promise in the treatment of Bulimia Nervosa (BN), Binge Eating Disorder (BED)and Eating Disorders not Otherwise Specified (EDNOS) (1-5). | Interviews were semi-structured and the questions were open-ended to allow the researcher to explore important issues as they arose (9). A topic guide....included: layout/presentation of the package; access to treatment; expectations; support around the package and motivation; knowledge and relevance; process of change and confidence in recovery.  Additional information on RCT participants’ views and perceptions of iCBT was obtained through a questionnaire, sent to all RCT participants (N=64) who took up iCBT (defined as completing at least one session). The questionnaire was developed by two of the authors(VSO and CM) and consisted of statements rated on visual analogue scales and also open-ended questions about the programme.  The content of interviews were analyzed using thematic analysis. | Questionnaire responses echoed themes identified in the interviews. iCBT was received positively as a way of fitting treatment into busy lives. Comments on the layout of some of the package content and the practitioner support offered were identified as areas that could be modified or improved.  Participants provided detailed accounts of their experience of using iCBT. In-depth inter-views revealed five key themes, and some consisted of several categories.  1. Reasons for choosing this form of treatment  2. Experiences of treatment: - sub-themes - (I) Confidentiality/privacy; (II) Flexibility; (III) Ease of use; (IV) Feeling supported - including help with motivation; and (V) Content of programme  3. Impact of treatment: - sub-themes - (I) Expectations about outcome; (II) Effectiveness - Changes in ED symptoms; (III) Effectiveness - Other changes; and (IV) Tools for coping in the future  4. Comparison between iCBT and other forms of treatment: - sub-themes - (I) Counselling; (II) General Practitioner (GP); and (III) Other Forms of Self-help  5. Feedback: - sub-themes - (I) Timing; and (II) Other methods of support |
| Veronica P. S. et al., 2020  UK | Other: Self-monitoring, education | Not specified, variable | Not specified, variable | Other: App, text messaging | Self-Determination Theory | Percived usefulness, satisfaction,acceptability, intervention processes, resource management, and outcome effect variances | ThE CARE intervention was found useful and participants “felt empowered” utilizing the app. Fourteen participants (70%) reported high-intensity distress and negative impact on life from neuropathic pain, anxiety (55%), fatigue (50%), and depressive symptoms (35%). Self-awareness and self-regulation also improved. Modest results of acceptability, usability, and positive trends in the outcome measures suggest possible effects. |
| Xia Jin et al., 2019  Canada | Other: Web-based orders of at home test kits | Not specified | Not specified | Online | Not specified - With China’s explosive internet growth, activities such as socializing and partner seeking among men who have sex with men (MSM) has also become Web based through popular services such as Blued. This creates a new mode of health promotion with the potential to instantly reach large numbers of MSM, including those who rarely access traditional offline testing facilities. | A chi-squared trend test was used to assess the relationship between lifetime HIV testing volume and HIV prevalence. Logistic regression models were used to identify independent risk factors associated with two outcomes: (1) never having tested for HIV and (2) receiving an HIV-positive result. | Total of 879 individuals submitted Web-based requests for test kits. Their median age was 28 (interquartile range 24-34 years); 69.3% (609/879) had at least a college education, and 51.5% (453/879) had a monthly income between US $450 to $750; 77.7% (683/879) of the applicants submitted images of their test results, among whom 14.3% (98/683) had an HIV-positive result. Among the 42.9% (293/683) who were first-time testers, the HIV prevalence was 18.8% (55/293). Nearly three-quarters (71/98, 72.4%) of those with a positive test result were connected with a peer navigator and enrolled in treatment. Among the first-time testers, having multiple sexual partners (2-3 sexual partners: adjusted odds ratio [aOR] 2.44, 95% CI 1.08-5.50; 4 or above sexual partners: aOR 3.55, 95% CI 1.18-10.68) and reporting inconsistent condom use in the previous 3 months (aOR 7.95, 95% CI 3.66-17.26) were both associated with an HIV-positive result. An inverse dose response relationship between lifetime  HIV testing volume and HIV prevalence was also observed in this study (χ2 =55.0; P<.001). |
